# Preclinical pancreatic cancer mouse models for treatment with small molecule inhibitors: a systematic review and meta-analysis

**DOI:** 10.1038/s41598-025-25191-1

**Published:** 2025-10-27

**Authors:** Sophia Villwock, Yalda Mirzaei, Edgar Dahl, Julia Steitz

**Affiliations:** 1 Institute for Laboratory Animal Science, Medical Faculty, RWTH Aachen University, Aachen, Germany; 2 Institute of Pathology, Medical Faculty, RWTH Aachen University, Aachen, Germany; 3 Center for Integrated Oncology Aachen Bonn Cologne Düsseldorf (CIO-ABCD), Aachen, Germany; 4 Institute for Laboratory Animal Science Medical Faculty, RWTH Aachen University, Pauwelsstrasse 30, 52074 Aachen, Germany

**Keywords:** Cancer, Gastrointestinal cancer, Pancreatic cancer

## Abstract

Pancreatic cancer (PC) is an aggressive malignant disease with poor prognosis, often diagnosed late, progressing rapidly, and resistant to chemotherapy. Although small molecule inhibitors (SMIs) show promise in preclinical PC models, translation into clinics remains challenging. In this systematic review and meta-analysis, we screened literature according to predefined criteria to identify preclinical PC mouse models used for SMI therapy in primary tumors, assess reporting quality, and evaluate tumor reduction, heterogeneity and publication bias. Following a pre-registered PROSPERO protocol (CRD42022314932), literature searches in PubMed and Embase yielded 2972 articles, of which 297 were included for data extraction. Most studies used PDX models or MiaPaCa-2 and PANC-1 cell lines as heterotopic xenografts, with Foxn1^nu^ mice representing the predominant genetic background. Reporting quality, assessed using the ARRIVE guidelines, revealed substantial gaps, particularly in blinding (94% not reported), inclusion/exclusion criteria (49% not reported), and randomization (34% not reported). Meta-regression accounted for part of the observed heterogeneity and funnel plot asymmetry was consistent with publication bias. This study emphasized the large variability among preclinical PC mouse models used to investigate SMI candidates and the complexity of model choice highlighting the critical need for improved reporting practices to enhance reproducibility and reliability of preclinical tumor models.

**Supplementary Information:**

The online version contains supplementary material available at 10.1038/s41598-025-25191-1.

## Introduction

Pancreatic cancer (PC) is one of the deadliest human malignancies worldwide, ranked as 7th leading cause of cancer-related deaths with a median patient survival of 6–8 months post-diagnosis^1,2^. Due to its aggressive nature and intrinsic chemotherapy resistance, PC has extreme poor prognosis with a 5-year-survival rate of only ~ 11%^1,3^. Despite being the 12th most common malignancy worldwide, its high mortality rate emphasizes the critical need for improved prevention, diagnostics and treatment^2,4^. Currently, there are no pancreatic tumor-specific diagnostic tools available which impedes together with non-specific symptoms early diagnosis^4,5^. Consequently, the disease is typically diagnosed at advanced stage when metastases has already occurred^1,5,6^. Surgical resection of the tumor, accompanied by adjuvant chemotherapy, is currently the only potentially curative option. However, as 90% of patients relapse and succumb to the disease post-surgery^5^. Additionally, the effectiveness of chemotherapeutics is limited by dose-related toxicity and a lack of specificity toward cancer cells, highlighting the urgent need for alternative therapies^7^.

A new potential approach in PC patients are tumor-targeted therapies typically involving small molecule inhibitors (SMI) that have been evolved over the past 20 years and are being extensively studied^8,9^. These drugs are typically administered orally and are small enough (less than 500 Da) to permeate the plasma membrane, enabling interaction with intracellular targets such as cytoplasmic domains of surface receptors and other cancer cell components^8^. Compared to traditional chemotherapeutics, SMIs offer higher selectivity, efficacy, and tolerability, representing significant improvements in patient therapy and outcome^10^. Several phase II/III clinical trials are currently investigating various targeted therapy approaches for PC treatment^11^. However, no targeted therapies have yet become standard clinical care for PC patients as SMIs still face challenges such as low response rate, short response duration, toxicities, and resistance^10^. Furthermore, the enormous complexity of cancer diseases with intra- and intertumoral molecular and biological heterogeneity poses significant challenges for translating preclinical research into clinical application^12,13^. Therefore, robust preclinical research is essential for the optimal development of effective therapies, using a variety of models that collectively represent the biology and genetics underlying the therapeutic outcomes in specific cancers^12^. However, selecting the most appropriate model for a given research question can be challenging due to the diversity of available models like carcinogen-induced, genetically engineered, cancer cell line or patient-derived xenograft models each with its strengths and limitations^12,14^. Moreover, accurate reporting of animal studies is inevitable to ensure transparency and reproducibility of preclinical findings which is crucial for clinical translation^15^. The ARRIVE guidelines outline 10 essential items representing the minimum standards for reporting animal studies. Despite recognition, their impact on enhancing transparency has been limited, as evidence suggests that in vivo scientists may not fully recognize the significant comprehensive reporting, hindering potential improvements in reproducibility^15^.

Murine cancer models offer distinct advantages and disadvantages, and no single model is universally optimal. Thus, selecting the appropriate model for preclinical tumor research must be tailored to the specific research question. Conducting a systematic review before designing an experimental study is an invaluable step in determining the most suitable tumor model for the intended purpose. This study aims to provide a systematic review of published articles on preclinical PC mouse models using SMIs to assess reporting quality, tumor regression and investigates key study design variables critical for ensuring reproducibility in future preclinical studies.

## Results

### Study selection and search results

**Fig. 1:**
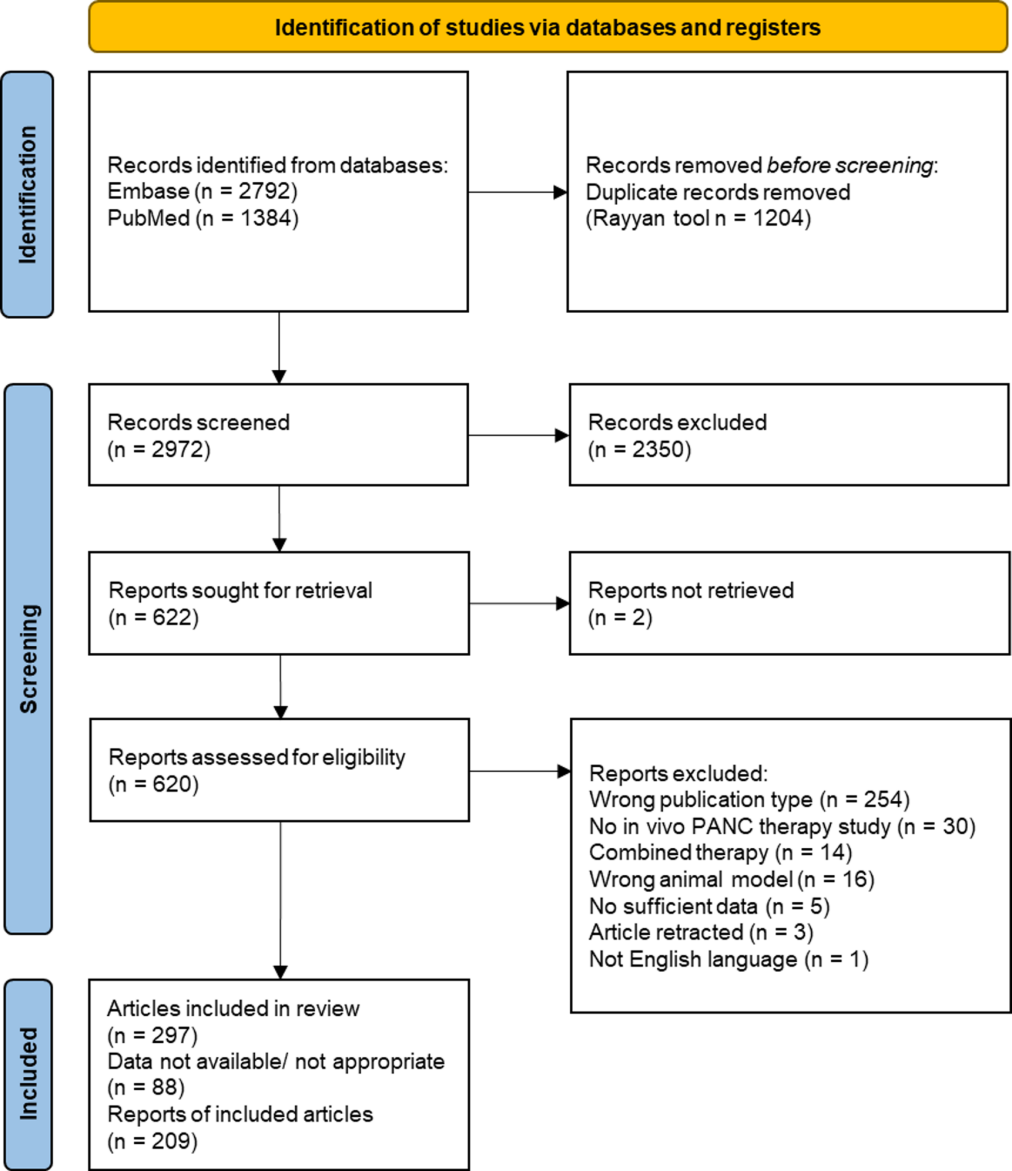
Flow diagram of the study selection from record identification over title and abstract following full-text screening to studies included for quantitative synthesis adapted from PRISMA^16^.

The systematic search yielded 4176 articles, with 1384 from Pubmed and 2792 from Embase (Fig. 1). After uploading to the Rayyan tool and removing 1204 duplicates, 2972 articles were screened for eligibility based on titles and abstracts. 2350 articles were excluded, leaving 620 for full-text screening according to predefined inclusion and exclusion criteria. During study selection and data extraction, the eligibility criteria were adjusted to exclude natural compounds, their derivatives, new compounds with not yet defined clear targets from interventions, compounds with originally other purposes than cancer and co-cultured cancer cells. Ultimately, 297 articles met the criteria for qualitative analysis. These articles often included multiple interventions and mouse models, resulting in 756 individual studies. Of the 297 articles, 88 were excluded from the meta-analysis due to incomplete or inappropriate data, such as missing animal numbers or non-relevant primary outcomes (e.g. pancreas weight from transgenic studies). Two of these articles contained data discrepancies (see SI reference list items 21 and 177). From the remaining 209 articles, comprising 485 individual studies, were included in the quantitative meta-analysis; since some studies reported more than one primary outcome, they were counted only once here but analyzed separately in the meta-analysis.

### Article characteristics

For each of the 756 studies from 297 articles, we collected detailed data on study design (groups, randomization, blinding, tumor model), mouse characteristics (background, sex, age, weight), and interventions (SMI category, dose, application). The following chapters summarize these data to describe study characteristics and reporting quality.

**Fig. 2:**
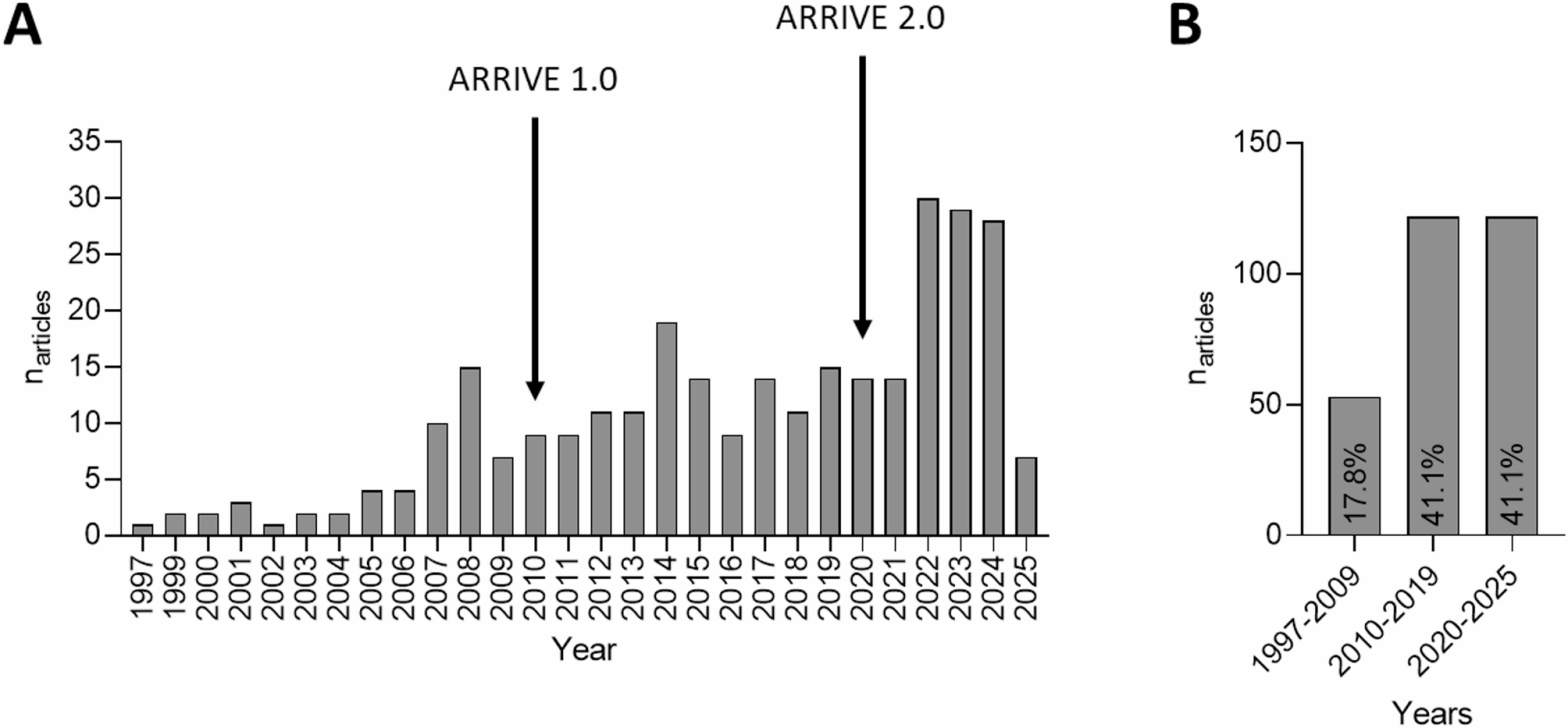
The distribution of the included articles over the years shows an increasing number of publications per year from 2007 onwards. (**A**) The number of articles published per year from 1997 to January 2025, when the systematic search was conducted. (*n* = 297) The arrows indicate the year when the ARRIVE guidelines were published. (**B**) Articles published in three time periods: 1997–2009: 53 articles (17.8%), 2010–2020: 122 articles (41.1%), and 2020–2025: 122 articles (41.1%). The years were trichotomized based on the years when the first ARRIVE Essential 10 guidelines (2010) and the updated ARRIVE 2.0 guidelines (2020) were published^15,17^.

The 297 articles were published across 112 different journals. The journals with the most publications (≥ 10 articles) were: Cancer Research (*n* = 18, 6.1%), Clinical Cancer Research, (*n* = 21, 7.1%), Molecular Cancer Therapeutics (*n* = 27, 9.1%), Oncotarget (*n* = 13, 4.4%), and PLOS ONE (10, 3.4%) (supplemental information (SI) Table 1). From 1997 to 2006, fewer than five articles were published annually on this topic, but since 2007, at least nine articles were published each year. For 2025, only articles published up to January 23rd (time of literature search) were included, resulting in seven articles for 2025 (Fig. 2A). Between 1997 and 2009 (13 years), 17.8% of articles were published. While in 10 years, between 2010 and 2019, 41.1% were published, in only 5 years, between 2020 and 2025, the exact same number of articles were published reflecting the increasing publication rate over the years. (Fig. 2B) Transgenic models (26 articles, 37 individual studies) were included in the systematic review but excluded from meta-analysis due to the absence of primary tumor regression outcomes. Metastatic models (14 articles, 28 individual studies) were initially addressed in the research question but were ultimately insufficient for consideration in meta-analysis.

### Characteristics of articles included in meta-analysis

For the meta-analysis, 209 articles comprising 485 individual studies were included. To explore potential sources of heterogeneity, meta-regression was conducted on several characteristics, including sex, transplantation type, tumor graft, tumor cell/tissue application route, genetic background of mice, injected tumor cell/tissue type, SMI category, drug application route, randomization, blinding, and baseline tumor size. Further details on these characteristics based on the 485 individual studies are provided in Table 1. Details on the mouse strains, here represented as genetic background, are summarized in SI Table 3.

**Table 1:** Overview of characteristics (sex, transplantation type, tumor graft, tumor cell/tissue application route, genetic background of mice, injected tumor cell/tissue type, SMI category, drug application route) of the 485 individual studies included for meta-analysis. The absolute number (n) always adds up to a total of 485. The relative number (%) usually adds up to 100%, deviations are due to rounding. The abbreviation n.m. Means ‘not mentioned’.

Characteristic	Subgroup	*n* (%)
Sex	Female	235 (48.5)
	Male	122 (25.2)
	Female/male	12 (2.5)
	n.m.	116 (23.9)
Transplantation	Heterotopic	382 (78.8)
	Orthotopic	94 (19.4)
	Metastatic	9 (1.9)
Tumor graft	Xenograft	363 (74.8)
	Patient-derived xenograft	75 (15.5)
	Syngraft	45 (9.3)
	Allograft	1 (0.2)
	n.m.	1 (0.2)
Application route tumor cells/tissues	Subcutaneously (s.c.)	380 (78.4)
	Intrapancreatic (i.panc.)*	94 (19.4)
	Intrasplenic (i.splen.)	5 (1.0)
	Intraperitoneally (i.p.)	3 (0.6)
	Peripancreatic (peri.panc.)	2 (0.4)
	Intravenously (i.v.)	1 (0.2)
Genetic background	Foxn1^nu^	300 (61.9)
	Prkdc^scid^	68 (14.0)
	Prkdc^scid^, Il2rg^tm1WjI^	56 (11.5)
	Wild type (wt)	44 (9.1)
	Prkdc^scid^, Lyst^bg^	3 (0.6)
	Prkdc^em26Cd52^Il2rg^em26Cd22^	3 (0.6)
	Ido1^tm1Alm^	2 (0.4)
	Pde6b^rd1^	2 (0.4)
	PNP	2 (0.4)
	Rag2^tm1Fwa^II2rg^tm1Rsky^	2 (0.4)
	n.m.	3 (0.6)
Tumor cell/tissue type	Patient-derived	75 (15.5)
	MiaPaCa-2	68 (14.0)
	PANC-1	63 (13.0)
	Panc-02	29 (6.0)
	AsPC-1	27 (5.6)
	BxPC-3	23 (4.7)
	L3.6pl	23 (4.7)
	Capan-1	18 (3.7)
	HPAF-2	13 (2.7)
	SW-1990	13 (2.7)
	others**	132 (27.2)
	n.m.	1 (0.2)
Small molecule inhibitor (SMI) category	Selective non-kinase SMI	234 (48.2)
	Selective intracellular kinase SMI	161 (33.2)
	Selective receptor-related kinase SMI	53 (10.9)
	Multikinase SMI	21 (4.3)
	Others***	16 (3.3)
Application route drug	i.p.	231 (47.6)
	Per orally (p.o.)	218 (44.9)
	i.v.	9 (1.9)
	s.c.	4 (0.8)
	Retroorbitally (r.o.)	2 (0.4)
	Intratumorally (i.t.)	1 (0.2)
	n.m.	20 (4.1)

*Applications mentioned in pancreas tail (n = 46, 9.5%), body (n = 2, 0.4%), head (n = 3, 0.6%), and subcapsular (n = 1, 0.2%). **Cell lines represented with less than 10 individual studies. ***SMIs that are either selective receptor-related and intracellular kinase SMIs (n = 10, 2.1%) or selective intracellular kinase and non-kinase SMIs (n = 6, 1.2%).

*Applications mentioned in pancreas tail (*n* = 46, 9.5%), body (*n* = 2, 0.4%), head (*n* = 3, 0.6%), and subcapsular (*n* = 1, 0.2%). **Cell lines represented with less than 10 individual studies. ***SMIs that are either selective receptor-related *and* intracellular kinase SMIs (*n* = 10, 2.1%) or selective intracellular kinase *and* non-kinase SMIs (*n* = 6, 1.2%).

### Reporting quality assessment of the included articles

**Fig. 3:**
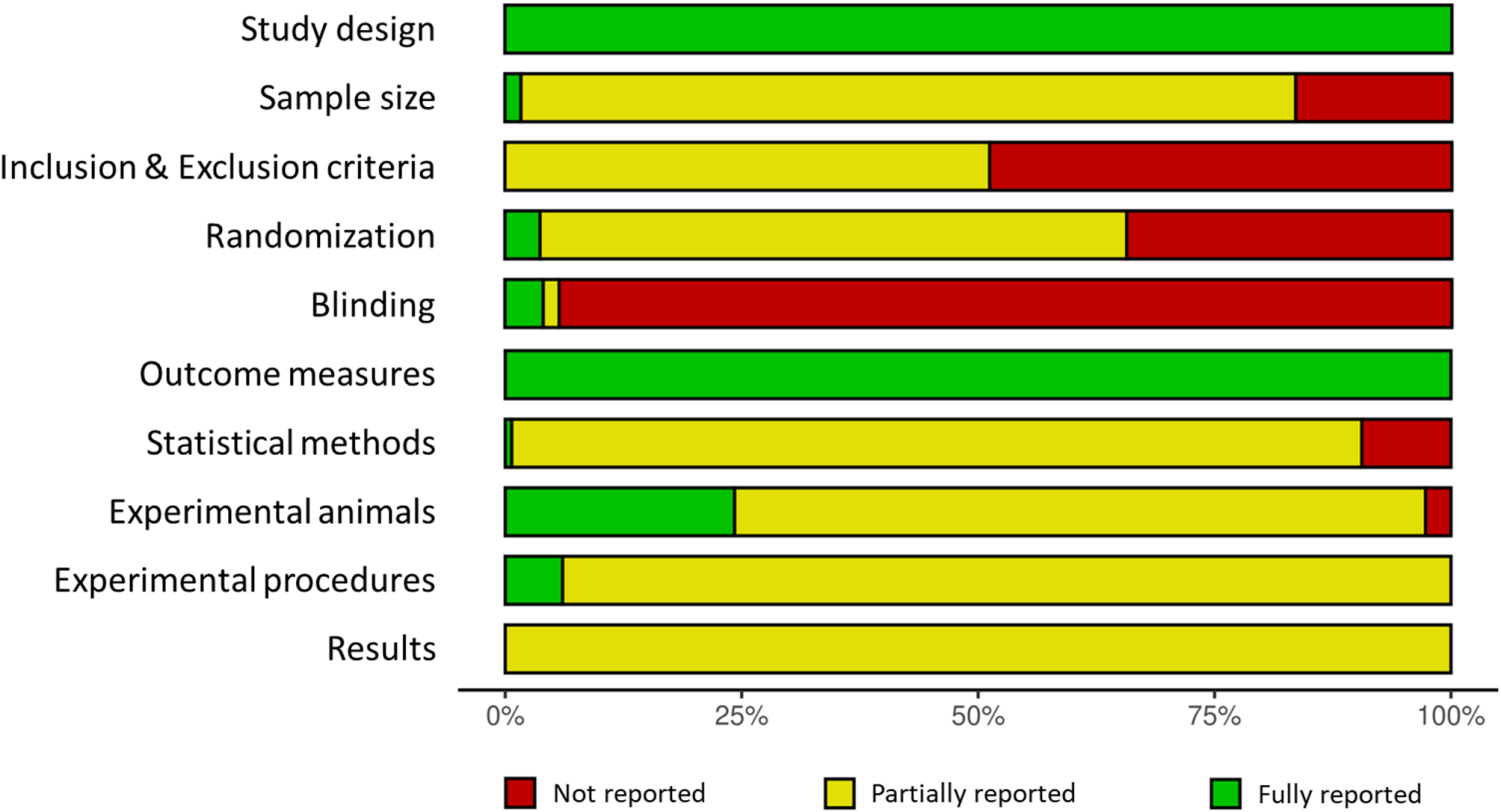
Summary plot: The reporting quality assessment of the 297 included articles was conducted based on the ARRIVE Essential 10 guideline 2.0 and visualized using the robvis tool^18^. Red represents unreported information, yellow partially reported information and green fully reported information according to the guidelines.

Reporting quality was assessed using the ARRIVE Essential 10 guidelines 2.0^15^. Details are in SI Table 2, and an overview is shown in Fig. 3. All 297 articles fully reported study design and outcome measures but only partially reported criteria for results. While descriptive statistics and measures of variability were provided in all articles, effect sizes and confidence intervals were not. Only five articles (2%) reported all criteria for sample size: two using group size calculations based on previous or preliminary studies, and three based on power calculations. Of the remaining articles, 243 (82%) partially reported sample size criteria, specifying the number of animals but not the method for determination. The remaining 49 articles (16%) did not provide sample size information.

None of the 297 articles fully reported inclusion and exclusion criteria. Half of the articles (152, 51%) partially reported these criteria, typically mentioning exclusion only when animals were removed due to early death or reaching humane endpoint). The other half (145, 49%) did not report any information on inclusion or exclusion criteria.

Randomization was not reported in 102 articles (34%). Of 195 articles (66%) that mentioned randomization, only 11 articles (4%) fully described the randomization process, with e.g. the random number table method or digital randomization tools. One article explicitly stated no randomization was performed. Blinding was not reported in 280 articles (94%). Five articles (2%) reported that blinding was performed without further details. Twelve articles (4%) fully reported blinding performance (e.g. blinding of personnel in different stages), whereas seven of these articles stating no blinding was performed. No further details were provided on measures to reduce performance bias, such as random housing, blinding of animal caretakers, or awareness of group allocation during data analysis. Regarding statistical methods, two articles (1%) fully reported all details, 267 articles (90%) partially reported them, and 28 articles (9%) did not provide any statistical information. For experimental animal criteria (species, sex, age, weight, developmental stage), 72 articles (24%) fully reported all details, while 217 articles (73%) reported them partially. In general, age was reported in most articles while weight was less reported. For experimental procedures (timing, intervention frequencies, acclimatization), only 18 articles (6%) fully reported these criteria, with 279 articles (94%) providing partial information. While timing and intervention frequencies were usually reported, acclimatization periods were often omitted. All results were partially reported as ARRIVE guidelines expect besides descriptive statistics, which are always reported, effect size and confidence interval. However, these two measures were not reported in any of the articles.

### Outcome measures and exploration of heterogeneity

Primary tumor volume, weight and area regression were three specific types of outcomes in the included studies and were classified and assessed separately.

#### Overall effect of small molecule inhibitor therapy on primary tumor regression

**Fig. 4:**
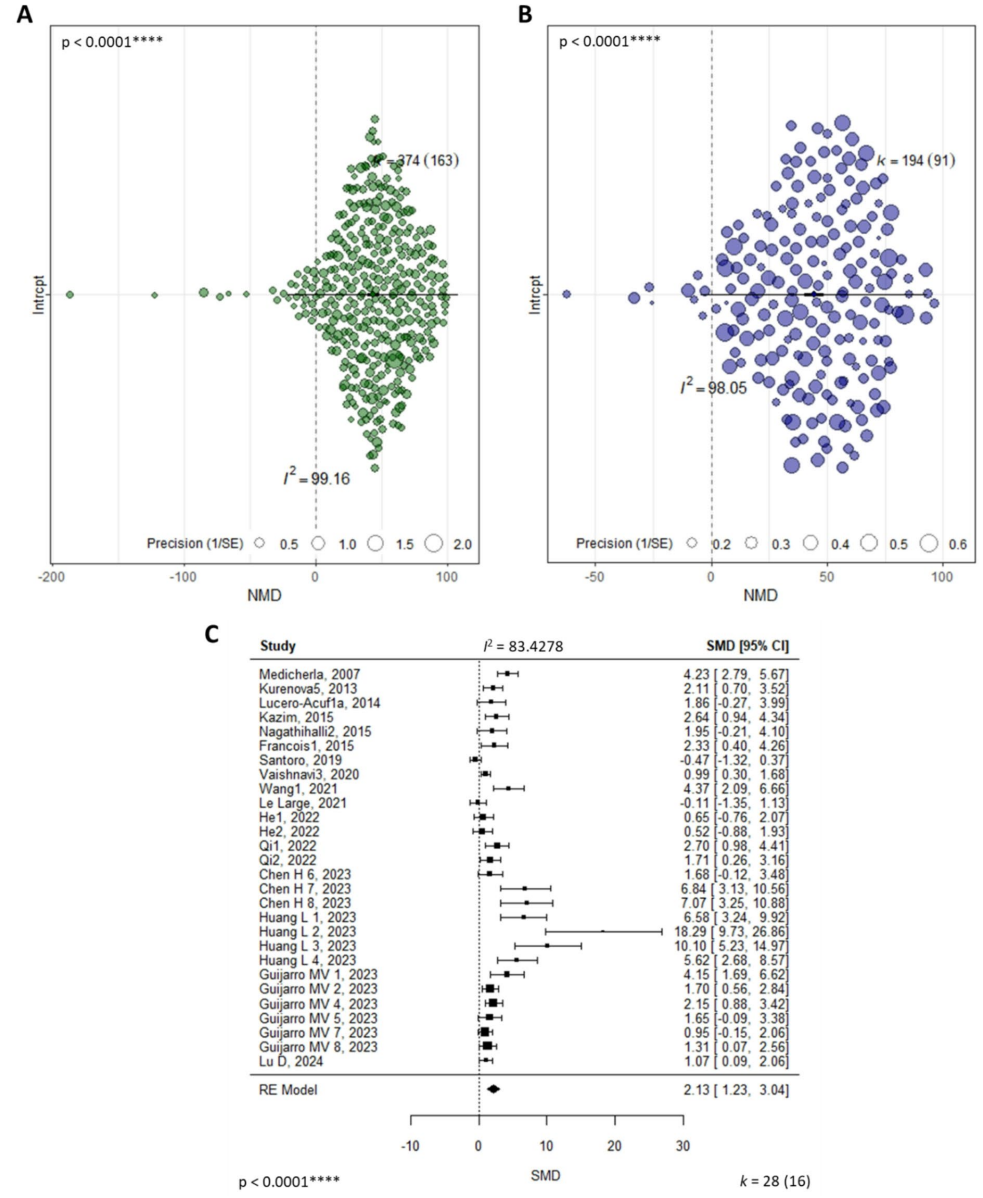
Meta-analysis results for primary tumor regression: (**A**) Tumor volume orchard plot, (**B**) Tumor weight orchard plot, (**C**) Tumor area forest plot. On each plot, the thick black line indicates 95% confidence intervals (CI), the extended thin black line indicates 95% prediction intervals (PI), and the midpoint is denoted by a square indicating the pooled normalized (**A**, **B**) and standardized (**C**) mean difference (NMD and SMD respectively) effect estimate. Each circle represents one comparison, and its diameter designates the weight each comparison carries in the pooled effect size based on precision, meaning the larger the sample size the comparison has, the more precise it is and the higher its weight in the pooled effect. I^2^ reports the quantitative value for heterogeneity (A 99.16%, B 98.05% and C 83.43%) and the p-value (p) for the test of heterogeneity was statistically significant (****p < 0.0001). The number of comparisons included in the meta-analysis is labelled by k with the number of individual studies labelled in brackets.

A total of 596 individual comparisons from 209 articles assessed primary tumor regression based on tumor volume (*n* = 374), tumor weight (*n* = 194) or tumor area (*n* = 28). Tumor volume (V) was typically measured with caliper and calculated using the formula $\:\text{V}=0.5\text{*}\text{L}\text{*}{\text{W}}^{2}$ (L = tumor length, W = tumor width). As shown in Fig. 4A and B, the pooled effect size was statistically significant in favor of using SMIs to reduce tumor volume and weight, with reductions of over 40% (tumor volume: NMD 44.05%, 95% CI [39.38–48.72], *p* < 0.0001, *n* = 374, tumor weight: NMD 44.27%, 95% CI [40.13–48.42], *p* < 0.0001, *n* = 194). However, a random-effects model showed considerable heterogeneity (I^2^) between the studies for both outcomes (tumor volume: I^2^ = 99.16%, *p* < 0.0001, tumor weight: I^2^ = 98.05%, *p* < 0.0001). The analyses revealed that most of the variability originated from differences between studies, with 60.5% of the variance in tumor volume and 78.1% in tumor weight attributable to inter-study heterogeneity. Prediction intervals (PI) also indicated high heterogeneity, with ranges crossing the no-effect line, suggesting both potential negative and positive effect sizes. For tumor volume, the PI ranged from − 20.88 to 108.96, and for tumor weight, it spanned from − 4.67 to 93.21. The pooled effect size for tumor area (Fig. 4C) was statistically significant in favor of using SMIs to reduce the tumor area (SMD 2.13 (SE = 0.46); 95% CI [1.23–3.23-04], *p* < 0.0001, *n* = 28), though considerable heterogeneity was observed (I^2^ = 83.43%, *p* < 0.0001), with PIs ranging from negative to positive effect sizes (PI [−1.30; 5.57]).

#### Subgrouping effects on heterogeneity of small molecule inhibitor therapy effects on primary tumor volume and weight regression

Subgroup analyses were conducted for tumor volume, weight, and area outcomes using uni- and multivariable meta-regression to test whether variation could be explained by study design (sex, genetic background, transplantation, graft type, cell/tissue source, application route, baseline tumor size), risk-of-bias factors (randomization, blinding), and drug-related variables (SMI category, drug application route) (SI Table 4).

**Fig. 5:**
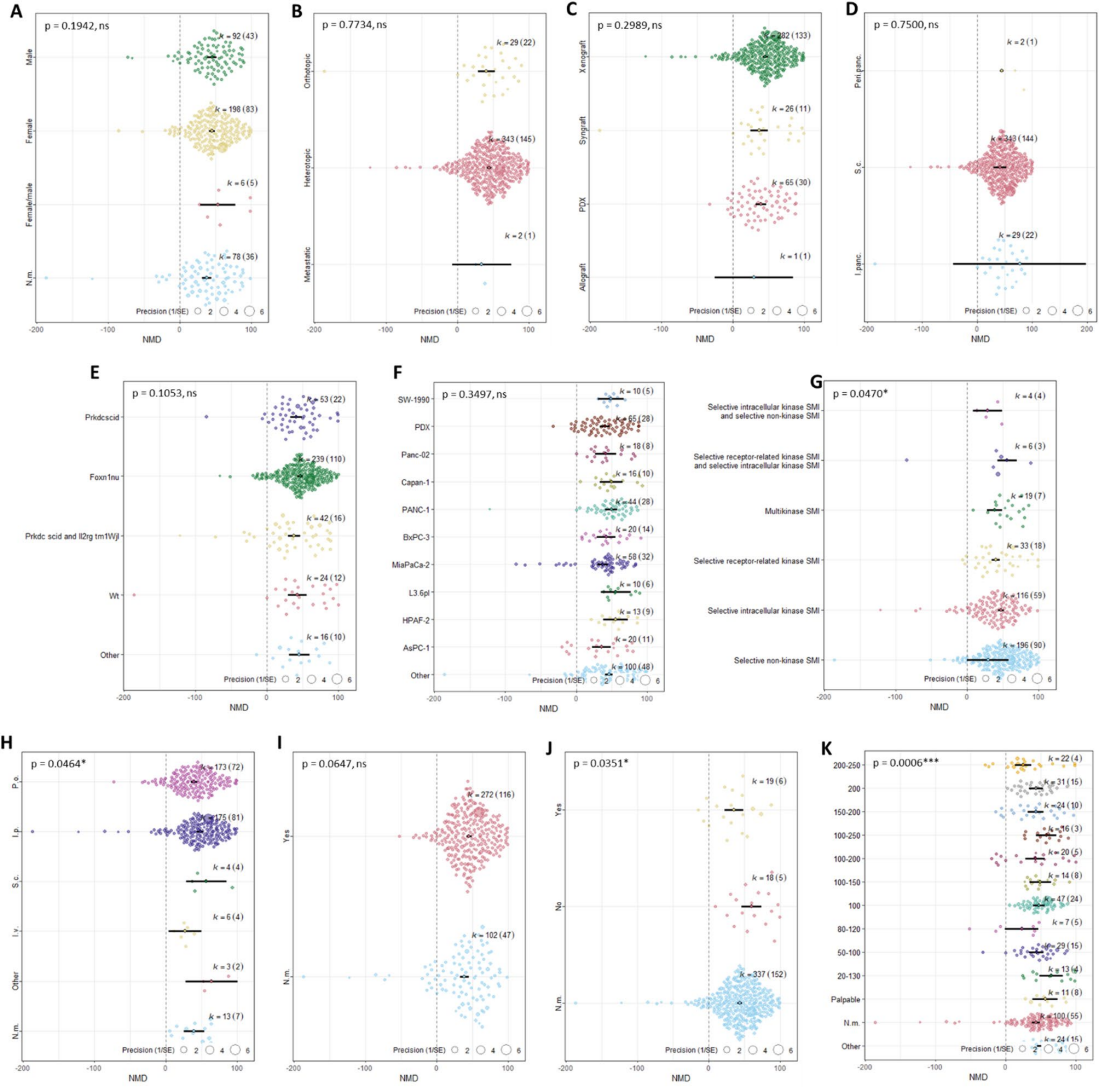
Orchard plots of univariate meta-regression analysis for primary tumor volume regression based on (**A**) sex, (**B**) transplantation, (**C**) tumor graft, (**D**) tumor cell/tissue application route, (**E**) genetic background of mice, (**F**) injected tumor cell/tissue type, (**G**) inhibitory category (‘other’ defined here as selective receptor-related kinase and intracellular SMI and selective intracellular kinase and non-kinase SMI), (**H**) drug application route (‘other’ defined here as i.t., i.v., r.o., s.c.). (**I**) randomization, (**J**) blinding, and (**K**) baseline tumor size. The thick black line indicates 95% confidence intervals (CI). Each circle represents one comparison, and its diameter designates the weight each comparison carries in the pooled effect size based on precision. The number of comparisons included in each subgroup of interest is labelled by k with the number of individual studies labelled in brackets. P-values (p) are presented with indication of significance (> 0.05 not significant (ns), ≤ 0.05*, ≤ 0.001***).

For tumor volume (k = 374), the multivariable model explained 14.4% of variance (R² = 14.43%; F(50,323) = 2.07, *p* < 0.0001), though residual heterogeneity remained high (I² = 87.6%). Absence of blinding inflated effects, smaller baseline tumors predicted larger responses, and strong effects were seen for HPAF-2, Panc-02, and PANC-1 (L3.6pl borderline). Selective receptor-related kinase inhibitors and oral application showed reduced efficacy. Univariate models supported these patterns but most single moderators explained little variance; the most informative were baseline tumor size (R² = 8.66%, *p* = 0.0006) and blinding (*p* = 0.035) (Fig. 5).

For tumor weight (k = 194), the multivariable model explained 32.9% of variance (R² = 32.89%; F(40,153) = 2.28, *p* = 0.0002), with residual heterogeneity remaining (I² = 66.0%). Stronger effects were observed in male animals, and in PANC-1 and MiaPaCa-2 tumors. Inhibitor class was not significant overall, and adding randomization, blinding, or baseline tumor size did not improve fit. Univariate models confirmed sex and cell line effects, and identified drug application route as a major determinant: intraperitoneal and oral delivery were linked to larger responses (SI Fig. 2).

For tumor area (k = 28), no moderators were significant in either multivariable or univariate analyses (all *p* > 0.2), and heterogeneity persisted (SI Table 6). Owing to small sample size and sparse subgroup representation, these findings should be interpreted with caution.

### Assessment of publication bias

**Fig. 6:**
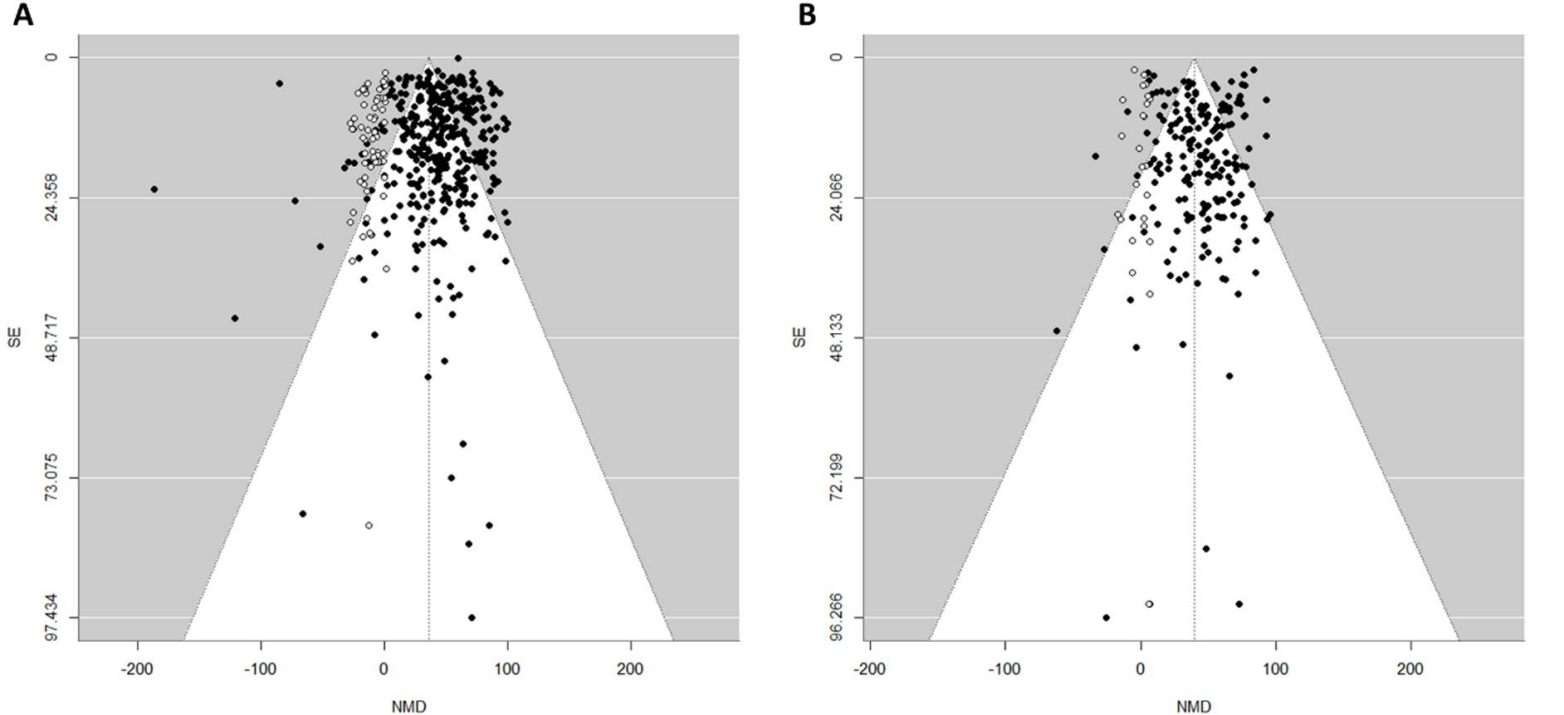
Funnel plots and trim-and-fill method of small molecule inhibitor therapy outcomes on pancreatic cancer. (**A**) Primary tumor volume regression, and (**B**) primary tumor weight regression. The black dots indicate the observed studies and the white dots the imputed missing studies by the trim-and-fill method (n_A_ = 65 and n_B_ = 25). Due to the high degree of heterogeneity and the overlap of imputed studies, the trim-and-fill method was only able to visualize and fill in a portion of the missing studies in the funnel plot. The x-axis shows the observed outcome as normalized mean difference (NMD) with the y-axis representing the standard error (SE). The two diagonal lines in either side of the plot represent the 95% confidence intervals. The dashed vertical line indicates the adjusted effect size including potentially imputed studies under the random effects model: NMD_A_ = 35.75%, and NMD_B_ = 39.49%.

For primary tumor volume and weight regression datasets, visual asymmetry in the funnel plots suggested potential bias in the findings (SI Fig. 3). The Egger’s test for primary tumor volume regression did not indicate significant funnel plot asymmetry (t(372) = − 1.68, *p* = 0.094), with the intercept estimate at 48.18 (95% CI: 42.49–53.88). However, the trim-and-fill method suggested as many as 65 potentially missing studies on the left side of the funnel, consistent with small-study effects (Fig. 6A). For primary tumor weight regression, Egger’s test again did not detect significant bias (t(192) = − 0.31, *p* = 0.759), with an intercept estimate of 45.49 (95% CI: 39.17–51.81). The trim-and-fill method imputed 25 missing studies on the left side of the funnel (Fig. 6B). The efficacy estimates for both regressions changed after the imputed studies were added, though SMI therapy still significantly favored the control. For tumor volume regression, the adjusted mean effect decreased to 35.75% (95% CI: 32.53–38.98, *p* < 0.0001), while for tumor weight regression, it was slightly attenuated to 39.49% (95% CI: 35.74–43.24, *p* < 0.0001; k = 219). Despite these adjustments, high heterogeneity remained in both datasets (tumor volume regression: I² = 95.9%, τ² = 923.0; tumor weight regression: I² = 85.8%, τ² = 499.6). Therefore, the adjusted intervention effect should be interpreted cautiously, as the inclusion of imputed studies likely contributed to the increased overall heterogeneity. Small-study effects for tumor area regression were explored descriptively using funnel plots (SI Fig. 3), which showed visual asymmetry. However, formal tests (Egger’s regression; trim-and-fill) are unreliable here because the number of independent studies is modest (~ 16), heterogeneity is high (I² = 83.43%), and multiple effects per study violate independence conditions that reduce power and can bias asymmetry estimates. We therefore treat the funnel plot as descriptive only and avoid attributing asymmetry to publication bias.

## Discussion

For pancreatic cancer (PC) no specific tools exist and curative approaches show limited effects. Therefore, there is an urgent need for advancements in prevention, diagnostics, and treatment^1,2,4–6^. Small molecule inhibitors (SMIs) offer a promising alternative for targeted therapy in PC, however, translating preclinical findings into successful clinical applications remains a significant challenge^10,12,13^. Robust preclinical cancer research is therefore essential and relies on a variety of animal models, each with unique advantages and disadvantages, making the selection of the most appropriate model for a specific purpose challenging^12,13^. To address this, we systematically reviewed published studies on small molecule inhibitors (SMIs) in preclinical PC mouse models, assessed the reporting quality in these articles, and conducted a meta-analysis to identify potential sources of heterogeneity that could impact the reproducibility of future preclinical studies.

Due to distinct differences between preclinical and clinical systematic reviews and meta-analyses such as higher numbers of included studies, smaller sample sizes, and higher variability in the preclinical compared to the clinical field, the synthesis of preclinical data may not always provide sufficient evidence for meta-analyses or firm conclusions^19^. Clinical systematic reviews and meta-analyses primarily aim to inform late-phase clinical trials, clinical practice, and guidelines, while also exploring heterogeneity within clinical populations. In contrast, preclinical systematic reviews and meta-analyses focus on investigating translational failures, explaining discrepancies, exploring both internal and external validity, heterogeneity, and guiding future preclinical research, early-phase clinical trials, and 3Rs (Replacement, Reduction, and Refinement) decisions^19^.

Here, we were able to systematically review available articles between 1997 and 23rd January 2025 according to predefined eligibility criteria. From 2010, when the first ARRIVE guideline^17^ was published, the publication rate of the included articles was three-fold higher than from 1997 to 2009, possibly due to increasing research interest in the field of PC and the urgent need for improvement of diagnostic and therapeutic tools, especially alternatives such as SMIs in the last decades^1,7–9^. This systematic review showed that most studies used female Foxn1^nu^ mice and predominantly applied heterotopic xenograft models, with patient-derived xenografts (PDX) being most common, followed by subcutaneously established MiaPaCa-2 and PANC-1 cell line xenografts. Both cell lines originate from pancreatic ductal adenocarcinomas (PDACs), the most common form of pancreatic tumors (85%)^1,4,20^. Both have a mutation for the proto-oncogene KRAS, which is the most common event in pancreatic cancer initiation and progression (~ 90%) of PDACs and can reflect human disease progression^20,21^. Since 2022, we observed a shift toward PDX models, reflecting a move to systems that more closely mimic patient’s tumor biology, preserving genetic and morphological characteristics. As highlighted in recent reviews^22,23^, model selection should be guided by the research question. PDX models may be preferable for testing new therapies, studying treatment resistance and advancing personalized medicine approaches and identifying biomarkers, whereas cell line-based models are better suited for early-stage efficacy screening. Most commonly, selective non-kinase or selective intracellular kinase SMIs (categorized according to Liu et al.^10^ were used and applied usually per orally or intraperitoneally.

The reporting quality was assessed according to the ARRIVE Essential 10 guidelines 2.0^15^, revealing concerns ranging from low to high, which could potentially compromise the internal validity of the included articles. Internal validity refers to the extent to which observed findings accurately reflect the true effects, excluding methodological errors^24^. These guidelines outline the minimum essential information required for reporting animal studies, which can significantly influence experimental outcomes and support transparency and reproducibility^15^. The most prominent reporting gaps in the included articles concerned measures to reduce bias, including selection, performance, detection, and attrition biases. While some articles mentioned randomization during group selection, the specific methods used were rarely described. Likewise, blinding procedures were rarely reported. Although incomplete reporting does not necessarily imply that these measures were not implemented, existing literature indicates that the inclusion of these measures is associated with substantially lower effect sizes^25^. Furthermore, other critical information that could impact effect sizes and heterogeneity of synthesized data in this study, such as sample size, statistical methods, experimental animals, procedures, and results, was often incomplete or entirely missing in the included articles. These omissions suggest poor internal validity, a finding consistent with literature, which frequently reports inadequate internal validity in animal studies. Already a decade ago, researchers were advocating for improvements in this area^25–27^. This suggests that while the ARRIVE guidelines have led to some improvement in the reporting quality of animal studies (SI Fig. 1), further enhancements are necessary, and greater emphasis on the importance of comprehensive reporting is critical. As recommended by ARRIVE 2.0^15^, key methodological measures should be reported in detail, specifying not only that they were applied but also how they were conducted, to enable rigorous assessment of study quality and reproducibility. Although the ARRIVE guidelines have been available for over a decade^17^, their inconsistent enforcement has led to persistent gaps in reporting. Recent evidence shows that requiring ARRIVE compliance in journal submission processes and explicit referencing by authors substantially improves reporting quality. Structured enforcement mechanisms, rather than geographic or bibliometric factors, appear most effective in raising reporting standards^28^. Strengthening adherence to these guidelines will improve the reproducibility and reliability of antitumor animal studies, thereby facilitating their translation from preclinical research to clinical trials^26,27^.

In the meta-analysis, primary pancreatic tumor regression following SMI therapy was assessed based on tumor volume, weight, and area. Across 596 individual comparisons from 209 articles, SMIs were associated with statistically significant reductions in tumor volume and weight of over 40%, as well as a significant decrease in tumor area. However, these findings were accompanied by considerable heterogeneity (I² >83% for all outcomes), indicating substantial variability in SMI efficacy across studies. Prediction intervals crossed the line of no effect, suggesting that both positive and negative effect sizes are possible. As tumor volume was typically measured with calipers and calculated using the formula V = 0.5 × L × W², measurement bias may also contribute to variability. Consistent with prior literature, such heterogeneity is common in preclinical research and is often linked to methodological limitations and risks of bias^25,29,30^. Therefore, prediction intervals may be more informative for illustrating the potential range of effects rather than for reliably predicting future outcomes.

To explore sources of heterogeneity, we conducted subgroup and meta-regression analyses. For tumor volume (k = 374), ~ 14% of variance was explained, with absence of blinding, smaller baseline tumors, and specific cell lines (HPAF-2, Panc-02, PANC-1, borderline L3.6pl) associated with stronger effects, while selective receptor-related kinase inhibitors and oral administration showed reduced efficacy. For tumor weight (k = 194), ~ 33% of variance was explained, with stronger effects in male animals and in PANC-1 and MiaPaCa-2 tumors; intraperitoneal and oral drug administration were also linked to larger responses. For tumor area (k = 28), no moderators were significant, likely due to small sample size. Overall, some study design and biological factors contributed to variability, but much remained unexplained. Key factors such as cell number, media, and SMI dosage may also contribute, but limited reporting prevented post-hoc analyses.

Funnel plot analyses suggested asymmetry for tumor volume and weight datasets, consistent with potential publication bias. Although Egger’s tests did not detect significant asymmetry, the trim-and-fill method imputed 65 and 25 missing studies, respectively, which reduced effect sizes (tumor volume: NMD = 35.75%; tumor weight: NMD = 39.49%) but left them statistically significant, while heterogeneity increased. Such patterns likely reflect selective reporting, with statistically significant positive results more likely to be published than negative or neutral findings^29,31–33^. The failure to publish neutral or negative findings poses a significant issue for research progress, as it leads to repeated failures and complicates the identification of suitable animal models. It could also lead to unnecessary animal use, violating the 3Rs principle. Furthermore, publication bias may inflate therapeutic effects and lead to unreliable conclusions^31,32^. Together with the limited generalizability and lack of standardized protocols in preclinical studies compared to clinical trials^26,29,32,34^., these findings should be interpreted with caution.

This work provided an overview of SMIs in PC in a variety of murine models and their impact on tumor regression. However, our investigation has limitations. It is recommended that two independent reviewers be involved in both study selection and data extraction processes^29,34^. In our study, two independent reviewers performed study selection during title and abstract screening while full-text screening and data extraction were carried out by a single reviewer. Any queries were discussed with a second or third reviewer.

The subgroups of genetic background and SMI category were manually assessed through literature research and to the best of our knowledge. For genetic background, we relied on the mouse strain information provided by the articles. However, authors often did not specify the exact mouse strain used based on international nomenclature. Therefore, we classified the mouse strains based on certain characteristics (genetic background, type of genetics, hair type, T cells, B cells, NK cells) and grouped them accordingly. This approach may introduce potential bias into the genetic background subgroup. Following Liu et al.^10^, we categorized the SMIs into four groups: multikinase, selective non-kinase, selective intracellular kinase, and selective receptor-related kinase SMIs. While this categorization is broadly comprehensive, alternative classifications with more detailed subgroups (e.g., receptor tyrosine kinase, non-receptor tyrosine kinase, serine/threonine kinase, epigenetic, Bcl-2, hedgehog pathway, PARP, and proteasome SMIs^35^ could yield different results for this subgroup.

This study focused on PDACs, as they are the most common type of pancreatic cancer. However, other tumor types, such as neuroendocrine and exocrine tumors, are also found in human patients and were not addressed in this study^1,4^. The transgenic models included in the systematic review predominantly featured tumors other than PDACs. These models were excluded from the meta-analysis due to the spontaneous development of tumors, often with multiple nodules, making it difficult to obtain data comparable to injected single tumors. Further research in this area could be valuable for improving the reproducibility of animal studies and facilitating the translation of preclinical research to clinical trials.

The human pancreas, located in the abdominal cavity behind the stomach, consists of four main sections: head, neck, body, and tail, with the head positioned toward the duodenum^1,4,36,37^. Most PDACs arise in the pancreatic head^6^, yet the majority of individual studies conducting orthotopic tumor establishment implanted cells or patient-derived tissue in the tail region. This is likely due to easier accessibility of the tail compared to the head. However, not all studies performing intrapancreatic implantation reported the specific pancreatic region, which may introduce bias into the evidence. However, this discrepancy should be considered when translating findings to human disease.

The National Library of Medicine (NIH) currently lists 19 clinical trials related to PC and SMI therapy, including trials that are not yet recruiting, recruiting, active, or completed. Of these, ten involve combination therapies with other SMIs or chemotherapeutics, seven focus on single SMI therapies, and two have been withdrawn^38^. The approved drugs for PC include seven different chemotherapeutics and three SMIs (Erlotinib, Olaparib, and Sunitinib). Erlotinib is approved only in combination with Gemcitabine for non-surgical, metastatic tumors, Olaparib is approved for non-surgical, metastatic NETs, and Sunitinib is used as maintenance therapy for non-progressive, metastatic tumors^39^. In this study, we focused on single SMI therapies and initially excluded combination therapies. However, given the prevalence of clinical trials involving combination therapies and the availability of approved combination therapies, this could be a promising area for future research. Additionally, it is noticeable that therapies have only been approved for metastatic tumors as patients are usually diagnosed with PC only after the disease has already metastasized^1,5,6^. However, we found that preclinical studies on SMI therapy with primary tumor models are dominating over metastatic models. The number of individual studies we identified were even insufficient for meta-analysis, raising the question of why research focus is not directed towards the clinical context.

In conclusion, our results demonstrated significant reductions in pancreatic tumor volume, weight, and area following treatment with SMIs. However, this systematic review and meta-analysis highlighted the substantial variability among preclinical PC mouse models used to investigate SMIs. We identified various factors that may contribute to the translational discrepancies between preclinical studies and clinical trials, while also highlighting that incomplete reporting limits our ability to comprehensively evaluate the reproducibility and validity of these animal models. There is a critical need for enhanced methodological reporting in accordance with the ARRIVE guidelines. Future systematic reviews and meta-analyses across different cancer entities and therapeutic approaches could offer new insights, enabling researchers to design studies with more suitable models tailored to specific research questions. This would help reduce the translational gap and improve the reproducibility and reliability of preclinical findings.

## Materials and methods

This systematic review was conducted according to a preregistered protocol in PROSPERO (CRD42022314932; available from: crd.york.ac.uk/prospero/display_record.php? RecordID = 314932) to address the research question ‘Which pancreatic mouse cancer model is suitable to investigate the effects of small molecule inhibitors for the treatment of primary tumors and metastasis compared to untreated controls?’^40^. The question was structured using the PICO format (Population, Intervention, Comparison, Outcome) with P as pancreatic mouse cancer model, I as small molecule inhibitors, C as untreated controls, O as effects on primary tumors and metastasis. Originally, we intended in the protocol a risk of bias analysis by use of SYRCLE’s tool and no quality assessment. However, we reached a decision to preferably assess the reporting quality based on the ARRIVE Essential 10 guidelines 2.0, as the experimental design of small animal studies is usually fraught with problems of randomization and blinding and thus with a high risk of bias^15,33,41^. With the ARRIVE Essential 10 guidelines 2.0, the adequate reporting of animal studies with their methodological details is ensured which is crucial for transparency, reproducibility, and identification of potential biases in research through improved reporting standards^15^. We focused in this study on the primary outcome data for tumor volumes, weights and area while secondary outcomes were not analyzed. Apart from this modification, we adhered to the original protocol. We investigated sex, transplantation method, tumor graft method, route of application of tumor cells or tissues, genetic background of mice, injected tumor cell or tissue type, small molecule inhibitory category, and route of drug application, randomization and blinding as potential sources of heterogeneity.

### Search strategy

The systematic bibliography research was conducted in two different databases, Pubmed (MEDLINE) and Embase (Elsevier), in January 2025 by one author (SV). All original available full-text studies up to this time point were included in this review. The search strategy was based on the research question, using the following keywords in titles and abstracts: “small molecules”, “small molecule inhibitors”, “small molecule therapy”, “inhibition”, “growth inhibition”, “growth suppression”, “in vivo”, “xenograft model antitumor assays”, “animal experiment”, “animal model”, “in vivo study”, “mouse model”, and “murine model”. The Medical Subject Heading (MeSH) term “pancreatic neoplasms” was added to the search strategy to include all possible terms related to pancreatic neoplasms, such as “pancreatic cancer” and “pancreas cancer”, to ensure that no publications on this topic were missed. To only include animal studies, a filter for animals was applied. Review articles were excluded from the search. The search strategy was further refined using the operators “AND”, “OR” and “NOT”. The detailed search strategy is available in the supplemental information (SI) (see supplemental search strategy). The bibliographies obtained from Pubmed (MEDLINE) and Embase (Elsevier) were imported into the reference management software Mendeley (RRID: SCR_002750) and subsequently separately into the online tool Rayyan QCRI (RRID: SCR_017584). Using this tool, duplicates were removed at the outset (SV) and the study decision process began with an initial blinded screening of titles and abstracts by two independent reviewers (SV, YM).

### Inclusion and exclusion criteria

Original controlled mouse studies at any sex and any age published in English using a single small molecule inhibitor (molecular size ranging from 0.1 to 1.0 kDa) to treat metastatic and non-metastatic pancreatic cancer were included. Studies involving combinational therapies with more than one small molecule inhibitor (SMI) or with other therapeutic regimens (e.g. chemotherapy, radiotherapy, immunotherapy) were excluded. There were no restrictions on the mode of intervention application. Orthotopic and subcutaneous, as well as syngeneic, allogeneic and xenogeneic (cell line-generated, patient-derived) mouse models were included. Genetically modified mouse models were also considered. Irrelevant studies were excluded based on predefined criteria consisting of opinion articles, observational studies, abstracts without adequate data, systematic reviews, letters, editorials, reviews, case reports, clinical studies, in vitro studies. Excluded populations were humans, wild-life and pet animals. The screening process was conducted in two steps and articles were included if they met the predefined inclusion criteria. First, titles and abstracts were independently screened by two reviewers (SV, YM). Second, full-text screening of potentially eligible articles was performed by one reviewer (SV). Any queries were discussed with a second reviewer (YM) and disagreements were resolved through discussion or by consultation of a third reviewer (JS).

### Data extraction

One reviewer (SV) assessed each included article to extract bibliographic data (first author, year of publication, journal, ethical approval), animal model characteristics (sex, age, body weight, strain, number), study design data (randomization, blinding, method of pancreatic cancer induction, tumor size at the beginning time of intervention, starting time of study, study duration), intervention characteristics (therapeutic and control agents, dosage regimen, route of application) and primary outcomes (tumor weight, tumor volume, tumor area). The data were recorded into a purpose-built Microsoft Excel spreadsheet (RRID: SCR_016137). We initially attempted to extract numerical data from tables, or text. In studies where numerical data were not reported and only presented graphically, a screenshot of the graphical data was taken, and an adequate estimation of the outcome measurements was extracted from graphs using WebPlotDigitizer (RRID: SCR_013996). In studies where data were unobtainable or unclear, an initial request for unpublished information was sent to the corresponding author via email. If there was no response to the initial email after a minimum of seven business days, a second reminder email was sent. If there was still no response after a minimum of ten business days following the reminder, or if the email was undeliverable and data remained missing, the results were excluded from the analysis. In cases where the primary outcome was measured repeatedly at different time points, only data from the endpoint (the last day of the experiment) were extracted. If the control group was terminated earlier than the intervention group, the primary outcome data from the control group’s endpoint were extracted. When more than one experiment was reported in the same manuscript with separate animals used for treatment groups, the experiments were included as separate comparisons in the analysis. If one control group was used for multiple intervention groups, the number of animals in the control group was divided by the number of intervention groups for meta-analysis. Extracted data were discussed in cases of uncertainty regarding eligibility, and final decisions were made in a meeting with a second and third reviewer (YM, JS).

### Reporting quality assessment

The quality of reporting was assessed for each of the included article (*n* = 297) based on the ARRIVE (*A*nimal *R*esearch: *R*eporting of *In* *Vivo* *E*xperiments) Essential 10 guidelines 2.0^15^. The following ten items were evaluated, as they represent key elements that should be included in any manuscript describing animal studies: study design, sample size, inclusion and exclusion criteria, animal randomization, blinding, outcome measures, statistical methods, experimental animals, experimental procedures, and results. Each item was scored separately for the reporting quality. If all criteria for an item were addressed, it was scored as fully reported (2 ≡ low concerns). If only some criteria were addressed, it was scored as partially reported (1 ≡ some concerns) and if none of the criteria were addressed, it was scored as not reported (0 ≡ high concerns). The data were collected in Microsoft Excel spreadsheet (RRID: SCR_016137). The reporting quality was assessed using the initial information extracted for the systematic review prior to contacting authors for additional information for meta-analysis. The results of the reporting quality assessment were visualized in a summary plot using the ROB2 template in the risk-of-bias tool^18^.

### Data synthesis and statistical analysis

We conducted meta-analyses using the extracted primary outcome data from the included studies on primary tumor volume, tumor weight and tumor area. Meta-analyses were performed using R V.4.2.3 (RRID: SCR_009175) when at least 4 individual studies per group (tumor volume, tumor weight, tumor area) were available. In all comparisons, data were reported as a mean outcome score with a measure of variance. Therefore, we calculated either a normalized difference in means (NMD) or a standardized difference in means (SMD). For primary tumor volume and tumor weight, we presented the normalized mean difference as data were on a ratio scale and sham data could be inferred^42^. This approach describes the direction and magnitude of the treatment effect, with effect sizes typically falling between − 100% and + 100%. For primary tumor area, we presented continuous data with the SMD with a 95% confidence interval (CI). When variance was provided as standard error of the mean, the standard deviation (SD) was calculated using the following equations (where *n* is the number of mice used for the value): $$\:\text{S}\text{D}=\text{S}\text{E}\text{M}\times\:\sqrt{n}$$ or $$\:\text{S}\text{E}\text{M}=\raisebox{1ex}{$\text{S}\text{D}$}\!\left/\:\!\raisebox{-1ex}{$\sqrt{n}$}\right.$$. A random-effects model was selected to account for weighting and variation among studies. By calculating the 95% prediction interval (PI), we identified an estimated range within which the true effect of a new study from the population of studies would fall in 95% of cases. Variance estimates were calculated using restricted maximum likelihood (REML) to provide unbiased estimates of variance and covariance parameters. The degree of heterogeneity was quantified using the I^2^ statistic index. We considered I^2^ values between 50% and 70% as moderate heterogeneity, and I^2^ > 70% as considerable heterogeneity. To further explore potential sources of heterogeneity, we performed uni- and multivariable meta-regression. Variables included sex, transplantation method, tumor graft method, route of application of tumor cells or tissues, genetic background of mice, injected tumor cell or tissue type, small molecule inhibitory category, and route of drug application, baseline tumor size, and risk-of-bias factors (randomization and blinding). Heterogeneity was described using the following metrics, Q (heterogeneity statistic), tau^2^ (estimation of between-study variance), residual I^2^ (percentage of residual variation attributable to between-study heterogeneity), and adjusted R^2^ (proportion of between-study variance explained by the covariate). Plots were created using the orchaRd 2.0 R package^42^. To assess asymmetry arising from small study effects and potential publication bias, we visually inspected the funnel plots and applied Egger’s regression test (a weighted linear regression of the treatment effect on its standard error) were applicable. The trim-and-fill method was further used to adjust pooled effect estimate for potential funnel plot asymmetry when appropriate^18,43^.

## Supplementary Information

Below is the link to the electronic supplementary material.

Supplementary Material 1

## Data Availability

Detailed information from data extraction and the analytic code for this study are available on Open Science Framework at https://osf.io/eht5k/?view_only=b3dd918771114180ada099d78752e26e.

## References

[1] Aslan, M., Shahbazi, R., Ulubayram, K. & Ozpolat, B. Targeted therapies for pancreatic cancer and hurdles ahead. *Anticancer Res.* **38**, 6591–6606. 10.21873/anticanres.13026 (2018). 30504367 10.21873/anticanres.13026

[2] Huang, J. et al. Worldwide burden of, risk factors for, and trends in pancreatic cancer. *Gastroenterology* **160**, 744–754 (2021). 33058868 10.1053/j.gastro.2020.10.007

[3] American Cancer Society. Cancer Facts & Figs. (2022).

[4] Aier, I., Semwal, R., Sharma, A. & Varadwaj, P. K. A systematic assessment of statistics, risk factors, and underlying features involved in pancreatic cancer. *Cancer Epidemiol.* **58**, 104–110 (2019). 10.1016/j.canep.2018.12.001 Preprint at. 30537645

[5] Rawla, P., Sunkara, T. & Gaduputi, V. Epidemiology of pancreatic cancer: global Trends, etiology and risk factors. *World J. Oncol.* **10**, 10–27 (2019). 30834048 10.14740/wjon1166 PMC6396775

[6] McGuigan, A. et al. Pancreatic cancer: A review of clinical diagnosis, epidemiology, treatment and outcomes. *World J. Gastroenterol.* **24**, 4846–4861 (2018). 30487695 10.3748/wjg.v24.i43.4846 PMC6250924

[7] Amjad, M. T., Chidharla, A. & Kasi, A. *Cancer Chemotherapy* (StatPearls Publishing LLC, 2022). 33232037

[8] Lavanya, V., Adil, M., Ahmed, N., Rishi, A. K. & Jamal, S. Integrative cancer science and therapeutics small molecule inhibitors as emerging cancer therapeutics. *Integr. Cancer Sci. Th.*10.15761/ICST.1000109 (2014).

[9] Khera, N. & Rajput, S. Therapeutic potential of small molecule inhibitors. *J. Cell. Biochem.* **118**, 959–961 (2017). 27813176 10.1002/jcb.25782

[10] Liu, G., Chen, T., Zhang, X., Ma, X. & Shi, H. Small molecule inhibitors targeting the cancers. *MedComm (Beijing).* **3** (2022). 10.1002/mco2.181 PMC9560750 36254250

[11] Garcia-Sampedro, A., Gaggia, G., Ney, A., Mahamed, I. & Acedo, P. The State-of-the-Art of phase II/III clinical trials for targeted pancreatic cancer therapies. *J. Clin. Med.* **10**, 566 (2021). 33546207 10.3390/jcm10040566 PMC7913382

[12] Day, C. P., Merlino, G. & Van Dyke, T. Preclinical Mouse Cancer Models: A Maze of Opportunities and Challenges. *Cell* **163**, 39–53. 10.1016/j.cell.2015.08.068 (2015). 26406370 10.1016/j.cell.2015.08.068 PMC4583714

[13] Guerin, M. V., Finisguerra, V., Van den Eynde, B. J., Bercovici, N. & Trautmann, A. Preclinical murine tumor models: A structural and functional perspective.. *Elife*10.7554/eLife.50740 (2020). 31990272 10.7554/eLife.50740 PMC6986875

[14] Saloman, J. L. et al. nimal Models: Challenges and Opportunities to Determine Optimal Experimental Models of Pancreatitis and Pancreatic Cancer. *Pancreas* **48**, 759–779. 10.1097/MPA.0000000000001335 (2019). 31206467 10.1097/MPA.0000000000001335 PMC6581211

[15] du Sert, N. et al. (ed, P.) Reporting animal research: explanation and elaboration for the arrive guidelines 2.0. *PLoS Biol.* **18** Preprintathttpsdoiorg101371journalpbio3000411 (2020). 10.1371/journal.pbio.3000411 PMC7360025 32663221

[16] Page, M. J. et al. The PRISMA 2020 statement: an updated guideline for reporting systematic reviews. *BMJ n71*10.1136/bmj.n71 (2021). 10.1136/bmj.n71 PMC8005924 33782057

[17] Kilkenny, C., Browne, W., Cuthill, I. C., Emerson, M. & Altman, D. G. Animal research: Reporting in vivo experiments: The ARRIVE guidelines. *Br J Pharmacol* **160**, 1577–1579. 10.1111/j.1476-5381.2010.00872.x (2010). 20649561 10.1111/j.1476-5381.2010.00872.x PMC2936830

[18] McGuinness, L. A. & Higgins, J. P. T. Risk-of-bias VISualization (robvis): An R package and Shiny web app for visualizing risk-of-bias assessments. *Res Synth Methods.* (2020). 10.1002/jrsm.1411 32336025

[19] Camarades Berlin & Quest-Bih Charité. Preclinical systematic reviews & Meta-Analysis Wiki. https://www.CAMARADES.de (2024).

[20] Deer, E. L. et al. Phenotype and genotype of pancreatic cancer cell lines. *Pancreas* **39**, 425–435 (2010). 20418756 10.1097/MPA.0b013e3181c15963 PMC2860631

[21] Murtaugh, L. C. Pathogenesis of pancreatic cancer. *Toxicol. Pathol.* **42**, 217–228 (2014). 24178582 10.1177/0192623313508250 PMC3926968

[22] Liu, M. & Yang, X. Patient-derived xenograft models: current status, challenges, and innovations in cancer research. *Genes Dis.* **12**, 101520 (2025). 40548062 10.1016/j.gendis.2025.101520 PMC12179623

[23] Liu, Y. et al. Patient-derived xenograft models in cancer therapy: technologies and applications. *Signal. Transduct. Target. Ther.* **8**, 160 (2023). 37045827 10.1038/s41392-023-01419-2 PMC10097874

[24] Patino, C. M. & Ferreira, J. C. Internal and external validity: can you apply research study results to your patients? *Jornal Brasileiro De Pneumologia*. **44**, 183 (2018). 30043882 10.1590/S1806-37562018000000164 PMC6188693

[25] Russell, A. A. M., Sutherland, B. A., Landowski, L. M., Macleod, M. & Howells, D. W. What has preclinical systematic review ever done for us?. *BMJ Open Sci.*10.1136/bmjos-2021-100219 (2022). 35360370 10.1136/bmjos-2021-100219 PMC8921935

[26] van Luijk, J. et al. Systematic reviews of animal Studies; missing link in translational research? *PLoS One*. **9**, e89981 (2014). 24670965 10.1371/journal.pone.0089981 PMC3966727

[27] Soliman, N., Rice, A. S. C. & Vollert, J. A practical guide to preclinical systematic review and meta-analysis. *Pain* **161**, 1949–1954 (2020). 33449500 10.1097/j.pain.0000000000001974 PMC7431149

[28] Lin, Y. et al. Reporting quality of animal research in journals that published the ARRIVE 1.0 or ARRIVE 2.0 guidelines: a cross-sectional analysis of 943 studies. *Cardiovasc. Diagn. Ther.* **14**, 1070–1082 (2024). 39790189 10.21037/cdt-24-413 PMC11707473

[29] Sena, E. S., Currie, G. L., McCann, S. K., Macleod, M. R. & Howells, D. W. Systematic reviews and Meta-Analysis of preclinical studies: why perform them and how to appraise them critically. *J. Cereb. Blood Flow. Metabolism*. **34**, 737–742 (2014). 10.1038/jcbfm.2014.28 PMC4013765 24549183

[30] Tenorio-Pedraza, J. M., Lippert, J., Burghaus, R. & Scheerans, C. R. E. S. E. A. R. C. H. Open access Meta-analysis of preclinical measures of efficacy in immune checkpoint Blockade therapies and comparison to clinical efficacy estimates. *Transl Med. Commun.* **8**, 17 (2023).

[31] Sterne, J. A. C. et al. Recommendations for examining and interpreting funnel plot asymmetry in meta-analyses of randomised controlled trials. *BMJ* **343**, d4002–d4002 (2011). 21784880 10.1136/bmj.d4002

[32] van der Worp, H. B. et al. Can animal models of disease reliably inform human studies? *PLoS Med.* **7**, e1000245 (2010). 20361020 10.1371/journal.pmed.1000245 PMC2846855

[33] Sena, E. S., van der Worp, H. B., Bath, P. M. W., Howells, D. W. & Macleod, M. R. Publication bias in reports of animal stroke studies leads to major overstatement of efficacy. *PLoS Biol.* **8**, e1000344 (2010). 20361022 10.1371/journal.pbio.1000344 PMC2846857

[34] de Vries, R. B. M. et al. The usefulness of systematic reviews of animal experiments for the design of preclinical and clinical studies. *ILAR J.* **55**, 427–437 (2014). 25541545 10.1093/ilar/ilu043 PMC4276599

[35] Zhong, L. et al. Small molecules in targeted cancer therapy: advances, challenges, and future perspectives. *Signal. Transduct. Target. Ther.* **6**, 201 (2021). 34054126 10.1038/s41392-021-00572-w PMC8165101

[36] Pansky, B. Anatomy of the pancreas. *Int. J. Pancreatol.* **7**, 101–108 (1990). 2081913

[37] Mahadevan, V. Anatomy of the pancreas and spleen. *Surg. (Oxford)*. **37**, 297–301 (2019).

[38] National Library of Medicine NIH. About ClinicalTrials.gov. *https://clinicaltrials.gov/* at < https://clinicaltrials.gov/search?cond=Pancreatic%20Cancer&term=Pancreas%20Cancer&intr=small%20molecule> (2024).

[39] National Cancer Institute NIH. Drugs Approved for Pancreatic Cancer. https://www.cancer.gov/about-cancer/treatment/drugs/pancreatic at https://www.cancer.gov/about-cancer/treatment/drugs/olaparib (2024).

[40] Villwock, S., Mirzaei, Y., Dahl, E. & Steitz, J. Experimental pancreatic cancer mouse models for treatment with small molecule inhibitors: A systematic review and meta-analysis. https://www.crd.york.ac.uk/prospero/display_record.php?RecordID=314932 (2022).10.1038/s41598-025-25191-141145806

[41] Hooijmans, C. R. et al. SYRCLE’s risk of bias tool for animal studies. *BMC Med. Res. Methodol*10.1186/1471-2288-14-43 (2014). 24667063 10.1186/1471-2288-14-43 PMC4230647

[42] Cohen, J. A coefficient of agreement for nominal scales. *Educ. Psychol. Meas.* **20**, 37–46 (1960).

[43] Landis, J. R. & Koch, G. G. The measurement of observer agreement for categorical data. *Biometrics* **33**, 159 (1977). 843571

