# Supplementary material for: Preclinical pancreatic cancer mouse models for treatment with small molecule inhibitors: a systematic review and meta-analysis
Source: Sci Rep. 2025 Oct 27;15:37358. doi: 10.1038/s41598-025-25191-1 (PMC12559716; doi:10.1038/s41598-025-25191-1)
Supplement: Supplementary file 1 — Supplementary Material 1 [file 41598_2025_25191_MOESM1_ESM.docx]

**SUPPLEMENTAL INFORMATION (SI)**

Preclinical pancreatic cancer mouse models for treatment with small molecule inhibitors: A systematic review and meta-analysis

Sophia Villwock^1^, Yalda Mirzaei^1^, Edgar Dahl^2^, Julia Steitz^1^

^1^Institute for Laboratory Animal Science, Medical Faculty, RWTH Aachen University, Aachen, Germany

^2^Institute of Pathology, Medical Faculty, RWTH Aachen University, Aachen, Germany

^3^Center for Integrated Oncology Aachen Bonn Cologne Düsseldorf (CIO-ABCD), Aachen, Germany

*Authors contribute equally

Corresponding author:

Julia Steitz

Institute for Laboratory Animal Science

Medical Faculty, RWTH Aachen University

Pauwelsstrasse 30

52074 Aachen

Phone: +49 (0)241 80-88603

Email: [jsteitz@ukaachen.de](mailto:jsteitz@ukaachen.de)

**Supplemental search strategy:**

Pubmed MEDLINE

(((((((((((pancreatic neoplasms[Title/Abstract])) OR (pancreatic neoplasm[Title/Abstract])) OR (pancreatic cancer[Title/Abstract])) OR (pancreas cancer[Title/Abstract])) OR (pancreas neoplasms[Title/Abstract])) OR (pancreas neoplasm[Title/Abstract])) AND (small molecule therapy[Title/Abstract])) OR (small molecules[Title/Abstract])) OR (small molecule inhibitor[Title/Abstract])) OR (small molecule inhibitors[Title/Abstract])) OR (inhibition[Title/Abstract]) OR (growth inhibition[Title/Abstract]) OR (growth suppression[Title/Abstract]) AND (in vivo[Title/Abstract]) OR (Xenograft Model Antitumor Assays[Title/Abstract]) NOT (review[Title/Abstract]) AND (pancreas neoplasm[MeSH Terms]) AND (animal[Filter]) Filters: Other Animals

((((((("pancreatic neoplasms"[Title/Abstract] OR "pancreatic neoplasm"[Title/Abstract] OR "pancreatic cancer"[Title/Abstract] OR "pancreas cancer"[Title/Abstract] OR "pancreas neoplasms"[Title/Abstract] OR "pancreas neoplasm"[Title/Abstract]) AND "small molecule therapy"[Title/Abstract]) OR "small molecules"[Title/Abstract] OR "small molecule inhibitor"[Title/Abstract] OR "small molecule inhibitors"[Title/Abstract] OR "inhibition"[Title/Abstract] OR "growth inhibition"[Title/Abstract] OR "growth suppression"[Title/Abstract]) AND "in vivo"[Title/Abstract]) OR "xenograft model antitumor assays"[Title/Abstract]) NOT "review"[Title/Abstract]) AND "pancreatic neoplasms"[MeSH Terms] AND "animals"[MeSH Terms:noexp]) AND (animal[Filter])

Translations:

pancreas neoplasm[MeSH Terms]: "pancreatic neoplasms"[MeSH Terms]

animal[Filter]: animals[mh:noexp]

Embase Elsevier

#1

((('pancreatic neoplasms':ti,ab OR 'pancreatic neoplasm':ti,ab OR 'pancreatic cancer':ti,ab OR 'pancreas cancer':ti,ab OR 'pancreas neoplasms':ti,ab OR 'pancreas neoplasm':ti,ab) AND 'small molecule therapy':ti,ab OR 'small molecules':ti,ab OR 'small molecule inhibitor':ti,ab OR 'small molecule inhibitors':ti,ab OR inhibition:ti,ab OR 'growth inhibition':ti,ab OR 'growth suppression':ti,ab) AND 'in vivo':ti,ab OR 'xenograft model antitumor assays':ti,ab) NOT review:ti,ab AND ('pancreas neoplasm'/exp OR 'pancreas neoplasm')

#2

#1 AND ('animal experiment'/de OR 'animal model'/de OR 'in vivo study'/de OR 'mouse model'/de OR 'murine model'/de)

**Supplemental Tables:**

Detailed information on characteristics from data extraction and the analytical code are available on the open science framework at https://osf.io/eht5k/?view_only=b3dd918771114180ada099d78752e26e.

Table 1. Main bibliographic data of the 297 articles included in the systematic review.

| Reference | First author | Journal | Year |
| --- | --- | --- | --- |
| [1] | Emmanuel E. Zervos | Journal of Surgical Research | 1997 |
| [2] | Akiko Saito | Proceedings of the National Academy of Sciences of the USA | 1999 |
| [3] | Grégoire P. Prevost | International Journal of Cancer | 1999 |
| [4] | Ramon E. Jimenez | Annals of Surgery | 2000 |
| [5] | Mahmudul Haq | Cancer Research | 2000 |
| [6] | Grégoire P. Prevost | International Journal of Cancer | 2001 |
| [7] | David W. End | Cancer Research | 2001 |
| [8] | Robert B. Lobell | Cancer Research | 2001 |
| [9] | Wie-Gang Tong | Clinical Cancer Research | 2002 |
| [10] | Hubert G.Hotz | Journal of Gastrointestinal Surgery | 2003 |
| [11] | Rosa F. Hwang | Clinical Cancer Research | 2003 |
| [12] | Susann Stephan | Clinical Cancer Research | 2004 |
| [13] | Maksim V. Yezhelyev | Clinical Cancer Research | 2004 |
| [14] | Kiranmai Gumireddy | Cancer Cell | 2005 |
| [15] | Hiromi Kazuno | Cancer Science | 2005 |
| [16] | Marie-Christine Brezak | Molecular Cancer Therapeutics | 2005 |
| [17] | Cindy Y. F. Yau | Cancer Research | 2005 |
| [18] | Donna L. Dehn | Molecular Cancer Therapeutics | 2006 |
| [19] | Alan J. Durkin | Journal of Surgical Research | 2006 |
| [20] | Cataldo Bianco | Clinical Cancer Research | 2006 |
| [21] | Maria S. Pino | Cancer Research | 2006 |
| [22] | Antonio Jimeno | Molecular Cancer Therapeutics | 2007 |
| [23] | Collins A. Karikari | Molecular Cancer Therapeutics | 2007 |
| [24] | Nicholas J. Gaspar | Molecular Pharmacology | 2007 |
| [25] | Soichi Kobayashi | Cancer Science | 2007 |
| [26] | Ivan Ischenko | Angiogenesis | 2007 |
| [27] | Peter Büchler | Neoplasia | 2007 |
| [28] | Jamael El Fitroi | Cancer | 2007 |
| [29] | Satyanarayana Medicherla | Anticancer Research | 2007 |
| [30] | Daisuke Kawasaki | Cancer Chemotherapy and Pharmacology | 2007 |
| [31] | Belen Rubio-Viqueria | Molecular Cancer Therapeutics | 2007 |
| [32] | A. Scholz | Gut | 2008 |
| [33] | Georg Feldmann | Molecular Cancer Therapeutics | 2008 |
| [34] | Matthias Kapischke | International Journal of Oncology | 2008 |
| [35] | Walter Gregory Roberts | Cancer Research | 2008 |
| [36] | Martin Haefner | World Journal of Gastroenterology | 2008 |
| [37] | Christian Moser | European Journal of Cancer | 2008 |
| [38] | Ying-Ying Lu | World Journal of Gastroenterology | 2008 |
| [39] | Dongweon Song | Molecular Cancer Therapeutics | 2008 |
| [40] | Atsushi Nakajima | Cancer Science | 2008 |
| [41] | Sven A. Lang | Molecular Cancer Therapeutics | 2008 |
| [42] | Asfar Sohail Azmi | Molecular Cancer Therapeutics | 2008 |
| [43] | Callum M. Sloss | Clinical Cancer Research | 2008 |
| [44] | Xianhua Cao | Clinical Cancer Research | 2008 |
| [45] | Takanori Ochiai | Journal of Research in Medical and Dental Science | 2008 |
| [46] | Yixian Zhang | Cancer Research | 2008 |
| [47] | Anthony L. Schwartz | Clinical Cancer Research | 2009 |
| [48] | Jie Lu | Molecular Cancer Therapeutics | 2009 |
| [49] | Haiyan Lu | Clinical Cancer Research | 2009 |
| [50] | Qing Chang | Cancer Biology & Therapy | 2009 |
| [51] | Chao Yan | Molecular Pharmacology | 2009 |
| [52] | Masahiro Komoto | Cancer Science | 2009 |
| [53] | Steven N. Hochwald | Cell Cycle | 2009 |
| [54] | John McLaughlin | Journal of Cancer Research and Clinical Oncology | 2010 |
| [55] | Koh Furugaki | Oncology Letters | 2010 |
| [56] | Nagaraj S. Nagathihalli | Molecular Cancer Therapeutics | 2010 |
| [57] | Holly K. Koblish | Molecular Cancer Therapeutics | 2010 |
| [58] | Soumya Jaganathan | The Journal of Pharmacology and Experimental Therapeutics | 2010 |
| [59] | Yu-Wen Zhang | Cancer Research | 2010 |
| [60] | Kuzhuvelil B. Harikumar | Molecular Cancer Therapeutics | 2010 |
| [61] | Donghang Zheng | Molecular Carcinogenesis | 2010 |
| [62] | Xiong Cai | Journal of Medicinal Chemistry | 2010 |
| [63] | Georg Feldmann | Cancer Biology & Therapy | 2011 |
| [64] | Volker Fendrich | Annals of Surgery | 2011 |
| [65] | Martin F. Strand | PLOS ONE | 2011 |
| [66] | Johannes Taeger | Molecular Cancer Therapeutics | 2011 |
| Reference | First author | Journal | Year |
| [67] | Nagaraj S. Nagathihalli | Clinical Cancer Research | 2011 |
| [68] | Olga K. Mirzoeva | Journal of Molecular Medicine | 2011 |
| [69] | Poitr Jasinski | Anticancer Research | 2011 |
| [70] | Vladimir Beljanski | Investigational New Drugs | 2011 |
| [71] | Toshio Fujisawa | Journal of Translational Meicine | 2011 |
| [72] | Niranjan Awasthi | Molecular Cancer Therapeutics | 2012 |
| [73] | Dmitriy I. Dovzhanskiy | BMC Cancer | 2012 |
| [74] | Rosa F. Hwang | Molecular Cancer Research | 2012 |
| [75] | Katharina Fraedrich | Clinical Cancer Research | 2012 |
| [76] | Keith B. Glaser | The Journal of Pharmacology and Experimental Therapeutics | 2012 |
| [77] | Christian Moser | Anticancer Research | 2012 |
| [78] | Mayumi Komachi | Cancer Science | 2012 |
| [79] | Volker Fendrich | The Scientific World Journal | 2012 |
| [80] | Irmgard Hofmann | PLOS ONE | 2012 |
| [81] | Niranjan Awasthi | PLOS ONE | 2012 |
| [82] | Christian Moser | Anticancer Research | 2012 |
| [83] | Friedhelm Bladt | Clinical Cancer Research | 2013 |
| [84] | Evan G. Gomes | Pancreas | 2013 |
| [85] | Shinichi Yabuuchi | Cancer Letters | 2013 |
| [86] | Fardokht Abulwerdi | Molecular Cancer Therapeutics | 2013 |
| [87] | Elena Kurenova | Oncotarget | 2013 |
| [88] | Dao-Jun Gong | Clinical Interventions in Aging | 2013 |
| [89] | Deniz A. Ucar | Anti-Cancer Agents in Medicinal Chemistry | 2013 |
| [90] | Gerardo G. Mackenzie | Neoplasia | 2013 |
| [91] | Hua Zhong | PLOS ONE | 2013 |
| [92] | Gunther Zimmermann | Nature | 2013 |
| [93] | Vindhya Palagani | Carcinogenesis | 2014 |
| [94] | Marisa O. Peluso | PLOS ONE | 2014 |
| [95] | Volker Fendrich | Neuroendocrinology | 2014 |
| [96] | Ethan V. Abel | PLOS ONE | 2014 |
| [97] | Lucero-Acuña | International Journal of Nanomedicine | 2014 |
| [98] | Thomas J. Hayman | Clinical Cancer Research | 2014 |
| [99] | H. Zhang | British Journal of Cancer | 2014 |
| [100] | Wenwen Chien | Oncotarget | 2014 |
| [101] | Joseph D. Valentino | Clinical Cancer Research | 2014 |
| [102] | Matthew H. Wong | Clinical Cancer Research | 2014 |
| [103] | Xueli Bai | Cancer Letters | 2014 |
| [104] | Andreas M. Heilmann | Cancer Research | 2014 |
| [105] | Wenwen Chien | Molecular Carcinogenesis | 2014 |
| [106] | Priyanka N. Gogate | Cancer Letters | 2014 |
| [107] | Yi Zhong | Clinical Cancer Research | 2014 |
| [108] | Muayad Almahariq | Molecular Pharmacology | 2014 |
| [109] | Jumben Wu | Current Signal Transduction Therapy | 2014 |
| [110] | Susann Weissmüller | Cell | 2014 |
| [111] | Garth Hamilton | Oncotarget | 2014 |
| [112] | Xiaobing Deng | Genes & Cancer | 2014 |
| [113] | Thomas A. Mace | Oncotarget | 2015 |
| [114] | Xiangxuan Zhao | Molecular Cancer Therapeutics | 2015 |
| [115] | Sabiha Kazim | Molecular Cancer Therapeutics | 2015 |
| [116] | Shang Minjie | Tumor Biology | 2015 |
| [117] | Yoko Hari | Oncotarget | 2015 |
| [118] | Hironobu Takano | Oncotarget | 2015 |
| [119] | Franziska Brandes | BMC Cancer | 2015 |
| [120] | Xiaohua Gao | International Journal of Oncology | 2015 |
| [121] | Niranjan Awasthi | Cancer Letters | 2015 |
| [122] | Feng Wei | International Journal of Molecular Sciences | 2015 |
| [123] | Yan Li | Cancer Letters | 2015 |
| [124] | Nagaraj S. Nagathihalli | Gastroenterology | 2015 |
| [125] | Francesca Vena | Clinical Cancer Research | 2015 |
| [126] | Rony A. François | Journal of the National Cancer Institute | 2015 |
| [127] | Ingrid Moen | Clinical & Experimental Metastasis | 2015 |
| [128] | Niranjan Awasthi | Oncotarget | 2016 |
| [129] | Keisuke Yamamoto | Oncotarget | 2016 |
| [130] | Alejandro Recio-Boiles | Oncotarget | 2016 |
| [131] | Yongsheng Jiang | Biochemical and Biophysical Research Communications | 2016 |
| [132] | Dannel Yeo | BMC Cancer | 2016 |
| [133] | Carolyn C. Arpin | Molecular Cancer Therapeutics | 2016 |
| [134] | Darlene Barnard | Invest New Drugs | 2016 |
| [135] | Sally E. Henderson | Neoplasia | 2016 |
| [136] | Hee Seung Lee | Scientific Reports | 2017 |
| [137] | Jair Machado Espindola-Netto | Oncotarget | 2017 |
| [138] | Casey G. Langdon | Molecular Cancer Therapeutics | 2017 |
| Reference | First author | Journal | Year |
| [139] | Hua Li | Neoplasia | 2017 |
| [140] | Angela Mathison | Molecular Cancer Research | 2017 |
| [141] | Jingkai Zhou | Oncotarget | 2017 |
| [142] | Yi-Jin Chen | PLOS ONE | 2017 |
| [143] | Kai Wang | International Journal of Oncology | 2017 |
| [144] | Daoxiang Zhang | Clinical Cancer Research | 2017 |
| [145] | Kanjoormana Aryan Manu | Molecular Cancer Therapeutics | 2017 |
| [146] | Wen-Hua Chen | Acta Pharmacologica Sinica | 2017 |
| [147] | Soichi Takiguchi | International Journal of Oncology | 2017 |
| [148] | Forest H. Andrews | Proceedings of the National Academy of Sciences of the USA | 2017 |
| [149] | Wenhua Chen | European Journal of Medicinal Chemistry | 2017 |
| [150] | Nicola Rath | Cancer Research | 2018 |
| [151] | C. S. Lewis | Journal of Thrombosis and Haemostasis | 2018 |
| [152] | Encheng Bai | Cancer Management and Research | 2018 |
| [153] | Anupama Pal | Neoplasia | 2018 |
| [154] | Toni Jauset | Oncotarget | 2018 |
| [155] | Kathleen F. Ludwig | Cancer Research | 2018 |
| [156] | Angela Chou | Gut | 2018 |
| [157] | Xu-Hong Fu | Acta Pharmacologica Sinica | 2018 |
| [158] | Sarah B. Gitto | Molecular Pharmaceutics | 2018 |
| [159] | Francesca Vena | Oncotarget | 2018 |
| [160] | Hongmei Jiang | Molecular Cancer Therapeutics | 2018 |
| [161] | Qi Xu | Cell Death and Disease | 2019 |
| [162] | Zheng Zhong | Oncogene | 2019 |
| [163] | Ping Lu | Oncogene | 2019 |
| [164] | Vivek K. Kashyap | Journal of Experimental & Clinical Cancer Research | 2019 |
| [165] | Aleksandra Adamska | Journal of Experimental & Clinical Cancer Research | 2019 |
| [166] | Aikaterini Emmanouilidi | Journal of Experimental & Clinical Cancer Research | 2019 |
| [167] | Aubrey L. Miller | EBioMedicine | 2019 |
| [168] | Hengyu Lu | Molecular Cancer Therapeutics | 2019 |
| [169] | Aamod S. Dekhne | Molecular Cancer Therapeutics | 2019 |
| [170] | Sejin Chung | Clinical Cancer Research | 2019 |
| [171] | Irmina A. Elliott | Proceedings of the National Academy of Sciences of the USA | 2019 |
| [172] | Raffaela Santoro | Molecular Cancer Therapeutics | 2019 |
| [173] | Kosuke Ogawa | Journal of Hematology & Oncology | 2019 |
| [174] | Mariano Ponz-Sarvise | Clinical Cancer Research | 2019 |
| [175] | Hong Su | Toxicology and Applied Pharmacology | 2019 |
| [176] | Wells S. Brown | Cell Reports Medicine | 2020 |
| [177] | Shiue-Wei Lai | Cells | 2020 |
| [178] | Mei Hua Jin | Cancer Research and Treatment | 2020 |
| [179] | Leonie Konczalla | International Journal of Cancer | 2020 |
| [180] | Jiaqi Wang | Acta Pharmaceutica Sinica B | 2020 |
| [181] | Austin R. Dosch | Molecular Cancer Research | 2020 |
| [182] | Rui Kong | Frontiers in Oncology | 2020 |
| [183] | Aria Vaishnavi | Cell Reports | 2020 |
| [184] | Yan Sun | American Journal of Cancer Research | 2020 |
| [185] | Xin Tong | Journal of Biological Chemistry | 2020 |
| [186] | Olivera Grbovic-Huezo | Proceedings of the National Academy of Sciences of the USA | 2020 |
| [187] | Zhentian Wang | Cancer Cell | 2020 |
| [188] | Rachel A. Caston | Journal of Cellular and Molecular Medicine | 2020 |
| [189] | Yifei Yang | Bioorganic & Medicinal Chemistry | 2020 |
| [190] | Dianyun Ren | Cancer Letters | 2021 |
| [191] | Yazhou Wang | Annals of Translational Medicine | 2021 |
| [192] | T. Y. S. Le Large | Journal of Experimental & Clinical Cancer Research | 2021 |
| [193] | Walaa E. Kattan | Proceedings of the National Academy of Sciences of the USA | 2021 |
| [194] | Shuang Liu | Cancer Chemotherapy and Pharmacology | 2021 |
| [195] | Sai Preethi Nakkina | International Journal of Molecular Sciences | 2021 |
| [196] | Sarah J. Hartman | Frontiers in Oncology | 2021 |
| [197] | Simon Leonhard April-Monn | Cancers | 2021 |
| [198] | Yuchong Zhao | Cancer Cell International | 2021 |
| [199] | Danlin Sun | Molecular Cancer Therapeutics | 2021 |
| [200] | Christian Dubiella | Nature Chemical Biology | 2021 |
| [201] | Zhen Ding | Molecular Medicine Reports | 2021 |
| [202] | Weikun Qian | Journal of Experimental & Clinical Cancer Research | 2021 |
| [203] | Zhengguang Zhang | Inflammopharmocology | 2021 |
| [204] | Josefina Doffo | Proceedings of the National Academy of Sciences of the USA | 2022 |
| [205] | Jiacheng He | Molecules | 2022 |
| [206] | Junfeng Xu | Frontiers in Oncology | 2022 |
| [207] | Daniel R. Principe | Proceedings of the National Academy of Sciences of the USA | 2022 |
| [208] | Ding Xue | Journal of Medicinal Chemistry | 2022 |
| [209] | Simin Qi | Molecular Cancer | 2022 |
| [210] | Shi-Qi Wu | Pharmacological Research | 2022 |
| Reference | First author | Journal | Year |
| [211] | Sumit Kumar | Gut | 2022 |
| [212] | Magdalena Bachmann | Cell Death and Disease | 2022 |
| [213] | Katrin J. Frank | Cell Reports Medicine | 2022 |
| [214] | Maxime Parisotto | Cancers | 2022 |
| [215] | Boya Li | International Journal of Molecular Sciences | 2022 |
| [216] | Mohammad Mahbubul Hoque | Anticancer Research | 2022 |
| [217] | Dat P. Ha | Neoplasia | 2022 |
| [218] | Francisco Quiñonero | Biomedicine & Pharmacotherapy | 2022 |
| [219] | Moises O. Guardado Rivas | PLOS ONE | 2022 |
| [220] | Feifei Cheng | CDD Press | 2022 |
| [221] | Simone Kumstel | PLOS ONE | 2022 |
| [222] | Chunmeng Zhang | Cancer Letters | 2022 |
| [223] | Raffaella Pacchiana | Cancers | 2022 |
| [224] | Zichen Xu | European Journal of Medicinal Chemistry | 2022 |
| [225] | Shijia Wu | Cancer Cell International | 2022 |
| [226] | Yixuan Zhang | Oncogene | 2022 |
| [227] | Penglin Pan | Cancer Research | 2022 |
| [228] | Chengsi Wu | Frontiers in Oncology | 2022 |
| [229] | Tiebo Mao | Cancer Cell International | 2022 |
| [230] | Shuichiro Okamoto | Biomedicine & Pharmacotherapy | 2022 |
| [231] | Chunle Zhao | CDD Press | 2022 |
| [232] | Donatella Delle Cave | Journal of Experimental & Clinical Cancer Research | 2022 |
| [233] | Xiuchao Wang | Cancer Research | 2022 |
| [234] | Alexei Brooun | Toxicology and Applied Pharmacology | 2023 |
| [235] | Chao-Di Chang | Journal of Enzyme Inhibition and Medicinal Chemistry | 2023 |
| [236] | Yuepeng Chen | European Journal of Medicinal Chemistry | 2023 |
| [237] | Yoshihito Morimoto | Journal of Chemotherapy | 2023 |
| [238] | Mi-Hyeon Jeong | JCI Insight | 2023 |
| [239] | Rodrigo Entrialgo-Cadierno | Molecular Cancer | 2023 |
| [240] | Weikang Kong | European Journal of Pharmacology | 2023 |
| [241] | Seoyeon Jeong | Molecular Therapy: Nucleic Acids | 2023 |
| [242] | Niannian Huang | Journal of Medicinal Chemistry | 2023 |
| [243] | Chunhua She | Journal of Experimental & Clinical Cancer Research | 2023 |
| [244] | Eun Jung Lee | CDD Press | 2023 |
| [245] | Qingxiang Lin | British Journal of Pharmacology | 2023 |
| [246] | Chani Stossel | American Association for Cancer Research | 2023 |
| [247] | Andrea Resovi | Journal of Experimental & Clinical Cancer Research | 2023 |
| [248] | Tingting Feng | Molecular Carcinogenesis | 2023 |
| [249] | Feda H. Hamdan | Gut | 2023 |
| [250] | Nina A. Hering | Cancers | 2023 |
| [251] | Myongjae Lee | Molecular Cancer Therapeutics | 2023 |
| [252] | Huang Chen | Clinical Cancer Research | 2023 |
| [253] | Dandan Li | Molecular Cancer Research | 2023 |
| [254] | Yibin Xu | ACS Pharmacology & Translational Science | 2023 |
| [255] | Lei Huang | Clinical and Translational Medicine | 2023 |
| [256] | Maria V. Guijarro | JCI Insight | 2023 |
| [257] | Samantha B. Kemp | Cancer Discovery | 2023 |
| [258] | Inbal Itzhak | Biomolecules | 2023 |
| [259] | Chorong Kim | Anticancer Research | 2023 |
| [260] | Francisco Quiñonero | International Journal of Nanomedicine | 2023 |
| [261] | Krishan Kumar | Cells | 2023 |
| [262] | Razmik Ghukasyan | Clinical Cancer Research | 2023 |
| [263] | Mi Rim Lee | Cancer Science | 2024 |
| [264] | Chu-di Zhang | Translational Oncology | 2024 |
| [265] | Dong Lu | Journal of Medicinal Chemistry | 2024 |
| [266] | Mengxin Li | BBA - Molecular Basis of Disease | 2024 |
| [267] | Giulia D. S. Ferretti 1 | Cell Death & Differentiation | 2024 |
| [268] | Suna Erdem | Molecular Cancer Therapeutics | 2024 |
| [269] | Mir Owais Ayaz | Life Sciences | 2024 |
| [270] | Jichun Gu | Journal of Translational Medicine | 2024 |
| [271] | Jeffrey H. Becker | Cancer Research | 2024 |
| [272] | Dan Su | Cancer Research | 2024 |
| [273] | Satoru Miyazaki | Molecular Oncology | 2024 |
| [274] | Camilla Pecoraro | Journal of Drug Targeting | 2024 |
| [275] | Hui Li | Immunopharmacology and Immunotoxicology | 2024 |
| [276] | Vishnu Kumarasamy | Cancer Research | 2024 |
| [277] | Hayley J. Hawkins | PLOS ONE | 2024 |
| [278] | Wilfried Reichardt | Journal of Magnetic Resonance Imaging | 2024 |
| [279] | Zachary Gao | World Journal of Oncology | 2024 |
| [280] | Caicun Zhou | Cancer Cell | 2024 |
| [281] | Mehrdad Zarei | Cancer Research | 2024 |
| [282] | Mingming Xiao | CDD Press | 2024 |
| Reference | First author | Journal | Year |
| [283] | Weiyu Ge | Molecular Cancer | 2024 |
| [284] | Urszula N. Wasko | Nature | 2024 |
| [285] | Bomin Ku | Cell Reports Medicine | 2024 |
| [286] | Mingming Xiao | Pancreas | 2024 |
| [287] | Jingjing Jiang | Cancer Discovery | 2024 |
| [288] | Simin Wang | EMBO Molecular Medicine | 2024 |
| [289] | Anupriya Singhal | Cancer Discovery | 2024 |
| [290] | Xinyuan Xu | Acta Pharmaceutica Sinica B | 2024 |
| [291] | Yang Li | Acta Pharmaceutica Sinica B | 2025 |
| [292] | Chao-Yang Zeng | Anti-Cancer Drugs | 2025 |
| [293] | Jung Won Chun | BBA - Molecular Basis of Disease | 2025 |
| [294] | Yuri Jobu | Human Cell | 2025 |
| [295] | Zehui Yao | Translational Oncology | 2025 |
| [296] | Zheng Zhang | CDD Press | 2025 |
| [297] | Hala A. Addassi | Carcinogenesis | 2025 |

Table 2. Reporting quality assessment based on the ARRIVE guidelines. Information was fully reported if each criterion of the ARRIVE Essential 10 guideline 2.0 was addressed (fully reported = 2). Information was partially reported if only some of the criteria of the ARRIVE Essential 10 guideline 2.0 were addressed (partially reported = 1). Information was not reported if none of the criteria of the ARRIVE Essential 10 guideline 2.0 were addressed (not reported = 0).

Questions for the ARRIVE Essential guideline 2.0:

A1) *Study design:* Are all experimental and control groups clearly identified? Is the experimental unit clearly identified?

A2) *Sample size:* Is the exact number of experimental units in each group at the start of the study provided? Is the method by which the sample size was chosen explained?

A3) *Inclusion & exclusion criteria:* Are the criteria used for including and excluding animals, experimental units, or data points provided? Are any exclusions of animals, experimental units, or data points reported, or is there a statement indicating that there were no exclusions?

A4) *Randomization:* Is the method by which experimental units were allocated to control and treatment groups described?

A5) *Blinding:* Is it clear whether researchers were aware of, or blinded to, the group allocation at any stage of the experiment or data analysis?

A6) *Outcome measures:* For all experimental outcomes presented, are details provided of exactly what parameter was measured?

A7) *Statistical methods:* Is the statistical approach used to analyses each outcome detailed? Is there a description of any methods used to assess whether data met statistical assumptions?

A8) *Experimental animals:* Are all species of animal used specified? Is the sex of the animals specified? Is at least one of age, weight or developmental stage of the animals specified?

A9) *Experimental procedures:* Are both the timing and frequency with which procedures took place specified? Are details of acclimatization periods to experimental locations provided?

A10) *Results:* Are descriptive statistics for each experimental group provided, with a measure of variability? Is the effect size and confidence interval provided?

| Reference | Year | A1 | A2 | A3 | A4 | A5 | A6 | A7 | A8 | A9 | A10 |
| --- | --- | --- | --- | --- | --- | --- | --- | --- | --- | --- | --- |
| [1] | 1997 | 2 | 0 | 0 | 0 | 0 | 2 | 1 | 2 | 2 | 1 |
| [2] | 1999 | 2 | 0 | 1 | 0 | 0 | 2 | 1 | 1 | 1 | 1 |
| [3] | 1999 | 2 | 0 | 1 | 0 | 0 | 2 | 0 | 1 | 1 | 1 |
| [4] | 2000 | 2 | 1 | 0 | 0 | 0 | 2 | 1 | 1 | 2 | 1 |
| [5] | 2000 | 2 | 1 | 1 | 1 | 0 | 2 | 1 | 1 | 1 | 1 |
| [6] | 2001 | 2 | 0 | 1 | 0 | 0 | 2 | 0 | 1 | 1 | 1 |
| [7] | 2001 | 2 | 0 | 0 | 1 | 0 | 2 | 1 | 1 | 1 | 1 |
| [8] | 2001 | 2 | 0 | 0 | 0 | 0 | 2 | 0 | 1 | 1 | 1 |
| [9] | 2002 | 2 | 1 | 0 | 0 | 0 | 2 | 1 | 1 | 2 | 1 |
| [10] | 2003 | 2 | 1 | 1 | 1 | 0 | 2 | 1 | 2 | 1 | 1 |
| [11] | 2003 | 2 | 1 | 1 | 1 | 0 | 2 | 1 | 1 | 1 | 1 |
| [12] | 2004 | 2 | 1 | 0 | 1 | 0 | 2 | 1 | 1 | 1 | 1 |
| [13] | 2004 | 2 | 1 | 0 | 1 | 0 | 2 | 1 | 1 | 1 | 1 |
| [14] | 2005 | 2 | 1 | 1 | 0 | 0 | 2 | 0 | 1 | 1 | 1 |
| [15] | 2005 | 2 | 1 | 0 | 1 | 0 | 2 | 1 | 1 | 1 | 1 |
| [16] | 2005 | 2 | 1 | 1 | 0 | 0 | 2 | 0 | 1 | 1 | 1 |
| [17] | 2005 | 2 | 1 | 0 | 1 | 0 | 2 | 1 | 1 | 1 | 1 |
| [18] | 2006 | 2 | 1 | 1 | 1 | 0 | 2 | 1 | 1 | 2 | 1 |
| [19] | 2006 | 2 | 1 | 1 | 1 | 0 | 2 | 1 | 1 | 2 | 1 |
| [20] | 2006 | 2 | 1 | 1 | 1 | 0 | 2 | 1 | 1 | 1 | 1 |
| [21] | 2006 | 2 | 1 | 1 | 1 | 0 | 2 | 1 | 1 | 1 | 1 |
| [22] | 2007 | 2 | 1 | 1 | 1 | 0 | 2 | 1 | 1 | 1 | 1 |
| [23] | 2007 | 2 | 1 | 1 | 1 | 0 | 2 | 0 | 1 | 1 | 1 |
| [24] | 2007 | 2 | 1 | 1 | 1 | 0 | 2 | 1 | 1 | 1 | 1 |
| [25] | 2007 | 2 | 1 | 0 | 0 | 0 | 2 | 1 | 1 | 1 | 1 |
| [26] | 2007 | 2 | 1 | 0 | 1 | 0 | 2 | 1 | 1 | 1 | 1 |
| [27] | 2007 | 2 | 1 | 1 | 1 | 0 | 2 | 1 | 1 | 1 | 1 |
| [28] | 2007 | 2 | 1 | 1 | 1 | 0 | 2 | 1 | 1 | 1 | 1 |
| [29] | 2007 | 2 | 1 | 0 | 1 | 0 | 2 | 1 | 1 | 2 | 1 |
| [30] | 2007 | 2 | 1 | 0 | 0 | 0 | 2 | 1 | 1 | 1 | 1 |
| [31] | 2007 | 2 | 1 | 1 | 1 | 0 | 2 | 0 | 1 | 1 | 1 |
| [32] | 2008 | 2 | 1 | 0 | 1 | 0 | 2 | 0 | 1 | 1 | 1 |
| [33] | 2008 | 2 | 1 | 1 | 1 | 0 | 2 | 1 | 1 | 1 | 1 |
| [34] | 2008 | 2 | 1 | 1 | 0 | 0 | 2 | 1 | 2 | 2 | 1 |
| [35] | 2008 | 2 | 0 | 1 | 0 | 0 | 2 | 1 | 1 | 1 | 1 |
| [36] | 2008 | 2 | 1 | 1 | 0 | 0 | 2 | 1 | 1 | 1 | 1 |
| [37] | 2008 | 2 | 1 | 0 | 1 | 0 | 2 | 1 | 1 | 1 | 1 |
| [38] | 2008 | 2 | 1 | 1 | 1 | 0 | 2 | 1 | 2 | 1 | 1 |
| [39] | 2008 | 2 | 1 | 1 | 1 | 0 | 2 | 0 | 1 | 1 | 1 |
| [40] | 2008 | 2 | 1 | 0 | 1 | 0 | 2 | 0 | 1 | 2 | 1 |
| [41] | 2008 | 2 | 1 | 0 | 1 | 0 | 2 | 1 | 1 | 1 | 1 |
| [42] | 2008 | 2 | 1 | 1 | 1 | 0 | 2 | 1 | 1 | 1 | 1 |
| [43] | 2008 | 2 | 1 | 1 | 1 | 0 | 2 | 1 | 1 | 1 | 1 |
| [44] | 2008 | 2 | 1 | 1 | 1 | 0 | 2 | 1 | 1 | 1 | 1 |
| [45] | 2008 | 2 | 1 | 0 | 0 | 0 | 2 | 1 | 1 | 1 | 1 |
| [46] | 2008 | 2 | 1 | 0 | 0 | 0 | 2 | 0 | 1 | 1 | 1 |
| [47] | 2009 | 2 | 0 | 0 | 0 | 0 | 2 | 1 | 1 | 1 | 1 |
| [48] | 2009 | 2 | 1 | 1 | 1 | 0 | 2 | 1 | 1 | 1 | 1 |
| [49] | 2009 | 2 | 1 | 1 | 1 | 0 | 2 | 1 | 2 | 1 | 1 |
| Reference | Year | A1 | A2 | A3 | A4 | A5 | A6 | A7 | A8 | A9 | A10 |
| [50] | 2009 | 2 | 1 | 0 | 1 | 0 | 2 | 1 | 1 | 1 | 1 |
| [51] | 2009 | 2 | 1 | 1 | 1 | 0 | 2 | 1 | 1 | 2 | 1 |
| [52] | 2009 | 2 | 1 | 1 | 1 | 0 | 2 | 1 | 1 | 2 | 1 |
| [53] | 2009 | 2 | 1 | 0 | 0 | 0 | 2 | 1 | 1 | 1 | 1 |
| [54] | 2010 | 2 | 1 | 1 | 0 | 0 | 2 | 1 | 1 | 1 | 1 |
| [55] | 2010 | 2 | 1 | 0 | 1 | 0 | 2 | 1 | 1 | 2 | 1 |
| [56] | 2010 | 2 | 1 | 0 | 0 | 0 | 2 | 1 | 1 | 1 | 1 |
| [57] | 2010 | 2 | 1 | 1 | 1 | 0 | 2 | 1 | 1 | 1 | 1 |
| [58] | 2010 | 2 | 0 | 1 | 0 | 0 | 2 | 1 | 1 | 1 | 1 |
| [59] | 2010 | 2 | 1 | 1 | 0 | 0 | 2 | 0 | 1 | 1 | 1 |
| [60] | 2010 | 2 | 1 | 0 | 1 | 0 | 2 | 1 | 1 | 2 | 1 |
| [61] | 2010 | 2 | 1 | 0 | 1 | 0 | 2 | 1 | 1 | 1 | 1 |
| [62] | 2010 | 2 | 0 | 0 | 0 | 0 | 2 | 0 | 1 | 1 | 1 |
| [63] | 2011 | 2 | 1 | 0 | 1 | 0 | 2 | 1 | 1 | 1 | 1 |
| [64] | 2011 | 2 | 1 | 1 | 2 | 0 | 2 | 1 | 1 | 1 | 1 |
| [65] | 2011 | 2 | 1 | 1 | 1 | 0 | 2 | 1 | 1 | 1 | 1 |
| [66] | 2011 | 2 | 1 | 1 | 1 | 0 | 2 | 1 | 1 | 1 | 1 |
| [67] | 2011 | 2 | 1 | 1 | 0 | 0 | 2 | 1 | 1 | 1 | 1 |
| [68] | 2011 | 2 | 1 | 1 | 1 | 0 | 2 | 1 | 1 | 1 | 1 |
| [69] | 2011 | 2 | 1 | 0 | 1 | 0 | 2 | 1 | 1 | 1 | 1 |
| [70] | 2011 | 2 | 1 | 1 | 1 | 0 | 2 | 0 | 1 | 1 | 1 |
| [71] | 2011 | 2 | 0 | 1 | 0 | 0 | 2 | 1 | 1 | 1 | 1 |
| [72] | 2012 | 2 | 0 | 1 | 1 | 0 | 2 | 1 | 1 | 1 | 1 |
| [73] | 2012 | 2 | 0 | 0 | 0 | 0 | 2 | 1 | 1 | 1 | 1 |
| [74] | 2012 | 2 | 0 | 0 | 0 | 0 | 2 | 1 | 1 | 1 | 1 |
| [75] | 2012 | 2 | 0 | 1 | 0 | 0 | 2 | 1 | 1 | 1 | 1 |
| [76] | 2012 | 2 | 1 | 1 | 0 | 0 | 2 | 0 | 1 | 1 | 1 |
| [77] | 2012 | 2 | 1 | 1 | 1 | 0 | 2 | 1 | 1 | 1 | 1 |
| [78] | 2012 | 2 | 1 | 1 | 0 | 0 | 2 | 1 | 1 | 1 | 1 |
| [79] | 2012 | 2 | 1 | 0 | 2 | 0 | 2 | 1 | 1 | 1 | 1 |
| [80] | 2012 | 2 | 1 | 1 | 1 | 0 | 2 | 1 | 1 | 1 | 1 |
| [81] | 2012 | 2 | 0 | 1 | 1 | 0 | 2 | 1 | 1 | 1 | 1 |
| [82] | 2012 | 2 | 1 | 1 | 1 | 0 | 2 | 1 | 1 | 1 | 1 |
| [83] | 2013 | 2 | 1 | 1 | 0 | 0 | 2 | 1 | 1 | 1 | 1 |
| [84] | 2013 | 2 | 1 | 0 | 0 | 0 | 2 | 1 | 1 | 1 | 1 |
| [85] | 2013 | 2 | 0 | 1 | 1 | 0 | 2 | 1 | 1 | 1 | 1 |
| [86] | 2013 | 2 | 1 | 1 | 0 | 0 | 2 | 1 | 1 | 1 | 1 |
| [87] | 2013 | 2 | 1 | 1 | 0 | 0 | 2 | 1 | 1 | 1 | 1 |
| [88] | 2013 | 2 | 1 | 1 | 1 | 0 | 2 | 1 | 1 | 1 | 1 |
| [89] | 2013 | 2 | 1 | 1 | 1 | 0 | 2 | 0 | 1 | 1 | 1 |
| [90] | 2013 | 2 | 1 | 1 | 1 | 0 | 2 | 1 | 1 | 1 | 1 |
| [91] | 2013 | 2 | 1 | 1 | 0 | 0 | 2 | 1 | 1 | 1 | 1 |
| [92] | 2013 | 2 | 1 | 1 | 1 | 0 | 2 | 1 | 1 | 1 | 1 |
| [93] | 2014 | 2 | 2 | 0 | 0 | 0 | 2 | 1 | 1 | 1 | 1 |
| [94] | 2014 | 2 | 1 | 1 | 1 | 0 | 2 | 0 | 1 | 1 | 1 |
| [95] | 2014 | 2 | 1 | 0 | 2 | 0 | 2 | 1 | 1 | 1 | 1 |
| [96] | 2014 | 2 | 0 | 1 | 1 | 0 | 2 | 1 | 1 | 1 | 1 |
| [97] | 2014 | 2 | 1 | 1 | 0 | 0 | 2 | 0 | 2 | 1 | 1 |
| [98] | 2014 | 2 | 1 | 1 | 1 | 0 | 2 | 1 | 1 | 1 | 1 |
| [99] | 2014 | 2 | 1 | 1 | 1 | 0 | 2 | 1 | 1 | 1 | 1 |
| [100] | 2014 | 2 | 0 | 0 | 0 | 1 | 2 | 0 | 1 | 1 | 1 |
| [101] | 2014 | 2 | 1 | 1 | 1 | 0 | 2 | 1 | 2 | 2 | 1 |
| [102] | 2014 | 2 | 1 | 1 | 1 | 0 | 2 | 1 | 1 | 1 | 1 |
| [103] | 2014 | 2 | 1 | 1 | 1 | 0 | 2 | 1 | 1 | 1 | 1 |
| [104] | 2014 | 2 | 1 | 1 | 1 | 0 | 2 | 0 | 1 | 1 | 1 |
| [105] | 2014 | 2 | 1 | 0 | 0 | 0 | 2 | 1 | 1 | 1 | 1 |
| [106] | 2014 | 2 | 0 | 1 | 1 | 0 | 2 | 1 | 1 | 1 | 1 |
| [107] | 2014 | 2 | 1 | 0 | 0 | 0 | 2 | 1 | 1 | 1 | 1 |
| [108] | 2014 | 2 | 0 | 0 | 0 | 0 | 2 | 1 | 1 | 1 | 1 |
| [109] | 2014 | 2 | 1 | 1 | 1 | 0 | 2 | 1 | 1 | 1 | 1 |
| [110] | 2014 | 2 | 1 | 0 | 0 | 0 | 2 | 0 | 1 | 1 | 1 |
| [111] | 2014 | 2 | 0 | 1 | 1 | 0 | 2 | 0 | 1 | 1 | 1 |
| [112] | 2014 | 2 | 1 | 1 | 0 | 0 | 2 | 1 | 1 | 1 | 1 |
| [113] | 2015 | 2 | 1 | 1 | 1 | 0 | 2 | 2 | 1 | 1 | 1 |
| [114] | 2015 | 2 | 1 | 1 | 0 | 0 | 2 | 1 | 1 | 1 | 1 |
| [115] | 2015 | 2 | 1 | 0 | 0 | 0 | 2 | 1 | 1 | 1 | 1 |
| [116] | 2015 | 2 | 1 | 1 | 0 | 0 | 2 | 1 | 1 | 1 | 1 |
| [117] | 2015 | 2 | 1 | 1 | 0 | 0 | 2 | 1 | 1 | 1 | 1 |
| [118] | 2015 | 2 | 1 | 0 | 0 | 0 | 2 | 1 | 1 | 1 | 1 |
| [119] | 2015 | 2 | 1 | 1 | 1 | 0 | 2 | 1 | 1 | 1 | 1 |
| [120] | 2015 | 2 | 1 | 0 | 0 | 0 | 2 | 1 | 1 | 1 | 1 |
| [121] | 2015 | 2 | 0 | 0 | 1 | 0 | 2 | 1 | 1 | 1 | 1 |
| Reference | Year | A1 | A2 | A3 | A4 | A5 | A6 | A7 | A8 | A9 | A10 |
| [122] | 2015 | 2 | 1 | 1 | 1 | 0 | 2 | 1 | 2 | 1 | 1 |
| [123] | 2015 | 2 | 1 | 1 | 1 | 0 | 2 | 1 | 1 | 1 | 1 |
| [124] | 2015 | 2 | 1 | 1 | 0 | 0 | 2 | 1 | 1 | 1 | 1 |
| [125] | 2015 | 2 | 0 | 0 | 0 | 0 | 2 | 1 | 1 | 2 | 1 |
| [126] | 2015 | 2 | 1 | 0 | 0 | 0 | 2 | 1 | 1 | 1 | 1 |
| [127] | 2015 | 2 | 1 | 0 | 0 | 0 | 2 | 1 | 1 | 1 | 1 |
| [128] | 2016 | 2 | 0 | 1 | 1 | 0 | 2 | 1 | 1 | 1 | 1 |
| [129] | 2016 | 2 | 1 | 1 | 1 | 0 | 2 | 1 | 1 | 1 | 1 |
| [130] | 2016 | 2 | 1 | 0 | 1 | 0 | 2 | 1 | 1 | 1 | 1 |
| [131] | 2016 | 2 | 1 | 1 | 1 | 0 | 2 | 1 | 1 | 1 | 1 |
| [132] | 2016 | 2 | 1 | 0 | 1 | 1 | 2 | 1 | 1 | 1 | 1 |
| [133] | 2016 | 2 | 1 | 1 | 0 | 0 | 2 | 1 | 1 | 1 | 1 |
| [134] | 2016 | 2 | 0 | 1 | 1 | 0 | 2 | 1 | 1 | 1 | 1 |
| [135] | 2016 | 2 | 1 | 1 | 1 | 0 | 2 | 1 | 1 | 1 | 1 |
| [136] | 2017 | 2 | 0 | 1 | 0 | 0 | 2 | 1 | 1 | 1 | 1 |
| [137] | 2017 | 2 | 1 | 1 | 1 | 0 | 2 | 1 | 1 | 1 | 1 |
| [138] | 2017 | 2 | 1 | 1 | 1 | 0 | 2 | 1 | 1 | 1 | 1 |
| [139] | 2017 | 2 | 1 | 1 | 1 | 0 | 2 | 1 | 1 | 1 | 1 |
| [140] | 2017 | 2 | 1 | 0 | 1 | 0 | 2 | 1 | 1 | 1 | 1 |
| [141] | 2017 | 2 | 1 | 1 | 1 | 0 | 2 | 1 | 1 | 1 | 1 |
| [142] | 2017 | 2 | 1 | 1 | 0 | 0 | 2 | 1 | 1 | 1 | 1 |
| [143] | 2017 | 2 | 1 | 0 | 1 | 0 | 2 | 1 | 1 | 1 | 1 |
| [144] | 2017 | 2 | 1 | 1 | 1 | 0 | 2 | 1 | 1 | 1 | 1 |
| [145] | 2017 | 2 | 1 | 0 | 0 | 0 | 2 | 1 | 1 | 1 | 1 |
| [146] | 2017 | 2 | 0 | 1 | 1 | 0 | 2 | 0 | 1 | 1 | 1 |
| [147] | 2017 | 2 | 1 | 0 | 0 | 0 | 2 | 1 | 1 | 1 | 1 |
| [148] | 2017 | 2 | 1 | 0 | 1 | 0 | 2 | 1 | 1 | 1 | 1 |
| [149] | 2017 | 2 | 1 | 1 | 1 | 0 | 2 | 1 | 1 | 1 | 1 |
| [150] | 2018 | 2 | 1 | 1 | 0 | 0 | 2 | 1 | 1 | 1 | 1 |
| [151] | 2018 | 2 | 0 | 0 | 0 | 0 | 2 | 1 | 1 | 1 | 1 |
| [152] | 2018 | 2 | 1 | 1 | 1 | 0 | 2 | 1 | 1 | 1 | 1 |
| [153] | 2018 | 2 | 1 | 1 | 0 | 0 | 2 | 1 | 1 | 1 | 1 |
| [154] | 2018 | 2 | 0 | 0 | 0 | 0 | 2 | 1 | 1 | 1 | 1 |
| [155] | 2018 | 2 | 1 | 1 | 1 | 0 | 2 | 1 | 1 | 1 | 1 |
| [156] | 2018 | 2 | 1 | 1 | 1 | 0 | 2 | 1 | 1 | 1 | 1 |
| [157] | 2018 | 2 | 1 | 1 | 1 | 0 | 2 | 1 | 1 | 1 | 1 |
| [158] | 2018 | 2 | 1 | 0 | 1 | 0 | 2 | 1 | 1 | 1 | 1 |
| [159] | 2018 | 2 | 0 | 1 | 0 | 0 | 2 | 1 | 1 | 2 | 1 |
| [160] | 2018 | 2 | 1 | 1 | 0 | 0 | 2 | 1 | 1 | 1 | 1 |
| [161] | 2019 | 2 | 1 | 1 | 1 | 0 | 2 | 1 | 1 | 1 | 1 |
| [162] | 2019 | 2 | 1 | 1 | 1 | 0 | 2 | 1 | 1 | 1 | 1 |
| [163] | 2019 | 2 | 1 | 1 | 1 | 1 | 2 | 1 | 1 | 1 | 1 |
| [164] | 2019 | 2 | 1 | 1 | 0 | 0 | 2 | 1 | 1 | 1 | 1 |
| [165] | 2019 | 2 | 2 | 1 | 1 | 0 | 2 | 1 | 1 | 1 | 1 |
| [166] | 2019 | 2 | 1 | 1 | 0 | 0 | 2 | 1 | 1 | 1 | 1 |
| [167] | 2019 | 2 | 1 | 1 | 1 | 0 | 2 | 1 | 1 | 1 | 1 |
| [168] | 2019 | 2 | 1 | 1 | 1 | 0 | 2 | 1 | 1 | 1 | 1 |
| [169] | 2019 | 2 | 1 | 0 | 0 | 0 | 2 | 1 | 2 | 1 | 1 |
| [170] | 2019 | 2 | 1 | 1 | 1 | 0 | 2 | 1 | 1 | 1 | 1 |
| [171] | 2019 | 2 | 0 | 1 | 0 | 0 | 2 | 0 | 1 | 1 | 1 |
| [172] | 2019 | 2 | 1 | 1 | 1 | 0 | 2 | 1 | 1 | 1 | 1 |
| [173] | 2019 | 2 | 1 | 1 | 1 | 0 | 2 | 1 | 1 | 1 | 1 |
| [174] | 2019 | 2 | 0 | 1 | 1 | 0 | 2 | 1 | 1 | 1 | 1 |
| [175] | 2019 | 2 | 1 | 1 | 1 | 0 | 2 | 1 | 1 | 1 | 1 |
| [176] | 2020 | 2 | 0 | 1 | 2* | 2** | 2 | 1 | 1 | 2 | 1 |
| [177] | 2020 | 2 | 1 | 0 | 2 | 0 | 2 | 1 | 2 | 1 | 1 |
| [178] | 2020 | 2 | 1 | 1 | 1 | 0 | 2 | 1 | 1 | 1 | 1 |
| [179] | 2020 | 2 | 1 | 1 | 1 | 0 | 2 | 2 | 1 | 1 | 1 |
| [180] | 2020 | 2 | 1 | 1 | 1 | 0 | 2 | 1 | 1 | 1 | 1 |
| [181] | 2020 | 2 | 1 | 0 | 0 | 0 | 2 | 1 | 1 | 1 | 1 |
| [182] | 2020 | 2 | 1 | 0 | 1 | 0 | 2 | 1 | 1 | 1 | 1 |
| [183] | 2020 | 2 | 1 | 1 | 1 | 0 | 2 | 1 | 1 | 1 | 1 |
| [184] | 2020 | 2 | 1 | 0 | 1 | 0 | 2 | 1 | 1 | 1 | 1 |
| [185] | 2020 | 2 | 1 | 0 | 0 | 0 | 2 | 1 | 1 | 1 | 1 |
| [186] | 2020 | 2 | 1 | 0 | 0 | 2** | 2 | 1 | 1 | 1 | 1 |
| [187] | 2020 | 2 | 1 | 1 | 1 | 0 | 2 | 1 | 1 | 1 | 1 |
| [188] | 2020 | 2 | 1 | 1 | 1 | 0 | 2 | 1 | 1 | 1 | 1 |
| [189] | 2020 | 2 | 1 | 1 | 1 | 0 | 2 | 1 | 1 | 1 | 1 |
| [190] | 2021 | 2 | 1 | 1 | 0 | 0 | 2 | 1 | 1 | 1 | 1 |
| [191] | 2021 | 2 | 1 | 0 | 0 | 0 | 2 | 1 | 2 | 1 | 1 |
| [192] | 2021 | 2 | 0 | 1 | 0 | 0 | 2 | 1 | 1 | 1 | 1 |
| [193] | 2021 | 2 | 1 | 0 | 0 | 0 | 2 | 1 | 1 | 1 | 1 |
| Reference | Year | A1 | A2 | A3 | A4 | A5 | A6 | A7 | A8 | A9 | A10 |
| [194] | 2021 | 2 | 1 | 1 | 1 | 0 | 2 | 1 | 1 | 1 | 1 |
| [195] | 2021 | 2 | 1 | 1 | 1 | 0 | 2 | 1 | 1 | 1 | 1 |
| [196] | 2021 | 2 | 1 | 1 | 0 | 0 | 2 | 1 | 1 | 1 | 1 |
| [197] | 2021 | 2 | 1 | 0 | 0 | 0 | 2 | 1 | 1 | 1 | 1 |
| [198] | 2021 | 2 | 1 | 1 | 1 | 0 | 2 | 1 | 1 | 1 | 1 |
| [199] | 2021 | 2 | 0 | 1 | 1 | 0 | 2 | 1 | 1 | 1 | 1 |
| [200] | 2021 | 2 | 1 | 1 | 0 | 0 | 2 | 1 | 1 | 1 | 1 |
| [201] | 2021 | 2 | 1 | 1 | 0 | 0 | 2 | 1 | 2 | 1 | 1 |
| [202] | 2021 | 2 | 1 | 0 | 1 | 0 | 2 | 1 | 1 | 1 | 1 |
| [203] | 2021 | 2 | 1 | 1 | 1 | 0 | 2 | 1 | 1 | 2 | 1 |
| [204] | 2022 | 2 | 1 | 1 | 0 | 0 | 2 | 1 | 1 | 1 | 1 |
| [205] | 2022 | 2 | 1 | 0 | 1 | 0 | 2 | 1 | 1 | 1 | 1 |
| [206] | 2022 | 2 | 1 | 0 | 1 | 0 | 2 | 1 | 1 | 1 | 1 |
| [207] | 2022 | 2 | 1 | 1 | 1 | 0 | 2 | 1 | 1 | 1 | 1 |
| [208] | 2022 | 2 | 1 | 1 | 1 | 0 | 2 | 1 | 1 | 1 | 1 |
| [209] | 2022 | 2 | 1 | 1 | 1 | 0 | 2 | 1 | 1 | 1 | 1 |
| [210] | 2022 | 2 | 1 | 1 | 1 | 0 | 2 | 1 | 2 | 1 | 1 |
| [211] | 2022 | 2 | 1 | 1 | 1 | 0 | 2 | 1 | 1 | 1 | 1 |
| [212] | 2022 | 2 | 2 | 0 | 1 | 0 | 2 | 1 | 2 | 1 | 1 |
| [213] | 2022 | 2 | 1 | 0 | 1 | 0 | 2 | 1 | 1 | 1 | 1 |
| [214] | 2022 | 2 | 1 | 0 | 1 | 0 | 2 | 1 | 2 | 1 | 1 |
| [215] | 2022 | 2 | 1 | 0 | 1 | 0 | 2 | 1 | 2 | 1 | 1 |
| [216] | 2022 | 2 | 1 | 0 | 1 | 0 | 2 | 1 | 2 | 1 | 1 |
| [217] | 2022 | 2 | 1 | 0 | 1 | 0 | 2 | 1 | 2 | 1 | 1 |
| [218] | 2022 | 2 | 1 | 0 | 1 | 0 | 2 | 1 | 2 | 1 | 1 |
| [219] | 2022 | 2 | 1 | 0 | 0 | 0 | 2 | 1 | 2 | 1 | 1 |
| [220] | 2022 | 2 | 1 | 0 | 2 | 0 | 2 | 1 | 2 | 1 | 1 |
| [221] | 2022 | 2 | 1 | 1 | 1 | 0 | 2 | 1 | 0 | 1 | 1 |
| [222] | 2022 | 2 | 1 | 0 | 1 | 0 | 2 | 1 | 1 | 1 | 1 |
| [223] | 2022 | 2 | 1 | 0 | 1 | 0 | 2 | 1 | 1 | 1 | 1 |
| [224] | 2022 | 2 | 1 | 0 | 1 | 0 | 2 | 0 | 0 | 1 | 1 |
| [225] | 2022 | 2 | 1 | 0 | 1 | 0 | 2 | 1 | 2 | 1 | 1 |
| [226] | 2022 | 2 | 1 | 0 | 1 | 0 | 2 | 1 | 2 | 1 | 1 |
| [227] | 2022 | 2 | 1 | 0 | 2 | 0 | 2 | 1 | 0 | 1 | 1 |
| [228] | 2022 | 2 | 0 | 0 | 0 | 0 | 2 | 1 | 1 | 1 | 1 |
| [229] | 2022 | 2 | 1 | 0 | 1 | 0 | 2 | 1 | 2 | 1 | 1 |
| [230] | 2022 | 2 | 1 | 0 | 0 | 0 | 2 | 1 | 2 | 1 | 1 |
| [231] | 2022 | 2 | 1 | 0 | 1 | 0 | 2 | 1 | 2 | 1 | 1 |
| [232] | 2022 | 2 | 1 | 0 | 0 | 0 | 2 | 1 | 2 | 1 | 1 |
| [233] | 2022 | 2 | 1 | 0 | 1 | 1 | 2 | 1 | 2 | 1 | 1 |
| [234] | 2023 | 2 | 1 | 1 | 1 | 0 | 2 | 0 | 2 | 1 | 1 |
| [235] | 2023 | 2 | 0 | 0 | 0 | 0 | 2 | 1 | 2 | 1 | 1 |
| [236] | 2023 | 2 | 1 | 0 | 1 | 0 | 2 | 0 | 2 | 1 | 1 |
| [237] | 2023 | 2 | 1 | 1 | 1 | 0 | 2 | 1 | 2 | 1 | 1 |
| [238] | 2023 | 2 | 1 | 0 | 0 | 0 | 2 | 1 | 2 | 1 | 1 |
| [239] | 2023 | 2 | 2 | 0 | 1 | 2 | 2 | 1 | 1 | 1 | 1 |
| [240] | 2023 | 2 | 1 | 0 | 1 | 0 | 2 | 1 | 1 | 1 | 1 |
| [241] | 2023 | 2 | 1 | 0 | 1 | 0 | 2 | 1 | 2 | 1 | 1 |
| [242] | 2023 | 2 | 1 | 0 | 1 | 0 | 2 | 1 | 2 | 1 | 1 |
| [243] | 2023 | 2 | 1 | 0 | 0 | 0 | 2 | 1 | 1 | 1 | 1 |
| [244] | 2023 | 2 | 1 | 1 | 1 | 0 | 2 | 1 | 2 | 1 | 1 |
| [245] | 2023 | 2 | 0 | 1 | 2 | 2 | 2 | 1 | 2 | 1 | 1 |
| [246] | 2023 | 2 | 0 | 0 | 1 | 0 | 2 | 1 | 2 | 1 | 1 |
| [247] | 2023 | 2 | 1 | 0 | 0 | 0 | 2 | 1 | 2 | 1 | 1 |
| [248] | 2023 | 2 | 1 | 0 | 0 | 0 | 2 | 1 | 2 | 1 | 1 |
| [249] | 2023 | 2 | 1 | 1 | 2 | 2 | 2 | 1 | 2 | 1 | 1 |
| [250] | 2023 | 2 | 1 | 1 | 1 | 0 | 2 | 1 | 2 | 1 | 1 |
| [251] | 2023 | 2 | 1 | 1 | 2 | 0 | 2 | 1 | 2 | 1 | 1 |
| [252] | 2023 | 2 | 1 | 0 | 1 | 0 | 2 | 1 | 2 | 1 | 1 |
| [253] | 2023 | 2 | 1 | 0 | 0 | 1 | 2 | 1 | 0 | 1 | 1 |
| [254] | 2023 | 2 | 1 | 0 | 1 | 0 | 2 | 1 | 2 | 1 | 1 |
| [255] | 2023 | 2 | 0 | 0 | 1 | 0 | 2 | 1 | 2 | 1 | 1 |
| [256] | 2023 | 2 | 1 | 0 | 1 | 0 | 2 | 1 | 2 | 1 | 1 |
| [257] | 2023 | 2 | 1 | 0 | 1 | 0 | 2 | 1 | 1 | 1 | 1 |
| [258] | 2023 | 2 | 1 | 1 | 1 | 2** | 2 | 1 | 1 | 1 | 1 |
| [259] | 2023 | 2 | 1 | 0 | 1 | 0 | 2 | 1 | 2 | 1 | 1 |
| [260] | 2023 | 2 | 1 | 0 | 1 | 0 | 2 | 1 | 2 | 2 | 1 |
| [261] | 2023 | 2 | 0 | 1 | 1 | 0 | 2 | 1 | 2 | 1 | 1 |
| [262] | 2023 | 2 | 1 | 0 | 1 | 0 | 2 | 1 | 2 | 1 | 1 |
| [263] | 2024 | 2 | 0 | 0 | 0 | 2 | 2 | 1 | 2 | 1 | 1 |
| [264] | 2024 | 2 | 1 | 0 | 1 | 0 | 2 | 1 | 2 | 1 | 1 |
| [265] | 2024 | 2 | 1 | 0 | 1 | 0 | 2 | 0 | 2 | 1 | 1 |
| Reference | Year | A1 | A2 | A3 | A4 | A5 | A6 | A7 | A8 | A9 | A10 |
| [266] | 2024 | 2 | 1 | 0 | 1 | 0 | 2 | 1 | 1 | 1 | 1 |
| [267] | 2024 | 2 | 2 | 0 | 1 | 2** | 2 | 1 | 2 | 1 | 1 |
| [268] | 2024 | 2 | 0 | 0 | 0 | 0 | 2 | 1 | 1 | 1 | 1 |
| [269] | 2024 | 2 | 1 | 0 | 1 | 0 | 2 | 1 | 2 | 1 | 1 |
| [270] | 2024 | 2 | 1 | 0 | 1 | 2 | 2 | 1 | 1 | 1 | 1 |
| [271] | 2024 | 2 | 0 | 0 | 1 | 0 | 2 | 1 | 1 | 1 | 1 |
| [272] | 2024 | 2 | 1 | 0 | 0 | 0 | 2 | 1 | 1 | 1 | 1 |
| [273] | 2024 | 2 | 1 | 0 | 0 | 0 | 2 | 1 | 2 | 1 | 1 |
| [274] | 2024 | 2 | 1 | 0 | 1 | 0 | 2 | 1 | 0 | 1 | 1 |
| [275] | 2024 | 2 | 1 | 0 | 0 | 0 | 2 | 1 | 1 | 1 | 1 |
| [276] | 2024 | 2 | 1 | 1 | 1 | 0 | 2 | 1 | 1 | 1 | 1 |
| [277] | 2024 | 2 | 0 | 0 | 1 | 2** | 2 | 1 | 1 | 1 | 1 |
| [278] | 2024 | 2 | 0 | 0 | 1 | 0 | 2 | 1 | 0 | 1 | 1 |
| [279] | 2024 | 2 | 0 | 0 | 1 | 0 | 2 | 1 | 2 | 1 | 1 |
| [280] | 2024 | 2 | 1 | 0 | 1 | 0 | 2 | 1 | 2 | 1 | 1 |
| [281] | 2024 | 2 | 1 | 0 | 1 | 0 | 2 | 1 | 2 | 1 | 1 |
| [282] | 2024 | 2 | 1 | 0 | 0 | 0 | 2 | 1 | 1 | 1 | 1 |
| [283] | 2024 | 2 | 1 | 0 | 1 | 0 | 2 | 1 | 2 | 1 | 1 |
| [284] | 2024 | 2 | 1 | 0 | 1 | 0 | 2 | 1 | 2 | 1 | 1 |
| [285] | 2024 | 2 | 1 | 0 | 1 | 0 | 2 | 1 | 2 | 1 | 1 |
| [286] | 2024 | 2 | 1 | 0 | 0 | 0 | 2 | 1 | 1 | 1 | 1 |
| [287] | 2024 | 2 | 1 | 0 | 2 | 0 | 2 | 1 | 2 | 1 | 1 |
| [288] | 2024 | 2 | 1 | 0 | 1 | 2** | 2 | 1 | 2 | 1 | 1 |
| [289] | 2024 | 2 | 1 | 0 | 1 | 2** | 2 | 1 | 0 | 1 | 1 |
| [290] | 2024 | 2 | 1 | 0 | 0 | 0 | 2 | 1 | 2 | 1 | 1 |
| [291] | 2025 | 2 | 1 | 0 | 1 | 0 | 2 | 1 | 0 | 1 | 1 |
| [292] | 2025 | 2 | 1 | 0 | 0 | 0 | 2 | 1 | 2 | 1 | 1 |
| [293] | 2025 | 2 | 1 | 0 | 1 | 0 | 2 | 1 | 2 | 1 | 1 |
| [294] | 2025 | 2 | 1 | 0 | 0 | 0 | 2 | 1 | 2 | 1 | 1 |
| [295] | 2025 | 2 | 1 | 0 | 0 | 0 | 2 | 1 | 2 | 1 | 1 |
| [296] | 2025 | 2 | 1 | 0 | 1 | 0 | 2 | 1 | 2 | 1 | 1 |
| [297] | 2025 | 2 | 0 | 0 | 0 | 0 | 2 | 1 | 2 | 1 | 1 |

*Explicitly mentioned that no randomization was conducted.

**Explicitly mentioned that no blinding was conducted**.**

Table 3. Main mouse strains found in the 297 articles (756 individual studies) categorized according to the genetic background, the type of genetics and the characteristics hair, T cells, B cells, NK cells.

| Strain | Genetic background | Type of genetics | Hair | T cells | B cells | NK cells |
| --- | --- | --- | --- | --- | --- | --- |
| BALB/c nu/nu, CD1 nu/nu, Foxn1 nu/nu, J:NU, NCr nu/nu, NMRI nu/nu, NU/NU-nu BR, Swiss nu/nu | Foxn1^nu^ | mutation | no | no | yes | yes |
| IDO KO | Ido1^tm1Alm^ | KO | yes | yes | yes | yes |
| FVB/NJ | Pde6b^rd1^ | homozygous allele | yes | yes | yes | yes |
| B6/NPI | PNP | mutation | yes | no | no | yes |
| Rag2^-/-^, Il2Ry^-/-^ | Rag2^tm1Fwa^II2rg^tm1Rsky^ | KO | yes | defective | defective | no |
| NCG | Prkdc^em26Cd52^Il2rg^em26Cd22^ | mutation | yes | no | no | no |
| SCID, SCID/NOD | Prkdc^scid^ | mutation | yes | no | no | yes |
| SCID/beige | Prkdc^scid^, Lyst^bg^ | mutation | yes | no | no | defective |
| NSG | Prkdc^scid^, Il2rg^tm1WjI^ | mutation | yes | no | no | no |
| C57BL/6 | wt | wt | yes | yes | yes | yes |

KO = knockout, wt = wild type, PNP = purine-nucleoside phosphorylase

Table 4. Multivariable meta-regression analyses for primary tumor volume regression dataset.

Mixed-Effects Model (k = 374; tau^2^ estimator: REML).

tau^2^ = 556.2553 (SE = 60.5871), I^2^ = 87.56%, R^2^ = 14.43%.

Test for Residual Heterogeneity: QE(df = 323) = 2490.0393, p < 0.0001.

Test of Moderators (coefficients 2:51): F(df1 = 50, df2 = 323) = 2.0739, p = 0.0307.

| Variable | Estimate (coefficient) | SE | P value | 95% confidence intervals | | t value |
| --- | --- | --- | --- | --- | --- | --- |
| Intercept | 10.1672 | 30.6009 | 0.7399 | -50.0351 | 70.3694 | 0.3323 |
| Genetic background (other) | 10.2414 | 8.4139 | 0.2244 | -6.3116 | 26.7945 | 1.2172 |
| Genetic background (Prkdcscid, Il2rgtm1WjI) | -8.5759 | 7.1557 | 0.2316 | -22.6536 | 5.5019 | -1.1985 |
| Genetic background (Prkdcscid) | -2.144 | 5.1588 | 0.678 | -12.2931 | 8.0051 | -0.4156 |
| Genetic background (wt) | 11.3958 | 14.0175 | 0.4168 | -16.1814 | 38.973 | 0.813 |
| Sex (female/male) | 16.4915 | 14.3576 | 0.2516 | -11.7548 | 44.7378 | 1.1486 |
| Sex (male) | 2.3393 | 4.5107 | 0.6044 | -6.5348 | 11.2133 | 0.5186 |
| Sex (n.m.) | -5.9001 | 4.9597 | 0.2351 | -15.6575 | 3.8573 | -1.1896 |
| Randomization (yes) | 4.6228 | 4.1951 | 0.2713 | -3.6304 | 12.876 | 1.102 |
| Blinding (no) | 26.9857 | 9.3519 | 0.0042** | 8.5874 | 45.3839 | 2.8856 |
| Blinding (yes) | -3.0967 | 8.5619 | 0.7178 | -19.9407 | 13.7474 | -0.3617 |
| Baseline tumor size (100-150) | 6.0168 | 10.1417 | 0.5534 | -13.9353 | 25.969 | 0.5933 |
| Baseline tumor size (100-200) | -2.5025 | 8.9611 | 0.7802 | -20.132 | 15.1269 | -0.2793 |
| Baseline tumor size (100-250) | 9.825 | 12.3231 | 0.4259 | -14.4186 | 34.0686 | 0.7973 |
| Baseline tumor size (100-300) | -15.9614 | 10.7328 | 0.138 | -37.0765 | 5.1537 | -1.4872 |
| Baseline tumor size (150-200) | 4.524 | 7.9711 | 0.5707 | -11.1579 | 20.2059 | 0.5675 |
| Baseline tumor size (200) | 1.4215 | 7.4253 | 0.8483 | -13.1865 | 16.0295 | 0.1914 |
| Baseline tumor size (200-250) | -6.078 | 9.3148 | 0.5145 | -24.4033 | 12.2472 | -0.6525 |
| Baseline tumor size (22-126) | 34.6071 | 11.5005 | 0.0028** | 11.9817 | 57.2325 | 3.0092 |
| Baseline tumor size (300-500) | -18.2889 | 15.9434 | 0.2522 | -49.655 | 13.0771 | -1.1471 |
| Baseline tumor size (50-100) | 5.9951 | 7.5489 | 0.4277 | -8.856 | 20.8463 | 0.7942 |
| Baseline tumor size (80-120) | -20.8163 | 13.4471 | 0.1226 | -47.2713 | 5.6388 | -1.548 |
| Baseline tumor size (n.m.) | 0.7192 | 6.1534 | 0.907 | -11.3865 | 12.8249 | 0.1169 |
| Baseline tumor size (other) | 15.8539 | 7.2463 | 0.0294* | 1.5979 | 30.1099 | 2.1879 |
| Baseline tumor size (palpable) | -14.694 | 12.3178 | 0.2338 | -38.9272 | 9.5392 | -1.1929 |
| Transplantation (metastatic) | -35.5399 | 23.6786 | 0.1344 | -82.1236 | 11.0438 | -1.5009 |
| Transplantation (orthotopic) | -2.769 | 7.2477 | 0.7027 | -17.0278 | 11.4897 | -0.3821 |
| Tumor model (PDX) | 33.813 | 30.9691 | 0.2757 | -27.1137 | 94.7397 | 1.0918 |
| Tumor model (syngraft) | -22.5955 | 32.1242 | 0.4823 | -85.7946 | 40.6036 | -0.7034 |
| Tumor model (xenograft) | 36.0626 | 27.9276 | 0.1975 | -18.8803 | 91.0055 | 1.2913 |
| Injected cell type (BxPC-3) | 11.4928 | 10.0133 | 0.2519 | -8.2066 | 31.1923 | 1.1478 |
| Injected cell type (Capan-1) | 14.3446 | 11.2722 | 0.2041 | -7.8315 | 36.5207 | 1.2726 |
| Injected cell type (HPAF-2) | 32.2097 | 11.7595 | 0.0065** | 9.0748 | 55.3446 | 2.739 |
| Injected cell type (L3.6pl) | 26.8399 | 13.6596 | 0.0503 | -0.0332 | 53.7129 | 1.9649 |
| Injected cell type (MiaPaCa-2) | 4.8561 | 8.2124 | 0.5547 | -11.3004 | 21.0127 | 0.5913 |
| Injected cell type (other) | 16.9022 | 7.8394 | 0.0318* | 1.4796 | 32.3249 | 2.1561 |
| Injected cell type (Panc-02) | 53.7514 | 16.1647 | 0.001*** | 21.95 | 85.5529 | 3.3252 |
| Injected cell type (PANC-1) | 17.2194 | 8.7517 | 0.05* | 0.0019 | 34.4369 | 1.9675 |
| Injected cell type (PDX) | 10.9152 | 16.7791 | 0.5158 | -22.0949 | 43.9254 | 0.6505 |
| Injected cell type (SW-1990) | 9.9465 | 11.9429 | 0.4056 | -13.5493 | 33.4422 | 0.8328 |
| Inhibitor category (selective non-kinase SMI) | -11.934 | 10.7216 | 0.2665 | -33.027 | 9.1591 | -1.1131 |
| Inhibitor category (selective intracellular kinase SMI, selective non-kinase SMI) | -35.8251 | 18.5019 | 0.0537 | -72.2247 | 0.5744 | -1.9363 |
| Inhibitor category (selective intracellular kinase SMI) | -16.0301 | 10.9864 | 0.1455 | -37.6439 | 5.5838 | -1.4591 |
| Inhibitor category (selective receptor-related kinase SMI) | -23.1109 | 11.9965 | 0.0549 | -46.7121 | 0.4903 | -1.9265 |
| Inhibitor category (selective receptor-related kinase SMI, selective intracellular kinase SMI) | -25.0053 | 15.1429 | 0.0997 | -54.7964 | 4.7858 | -1.6513 |
| Application route drug (i.v.) | -15.1351 | 13.2177 | 0.253 | -41.1387 | 10.8686 | -1.1451 |
| Application route drug (n.m.) | -11.4834 | 8.9332 | 0.1995 | -29.058 | 6.0911 | -1.2855 |
| Application route drug (other) | 17.1276 | 20.1741 | 0.3965 | -22.5617 | 56.8168 | 0.849 |
| Application route drug (p.o.) | -7.5595 | 3.8471 | 0.0503 | -15.128 | 0.0091 | -1.965 |
| Application route drug (s.c.) | 11.5262 | 14.5138 | 0.4277 | -17.0273 | 40.0797 | 0.7942 |
| Application route cells (peri.panc.) | 32.12 | 60.9896 | 0.5988 | -87.867 | 152.1071 | 0.5266 |

Significant codes: ≤0.0001***, ≤0.01**, ≤0.05*, n.m. = not mentioned

Table 5. Multivariable meta-regression analyses for primary tumor weight regression dataset.

Mixed-Effects Model (k = 194; tau^2^ estimator: REML).

tau^2^ = 249.0265 (SE = 53.0712), I^2^ =66.19%, R^2^ =32.32%.

Test for Residual Heterogeneity: QE(df = 155) = 452.9028, p-value < 0.0001.

Test of Moderators (coefficients 2:39): F(df1 = 38, df2 = 155) = 2.2893, p-value = 0.0002.

| Variable | Estimate (coefficient) | SE | P value | 95% confidence intervals | | t value |
| --- | --- | --- | --- | --- | --- | --- |
| Intercept | 11.8582 | 23.7657 | 0.6185 | -35.0884 | 58.8047 | 0.499 |
| Genetic background (other) | 20.0769 | 11.7153 | 0.0886 | -3.0653 | 43.2192 | 1.7137 |
| Genetic background (Prkdc^scid^, Il2rgtm1WjI) | 2.6412 | 8.2519 | 0.7493 | -13.6596 | 18.9419 | 0.3201 |
| Genetic background (Prkdc^scid^) | -7.0362 | 7.7456 | 0.3651 | -22.3367 | 8.2643 | -0.9084 |
| Genetic background (wt) | -15.1246 | 19.8489 | 0.4472 | -54.3339 | 24.0846 | -0.762 |
| Sex (female/male) | 24.6314 | 15.5395 | 0.115 | -6.0652 | 55.3279 | 1.5851 |
| Sex (male) | 10.711 | 5.1187 | 0.038* | 0.5995 | 20.8224 | 2.0925 |
| Sex (n.m.) | 1.8102 | 5.5236 | 0.7436 | -9.1011 | 12.7216 | 0.3277 |
| Transplantation (metastatic) | -55.0664 | 54.8229 | 0.3167 | -163.3629 | 53.2301 | -1.0044 |
| Transplantation (orthotopic) | -1.4035 | 6.5114 | 0.8296 | -14.2661 | 11.4591 | -0.2155 |
| Tumor model (PDX) | 34.3822 | 20.1465 | 0.0899 | -5.415 | 74.1794 | 1.7066 |
| Tumor model (syngraft) | 25.8611 | 23.575 | 0.2744 | -20.7086 | 72.4308 | 1.097 |
| Tumor model (xenograft) | 20.7735 | 17.7729 | 0.2443 | -14.3348 | 55.8819 | 1.1688 |
| Injected cell type (BxPC-3) | 14.8328 | 13.1689 | 0.2618 | -11.1808 | 40.8465 | 1.1264 |
| Injected cell type (L3.6pl) | 0.3913 | 10.7083 | 0.9709 | -20.7616 | 21.5443 | 0.0365 |
| Injected cell type (MiaPaCa-2) | 18.4734 | 9.2744 | 0.0481* | 0.153 | 36.7938 | 1.9919 |
| Injected cell type (other) | 15.5105 | 8.7189 | 0.0772 | -1.7128 | 32.7338 | 1.7789 |
| Injected cell type (Panc-02) | 9.411 | 15.406 | 0.5422 | -21.0219 | 39.8439 | 0.6109 |
| Injected cell type (PANC-1) | 26.9289 | 9.5252 | 0.0053** | 8.113 | 45.7448 | 2.8271 |
| Inhibitor category (other) | -5.1797 | 15.8525 | 0.7443 | -36.4946 | 26.1351 | -0.3267 |
| Inhibitor category (selective intracellular kinase SMI) | -6.4424 | 13.9636 | 0.6452 | -34.0258 | 21.141 | -0.4614 |
| Inhibitor category (selective non-kinase SMI) | -2.9893 | 13.2107 | 0.8213 | -29.0856 | 23.1069 | -0.2263 |
| Inhibitor category (selective receptor-related kinase SMI) | -0.7573 | 14.6644 | 0.9589 | -29.725 | 28.2105 | -0.0516 |
| Application route drug (n.m.) | -22.1463 | 7.1419 | 0.0023** | -36.2543 | -8.0383 | -3.1009 |
| Application route drug (other) | 20.833 | 9.2805 | 0.0262* | 2.5004 | 39.1656 | 2.2448 |
| Application route drug (p.o.) | -4.7789 | 4.5488 | 0.2951 | -13.7646 | 4.2068 | -1.0506 |
| Randomization (yes) | -4.0934 | 5.0962 | 0.4231 | -14.1603 | 5.9736 | -0.8032 |
| Blinding (no) | 5.5558 | 14.2004 | 0.6962 | -22.4955 | 33.607 | 0.3912 |
| Blinding (yes) | -8.2537 | 11.3227 | 0.4671 | -30.6205 | 14.1131 | -0.729 |
| Baseline tumor size (100-250) | 23.0134 | 12.7186 | 0.0723 | -2.1108 | 48.1377 | 1.8094 |
| Baseline tumor size (120-300) | -7.1769 | 10.7845 | 0.5067 | -28.4805 | 14.1267 | -0.6655 |
| Baseline tumor size (20-80) | 49.2575 | 30.7492 | 0.1112 | -11.4842 | 109.9991 | 1.6019 |
| Baseline tumor size (200) | 4.8801 | 10.3398 | 0.6376 | -15.545 | 25.3052 | 0.472 |
| Baseline tumor size (50-100) | -2.0931 | 8.9498 | 0.8154 | -19.7724 | 15.5862 | -0.2339 |
| Baseline tumor size (90-150) | 3.7936 | 12.1619 | 0.7555 | -20.2307 | 27.818 | 0.3119 |
| Baseline tumor size (n.m.) | 3.0526 | 6.1025 | 0.6176 | -9.0023 | 15.1074 | 0.5002 |
| Baseline tumor size (other) | -3.2205 | 8.1877 | 0.6946 | -19.3944 | 12.9533 | -0.3933 |
| Baseline tumor size (palpable) | -1.0948 | 10.5771 | 0.9177 | -21.9887 | 19.7992 | -0.1035 |
| Application route cells (other) | -14.4519 | 15.0827 | 0.3395 | -44.2462 | 15.3423 | -0.9582 |

Significant codes: ≤0.0001***, ≤0.01**, ≤0.05*, n.m. = not mentioned

Table 6. Multivariable meta-regression analyses for primary tumor area regression dataset.

Mixed-Effects Model (k = 28; method: REML).

Variance components:

estim sqrt nlvls fixed factor

sigma^2.1^ 3.4014 1.8443 16 no number

sigma^2.2^ 0.0000 0.0001 28 no index

Test for Residual Heterogeneity: QE(df = 11) = 40.9314, p-value < 0.0001.

Test of Moderators (coefficients 2:17): F(df1 = 16, df2 = 11) = 0.4944, p-value = 0.9027.

| Variable | Estimate (coefficient) | SE | P value | 95% confidence intervals | | t value |
| --- | --- | --- | --- | --- | --- | --- |
| Intercept | 1.6493 | 8.5337 | 0.8503 | -17.1332 | 20.4317 | 0.1933 |
| Genetic background (Foxn1^nu^) | 5.255 | 4.2331 | 0.2403 | -4.062 | 14.572 | 1.2414 |
| Genetic background (Prkdc^scid^) | 6.6025 | 5.7996 | 0.2791 | -6.1624 | 19.3674 | 1.1384 |
| Genetic background (wt) | 6.9515 | 6.8865 | 0.3344 | -8.2054 | 22.1085 | 1.0095 |
| Sex (female/male) | -0.0733 | 4.7084 | 0.9879 | -10.4365 | 10.2899 | -0.0156 |
| Sex (male) | -6.351 | 6.2341 | 0.3302 | -20.0723 | 7.3702 | -1.0188 |
| Sex (n.m.) | 4.5411 | 4.5536 | 0.3401 | -5.4813 | 14.5635 | 0.9973 |
| Transplantation (metastatic) | -3.0054 | 4.8453 | 0.5477 | -13.6699 | 7.6591 | -0.6203 |
| Transplantation (orthotopic) | -1.9591 | 4.1245 | 0.6441 | -11.037 | 7.1187 | -0.475 |
| Tumor model (xenograft) | -1.8215 | 4.2116 | 0.6737 | -11.0911 | 7.4481 | -0.4325 |
| Injected cell type (PANC-1) | 1.9151 | 1.5606 | 0.2454 | -1.5197 | 5.3499 | 1.2272 |
| Injected cell type (SW-1990) | 0.5427 | 1.8603 | 0.7759 | -3.5518 | 4.6372 | 0.2917 |
| Inhibitor category (selective intracellular kinase SMI) | -2.6135 | 3.0559 | 0.4107 | -9.3396 | 4.1125 | -0.8552 |
| Inhibitor category (selective non-kinase SMI) | 1.9551 | 4.0703 | 0.6404 | -7.0035 | 10.9137 | 0.4803 |
| Inhibitor category (selective receptor-related kinase SMI, selective intracellular kinase SMI) | -2.3586 | 5.2221 | 0.6603 | -13.8524 | 9.1352 | -0.4517 |
| Application route drug (p.o.) | -2.4391 | 1.5185 | 0.1365 | -5.7812 | 0.903 | -1.6063 |
| Application route cells (other) | 2.8326 | 1.8824 | 0.1606 | -1.3106 | 6.9757 | 1.5047 |

n.m. = not mentioned

**Supplemental Figures:**


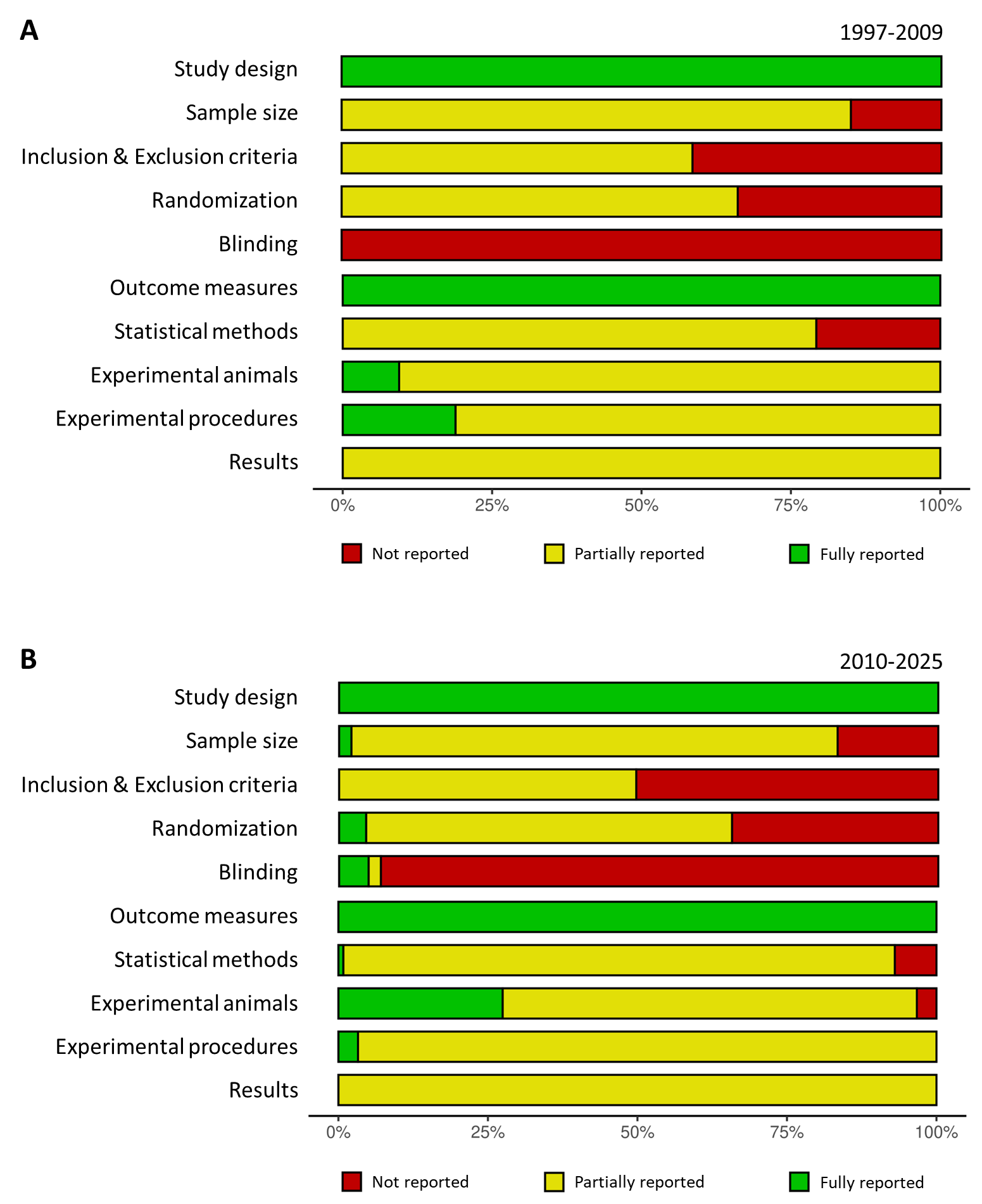


Figure 1. Summary plots of the reporting quality assessment based on ARRIVE Essential 10 guideline 2.0 generated by the *robvis tool*. (McGuinness, L. A. & Higgins, J. P. T. Risk-of-bias VISualization (robvis): An R package and Shiny web app for visualizing risk-of-bias assessments. Res Synth Methods n/a, 2020). Red represents unreported information, yellow partially reported information and green fully reported information according to the guidelines. Since the ARRIVE Essential 10 guideline was implemented in 2010, the year 2010 was selected as cut-off for the reporting quality assessment to investigate a potential difference in reporting quality over time. A) Summary plot of 53 articles published between 1997 and 2009. B) Summary plot 244 articles published between 2010 and 2025.


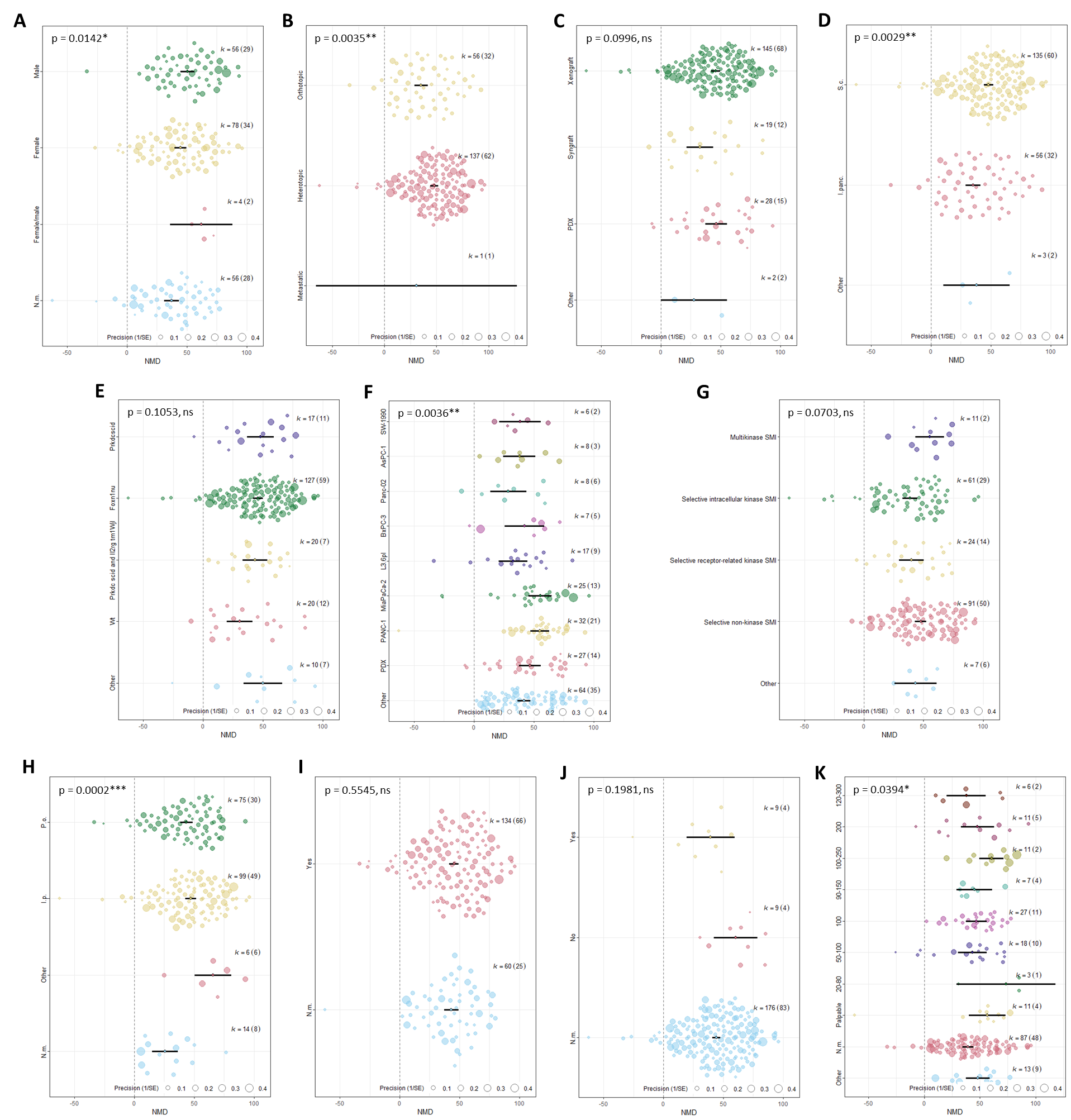


Figure 2. Orchard plots of univariate meta-regression analysis for primary tumor weight regression based on A) sex, B) transplantation, C) tumor graft, D) tumor cell/tissue application route, E) genetic background of mice, F) injected tumor cell/tissue type, G) inhibitory category (‘other’ defined here as selective receptor-related kinase and intracellular SMI and selective intracellular kinase and non-kinase SMI), H) drug application route (‘other’ defined here as n.m. and s.c.). I) randomization, J) blinding, and K) baseline tumor size. The thick black line indicates 95% confidence intervals (CI), the extended black line indicates 95% prediction intervals (PI). Each circle represents one comparison and its diameter designates the weight each comparison carries in the pooled effect size based on precision. The number of comparisons included in each subgroup of interest is labelled by *k*, and with the number of individual studies labelled in brackets. P-values are denoted as follows: >0.05 (ns), ≤0.05 (*), ≤0.005 (**), and ≤0.0005 (***).


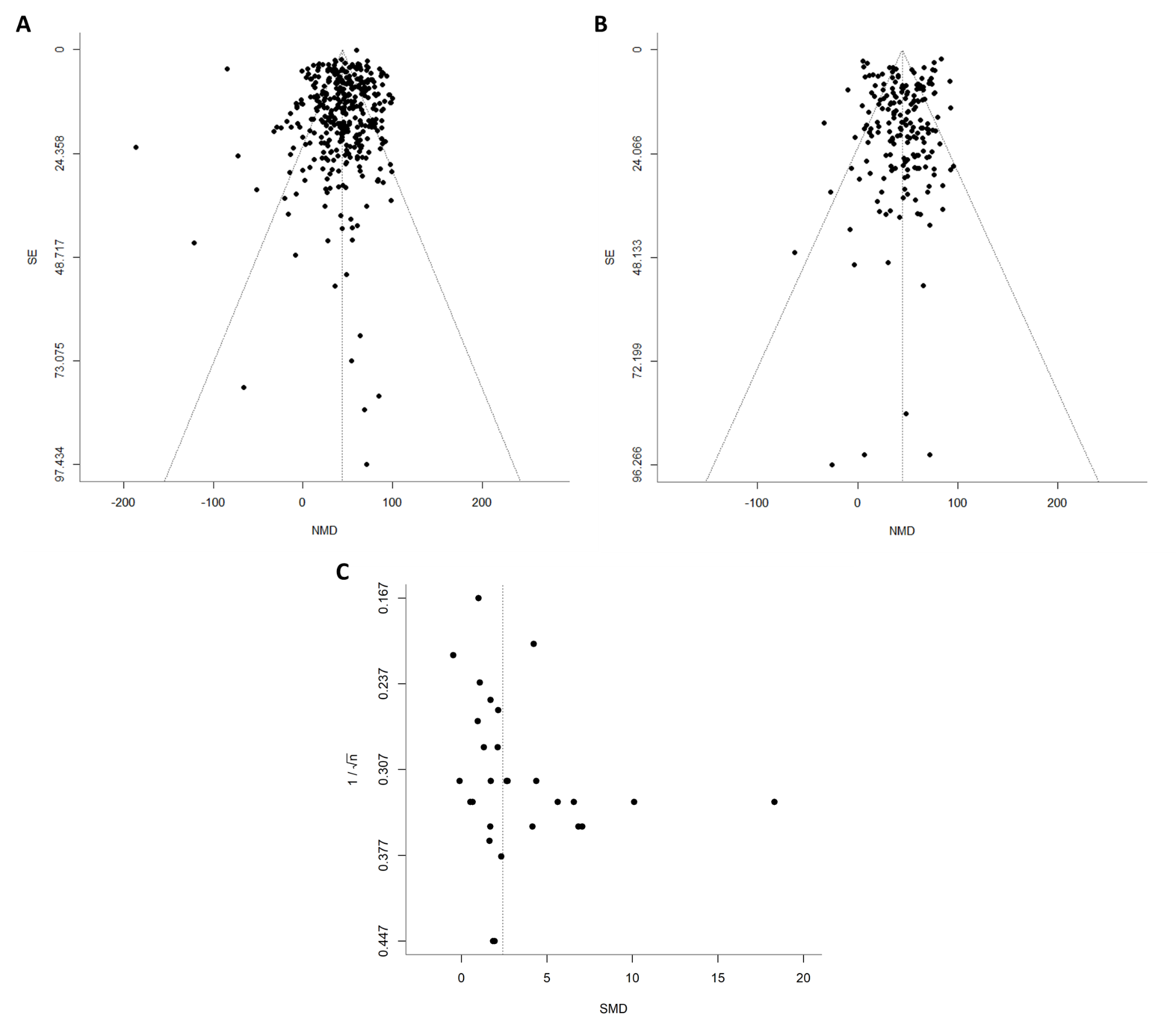


Figure 3. Funnel plots of small molecule inhibitor therapy outcomes on pancreatic cancer. A) Primary tumor volume regression, B) primary tumor weight regression, and C) primary tumor area regression. Each black dot represents an individual study. In panels A and B, the x-axis shows the observed effect size as normalized mean difference (NMD) and the y-axis represents the standard error (SE), with diagonal lines indicating the 95% confidence limits. In panel C, the x-axis shows the effect size as standardized mean difference (SMD), and the y-axis corresponds to 1/√n. The vertical dashed line represents the overall effect size estimated under the random-effects model (NMD_A_ = 35.75%; NMD_B_ = 39.49%; SMD_C_= 2.14). The apparent asymmetry in panel C should be regarded as descriptive only, given the limited number of studies, high heterogeneity, and non-independence of effects.

**Reference list of the included studies in systematic review and meta-analyses:**

1. Zervos, E. E., Norman, J. G., Gower, W. R., Franz, M. G. & Rosemurgy, A. S. Matrix metalloproteinase inhibition attenuates human pancreatic cancer growth in vitro and decreases mortality and tumorigenesis in vivo. *J Surg Res* 69, 367–371 (1997).

2. Saito, A. *et al.* A synthetic inhibitor of histone deacetylase, MS-27-275, with marked in vivo antitumor activity against human tumors. *Proc Natl Acad Sci U S A* 96, 4592–4597 (1999).

3. Prevost, G. P. *et al.* Inhibition of human tumor cell growth in vitro and in vivo by a specific inhibitor of human farnesyltransferase: BIM-46068. *Int J Cancer* 83, 283–287 (1999).

4. Jimenez, R. E. *et al.* Effect of matrix metalloproteinase inhibition on pancreatic cancer invasion and metastasis: an additive strategy for cancer control. *Ann Surg* 231, 644–54 (2000).

5. Haq, M., Shafii, A., Zervos, E. E. & Rosemurgy, A. S. Addition of matrix metalloproteinase inhibition to conventional cytotoxic therapy reduces tumor implantation and prolongs survival in a murine model of human pancreatic cancer. *Cancer Res* 60, 3207–3211 (2000).

6. Prevost, G. P. *et al.* Inhibition of human tumor cell growth in vivo by an orally bioavailable inhibitor of human farnesyltransferase, BIM-46228. *Int J Cancer* 91, 718–722 (2001).

7. End, D. W. *et al.* Characterization of the antitumor effects of the selective farnesyl protein transferase inhibitor R115777 in vivo and in vitro. *Cancer Res* 61, 131–137 (2001).

8. Lobell, R. B. *et al.* Evaluation of farnesyl:protein transferase and geranylgeranyl:protein transferase inhibitor combinations in preclinical models. *Cancer Res* 61, 8758–8768 (2001).

9. Tong, W.-G., Ding, X.-Z., Witt, R. C. & Adrian, T. E. Lipoxygenase inhibitors attenuate growth of human pancreatic cancer xenografts and induce apoptosis through the mitochondrial pathway. *Mol Cancer Ther* 1, 929–935 (2002).

10. Hotz, H. G. *et al.* Evaluation of Vascular Endothelial Growth Factor Blockade and Matrix Metalloproteinase Inhibition as a Combination Therapy for Experimental Human Pancreatic Cancer. *Journal of Gastrointestinal Surgery.* 7, 2, 220-227 (2003).

11. Hwang, R. F. *et al.* Inhibition of platelet-derived growth factor receptor phosphorylation by STI571 (Gleevec) reduces growth and metastasis of human pancreatic carcinoma in an orthotopic nude mouse model. *Clin Cancer Res* 9, 6534–6544 (2003).

12. Stephan, S. *et al.* Effect of rapamycin alone and in combination with antiangiogenesis therapy in an orthotopic model of human pancreatic cancer. *Clin Cancer Res* 10, 6993–7000 (2004).

13. Yezhelyev, M. V *et al.* Inhibition of Src tyrosine kinase as treatment for human pancreatic cancer growing orthotopically in nude mice. *Clinical Cancer Research* 10, 8028–8036 (2004).

14. Gumireddy, K. *et al.* ON01910, a non-ATP-competitive small molecule inhibitor of Plk1, is a potent anticancer agent. *Cancer Cell* 7, 275–286 (2005).

15. Kazuno, H. *et al.* Possible antitumor activity of 1-(3-C-ethynyl-β-D-ribo-pentofuranosyl)cytosine (ECyd, TAS-106) against an established gemcitabine (dFdCyd)-resistant human pancreatic cancer cell line. *Cancer Sci* 96, 295–302 (2005).

16. Brezak, M. C. *et al.* Inhibition of human tumor cell growth in vivo by an orally bioavailable inhibitor of CDC25 phosphatases. *Mol Cancer Ther* 4, 1378–1387 (2005).

17. Yau, C. Y. F., Wheeler, J. J., Sutton, K. L. & Hedley, D. W. Inhibition of integrin-linked kinase by QLT0254 inhibits Akt-dependent pathways and is growth inhibitory in orthotopic primary pancreatic cancer xenografts. *Cancer Res* 65, 1497–1504 (2005).

18. Dehn, D. L. *et al.* 5-Methoxy-1,2-dimethyl-3-[(4-nitrophenoxy)methyl] indole-4,7-dione, a mechanism-based inhibitor of NAD(P)H:quinone oxidoreductase 1, exhibits activity against human pancreatic cancer in vitro and in vivo. *Mol Cancer Ther* 5, 1702–1709 (2006).

19. Durkin, A. J. *et al.* EGF Receptor Antagonism Improves Survival in a Murine Model of Pancreatic Adenocarcinoma. *Journal of Surgical Research* 135, 195–201 (2006).

20. Bianco, C. *et al.* Synergistic antitumor activity of ZD6474, an inhibitor of vascular endothelial growth factor receptor and epidermal growth factor receptor signaling, with gemcitabine and ionizing radiation against pancreatic cancer. *Clinical Cancer Research* 12, 7099–7107 (2006).

21. Pino, M. S. *et al.* Transforming growth factor α expression drives constitutive epidermal growth factor receptor pathway activation and sensitivity to gefitinib (Iressa) in human pancreatic cancer cell lines. *Cancer Res* 66, 3802–3812 (2006).

22. Jimeno, A. *et al.* Dual mitogen-activated protein kinase and epidermal growth factor receptor inhibition in biliary and pancreatic cancer. *Mol Cancer Ther* 6, 1079–1088 (2007).

23. Karikari, C. A. *et al.* Targeting the apoptotic machinery in pancreatic cancers using small-molecule antagonists of the X-linked inhibitor of apoptosis protein. *Mol Cancer Ther* 6, 957–966 (2007).

24. Gaspar, N. J. *et al.* Inhibition of transforming growth factor β signaling reduces pancreatic adenocarcinoma growth and invasiveness. *Mol Pharmacol* 72, 152–161 (2007).

25. Kobayashi, S. *et al.* Rapamycin, a specific inhibitor of the mammalian target of rapamycin, suppresses lymphangiogenesis and lymphatic metastasis. *Cancer Sci* 98, 726–733 (2007).

26. Ischenko, I. *et al.* Effect of Src kinase inhibition on metastasis and tumor angiogenesis in human pancreatic cancer. *Angiogenesis* 10, 167–182 (2007).

27. Büchler, P. *et al.* Target therapy using a small molecule inhibitor against angiogenic receptors in pancreatic cancer. *Neoplasia* 9, 119–127 (2007).

28. El Fitori, J. *et al.* PKC412 small-molecule tyrosine kinase inhibitor: Single-compound therapy for pancreatic cancer. *Cancer* 110, 1457–1468 (2007).

29. Medicherla, S. *et al.* Antitumor activity of TGF-beta inhibitor is dependent on the microenvironment. *Anticancer Res* 27, 4149–4157 (2007).

30. Kawasaki, D. *et al.* Effect of Z-360, a novel orally active CCK-2/gastrin receptor antagonist on tumor growth in human pancreatic adenocarcinoma cell lines in vivo and mode of action determinations in vitro. *Cancer Chemother Pharmacol* 61, 883–892 (2007).

31. Rubio-Viqueira, B. *et al.* Optimizing the development of targeted agents in pancreatic cancer: tumor fine-needle aspiration biopsy as a platform for novel prospective ex vivo drug sensitivity assays. *Mol Cancer Ther* 6, 515–523 (2007).

32. Scholz, A. *et al.* The oral multitarget tumour growth inhibitor, ZK 304709, inhibits growth of pancreatic neuroendocrine tumours in an orthotopic mouse model. *Gut* 58, 261–270 (2008).

33. Feldmann, G. *et al.* An orally bioavailable small-molecule inhibitor of Hedgehog signaling inhibits tumor initiation and metastasis in pancreatic cancer. *Mol Cancer Ther* 7, 2725–2735 (2008).

34. Kapischke, M. *et al.* Characterisation of a novel matrix metalloproteinase inhibitor on pancreatic adenocarcinoma cells in vitro and in an orthotopic pancreatic cancer model in vivo. *Int J Oncol* 32, 273–282 (2008).

35. Roberts, W. G. *et al.* Antitumor activity and pharmacology of a selective focal adhesion kinase inhibitor, PF-562,271. *Cancer Res* 68, 1935–1944 (2008).

36. Haefner, M. *et al.* Experimental treatment of pancreatic cancer with two novel histone deacetylase inhibitors. *World J Gastroenterol* 14, 3681–3692 (2008).

37. Moser, C. *et al.* Inhibition of insulin-like growth factor-I receptor (IGF-IR) using NVP-AEW541, a small molecule kinase inhibitor, reduces orthotopic pancreatic cancer growth and angiogenesis. *Eur J Cancer* 44, 1577–1586 (2008).

38. Lu, Y. Y., Jing, D. D., Xu, M., Wu, K. & Wang, X. P. Anti-tumor activity of erlotinib in the BxPC-3 pancreatic cancer cell line. *World J Gastroenterol* 14, 5403–5411 (2008).

39. Song, D. *et al.* Antitumor activity and molecular effects of the novel heat shock protein 90 inhibitor, IPI-504, in pancreatic cancer. *Mol Cancer Ther* 7, 3275–3284 (2008).

40. Nakajima, A. *et al.* Inhibition of peroxisome proliferator-activated receptor γ activity suppresses pancreatic cancer cell motility. *Cancer Sci* 99, 1892–1900 (2008).

41. Lang, S. A. *et al.* Dual targeting of Raf and VEGF receptor 2 reduces growth and metastasis of pancreatic cancer through direct effects on tumor cells, endothelial cells, and pericytes. *Mol Cancer Ther* 7, 3509–3518 (2008).

42. Azmi, A. S. *et al.* Critical role of prostate apoptosis response-4 in determining the sensitivity of pancreatic cancer cells to small-molecule inhibitor-induced apoptosis. *Mol Cancer Ther* 7, 2884–2893 (2008).

43. Sloss, C. M. *et al.* Proteasome inhibition activates epidermal growth factor receptor (EGFR) and EGFR-lndependent mitogenic kinase signaling pathways in pancreatic cancer cells. *Clinical Cancer Research* 14, 5116–5123 (2008).

44. Cao, X. *et al.* Synergistic antipancreatic tumor effect by simultaneously targeting hypoxic cancer cells with HSP90 inhibitor and glycolysis inhibitor. *Clinical Cancer Research* 14, 1831–1839 (2008).

45. Ochiai, T. *et al.* Inhibition of IkappaB kinase beta restrains oncogenic proliferation of pancreatic cancer cells. *J Med Dent Sci* 55, 49–59 (2008).

46. Zhang, Y. *et al.* IκBα kinase inhibitor IKI-1 conferred tumor necrosis factor α sensitivity to pancreatic cancer cells and a xenograft tumor model. *Cancer Res* 68, 9519–9524 (2008).

47. Schwartz, A. L. *et al.* Phenylmethimazole decreases toll-like receptor 3 and noncanonical Wnt5a expression in pancreatic cancer and melanoma together with tumor cell growth and migration. *Clinical Cancer Research* 15, 4114–4122 (2009).

48. Lu, J. *et al.* In vivo antitumor effect of a novel inhibitor of protein geranylgeranyltransferase-I. *Mol Cancer Ther* 8, 1218–1226 (2009).

49. Lu, H., Klein, R. S. & Schwartz, E. L. Antiangiogenic and antitumor activity of 6-(2-aminoethyl)amino-5- chlorouracil, a novel small-molecule inhibitor of thymidine phosphorylase, in combination with the vascular endothelial growth factor-trap. *Clinical Cancer Research* 15, 5136–5144 (2009).

50. Chang, Q., Chen, E. & Hedley, D. W. Effects of combined inhibition of MeK and mTOR on downstream signaling and tumor growth in pancreatic cancer xenograft models. *Cancer Biol Ther* 8, 1893–1901 (2009).

51. Yan, C. *et al.* Potent activity of indolequinones against human pancreatic cancer: Identification of thioredoxin reductase as a potential target. *Mol Pharmacol* 76, 163–172 (2009).

52. Komoto, M. *et al.* In vitro and in vivo evidence that a combination of lapatinib plus S-1 is a promising treatment for pancreatic cancer. *Cancer Sci* 101, 468–473 (2009).

53. Hochwald, S. N. *et al.* A novel small molecule inhibitor of FAK decreases growth of human pancreatic cancer. *Cell Cycle* 8, 2435–2443 (2009).

54. McLaughlin, J. *et al.* Preclinical characterization of Aurora kinase inhibitor R763/AS703569 identified through an image-based phenotypic screen. *J Cancer Res Clin Oncol* 136, 99–113 (2010).

55. Furugaki, K., Iwai, T., Kondoh, K., Moriya, Y. & Mori, K. Antitumor activity of erlotinib in combination with gemcitabine in in vitro and in vivo models of KRAS-mutated pancreatic cancers. *Oncol Lett* 1, 231–235 (2010).

56. Nagaraj, N. S., Smith, J. J., Revetta, F., Washington, M. K. & Merchant, N. B. Targeted inhibition of Src kinase signaling attenuates pancreatic tumorigenesis. *Mol Cancer Ther* 9, 2322–2332 (2010).

57. Koblish, H. K. *et al.* Hydroxyamidine inhibitors of indoleamine-2,3-dioxygenase potently suppress systemic tryptophan catabolism and the growth of IDO-expressing tumors. *Mol Cancer Ther* 9, 489–498 (2010).

58. Jaganathan, S., Yue, P. & Turkson, J. Enhanced sensitivity of pancreatic cancer cells to concurrent inhibition of aberrant signal transducer and activator of transcription 3 and epidermal growth factor receptor or Src. *J Pharmacol Exp Ther* 333, 373–381 (2010).

59. Zhang, Y.-W. *et al.* Why two are better than one: Suppression of carcinoma cell growth in vitro and in human HGF/SF transgenic SCID by inhibitors of MET (SGX523) and EGFR (erlotinib). *Cancer Res* 70, (2010).

60. Harikumar, K. B. *et al.* A novel small-molecule inhibitor of protein kinase D blocks pancreatic cancer growth in vitro and in vivo. *Mol Cancer Ther* 9, 1136–1146 (2010).

61. Zheng, D. *et al.* A novel strategy to inhibit FAK and IGF-1R decreases growth of pancreatic cancer xenografts. *Mol Carcinog* 49, 200–209 (2010).

62. Cai, X. *et al.* Discovery of 7-(4-(3-Ethynylphenylamino)-7-methoxyquinazolin-6-yloxy)-N- hydroxyheptanamide (CUDC-101) as a potent multi-acting HDAC, EGFR, and HER2 inhibitor for the treatment of cancer. *J Med Chem* 53, 2000–2009 (2010).

63. Feldmann, G. *et al.* Cyclin-dependent kinase inhibitor dinaciclib (SCH727965) inhibits pancreatic cancer growth and progression in murine xenograft models. *Cancer Biol Ther* 12, 598–609 (2011).

64. Fendrich, V. *et al.* Hedgehog inhibition with the orally bioavailable smo antagonist LDE225 represses tumor growth and prolongs survival in a transgenic mouse model of islet cell neoplasms. *Ann Surg* 254, 818–823 (2011).

65. Strand, M. F. *et al.* A novel synthetic smoothened antagonist transiently inhibits pancreatic adenocarcinoma xenografts in a mouse model. *PLoS One* 6, (2011).

66. Taeger, J. *et al.* Targeting FGFR/PDGFR/VEGFR impairs tumor growth, angiogenesis, and metastasis by effects on tumor cells, endothelial cells, and pericytes in pancreatic cancer. *Mol Cancer Ther* 10, 2157–2167 (2011).

67. Nagaraj, N. S., Washington, M. K. & Merchant, N. B. Combined blockade of Src kinase and epidermal growth factor receptor with gemcitabine overcomes STAT3-mediated resistance of inhibition of pancreatic tumor growth. *Clin Cancer Res* 17, 483–493 (2011).

68. Mirzoeva, O. K. *et al.* Autophagy suppression promotes apoptotic cell death in response to inhibition of the PI3K-mTOR pathway in pancreatic adenocarcinoma. *J Mol Med* 89, 877–889 (2011).

69. Jasinski, P. *et al.* MT477 acts in tumor cells as an AURKA inhibitor and strongly induces NRF-2 signaling. *Anticancer Res* 31, 1181–1187 (2011).

70. Beljanski, V., Knaak, C., Zhuang, Y. & Smith, C. D. Combined anticancer effects of sphingosine kinase inhibitors and sorafenib. *Invest New Drugs* 29, 1132–1142 (2011).

71. Fujisawa, T., Joshi, B. H. & Puri, R. K. Histone modification enhances the effectiveness of IL-13 receptor targeted immunotoxin in murine models of human pancreatic cancer. *J Transl Med* 9, 37 (2011).

72. Awasthi, N., Zhang, C., Ruan, W., Schwarz, M. A. & Schwarz, R. E. BMS-754807, a small-molecule inhibitor of insulin-like growth factor-1 receptor/insulin receptor, enhances gemcitabine response in pancreatic cancer. *Mol Cancer Ther* 11, 2644–2653 (2012).

73. Dovzhanskiy, D. I. *et al.* Experimental in vivo and in vitro treatment with a new histone deacetylase inhibitor belinostat inhibits the growth of pancreatic cancer. *BMC Cancer* 12, (2012).

74. Hwang, R. F. *et al.* Inhibition of the hedgehog pathway targets the tumor-associated stroma in pancreatic cancer. *Molecular Cancer Research* 10, 1147–1157 (2012).

75. Fraedrich, K. *et al.* TarGeting aurora kinases with danusertib (PHA-739358) inhibits growth of liver metastases from gastroenteropancreatic neuroendocrine tumors in an orthotopic xenograft model. *Clinical Cancer Research* 18, 4621–4632 (2012).

76. Glaser, K. B. *et al.* Preclinical characterization of ABT-348, a kinase inhibitor targeting the Aurora, vascular endothelial growth factor receptor/platelet-derived growth factor receptor, and Src kinase families. *Journal of Pharmacology and Experimental Therapeutics* 343, 617–627 (2012).

77. Moser, C. *et al.* Targeting HSP90 by the novel inhibitor NVP-AUY922 reduces growth and angiogenesis of pancreatic cancer. *Anticancer Res* 32, 2551–2561 (2012).

78. Komachi, M. *et al.* Orally active lysophosphatidic acid receptor antagonist attenuates pancreatic cancer invasion and metastasis in vivo. *Cancer Sci* 103, 1099–1104 (2012).

79. Fendrich, V. *et al.* Sorafenib inhibits tumor growth and improves survival in a transgenic mouse model of pancreatic islet cell tumors. *ScientificWorldJournal* 2012, 529151 (2012).

80. Hofmann, I. *et al.* K-RAS Mutant Pancreatic Tumors Show Higher Sensitivity to MEK than to PI3K Inhibition In Vivo. *PLoS One* 7, (2012).

81. Awasthi, N., Zhang, C., Ruan, W., Schwarz, M. A. & Schwarz, R. E. Evaluation of poly-mechanistic antiangiogenic combinations to enhance cytotoxic therapy response in pancreatic cancer. *PLoS One* 7, e38477–e38477 (2012).

82. Moser, C. *et al.* Oncogenic MST1R activity in pancreatic and gastric cancer represents a valid target of HSP90 inhibitors. *Anticancer Res* 32, 427–437 (2012).

83. Bladt, F. *et al.* EMD 1214063 and EMD 1204831 constitute a new class of potent and highly selective c-Met inhibitors. *Clinical Cancer Research* 19, 2941–2951 (2013).

84. Gomes, E. G., Connelly, S. F. & Summy, J. M. Targeting the yin and the yang: Combined inhibition of the tyrosine kinase c-Src and the tyrosine phosphatase SHP-2 disrupts pancreatic cancer signaling and biology in vitro and tumor formation in vivo. *Pancreas* 42, 795–806 (2013).

85. Yabuuchi, S. *et al.* Notch signaling pathway targeted therapy suppresses tumor progression and metastatic spread in pancreatic cancer. *Cancer Lett* 335, 41–51 (2013).

86. Abulwerdi, F. *et al.* A novel small-molecule inhibitor of Mcl-1 blocks pancreatic cancer growth in vitro and in vivo. *Mol Cancer Ther* 13, 565–575 (2013).

87. Kurenova, E. *et al.* The FAK scaffold inhibitor C4 disrupts FAK-VEGFR-3 signaling and inhibits pancreatic cancer growth. *Oncotarget* 4, 1632–1646 (2013).

88. Gong, D. J., Zhang, J. M., Yu, M., Zhuang, B. & Guo, Q. Q. Inhibition of SIRTI combined with gemcitabine therapy for pancreatic carcinoma. *Clin Interv Aging* 8, 889–897 (2013).

89. Ucar, D. A. *et al.* *Inhibiting the interaction of cMET and IGF-1R with FAK effectively reduces growth of pancreatic cancer cells in vitro and in vivo NIH Public Access*. *Anticancer Agents Med Chem* 13, (2013).

90. Mackenzie, G. G. *et al.* A novel Ras inhibitor (MDC-1016) reduces human pancreatic tumor growth in mice. *Neoplasia* 15, 1184–1195 (2013).

91. Zhong, H. *et al.* Synergistic Effects of Concurrent Blockade of PI3K and MEK Pathways in Pancreatic Cancer Preclinical Models. *PLoS One* 8, (2013).

92. Zimmermann, G. *et al.* Small molecule inhibition of the KRAS-PDEδ interaction impairs oncogenic KRAS signalling. *Nature* 497, 638–642 (2013).

93. Palagani, V. *et al.* Combined inhibition of Notch and JAK/STAT is superior to monotherapies and impairs pancreatic cancer progression. *Carcinogenesis* 35, 859–866 (2014).

94. Peluso, M. O. *et al.* Impact of the smoothened inhibitor, IPI-926, on smoothened ciliary localization and hedgehog pathway activity. *PLoS One* 9, (2014).

95. Fendrich, V. *et al.* Inhibition of heat shock protein 90 with AUY922 represses tumor growth in a transgenic mouse model of islet cell neoplasms. *Neuroendocrinology* 100, 300–309 (2014).

96. Abel, E. V. *et al.* The notch pathway is important in maintaining the cancer stem cell population in pancreatic cancer. *PLoS One* 9, (2014).

97. Lucero-Acuña, A. *et al.* Nanoparticle delivery of an akt/pdk1 inhibitor improves the therapeutic effect in pancreatic cancer. *Int J Nanomedicine* 9, 5653–5665 (2014).

98. Hayman, T. J. *et al.* The ATP-competitive mTOR inhibitor INK128 enhances in vitro and in vivo radiosensitivity of pancreatic carcinoma cells. *Clinical Cancer Research* 20, 110–119 (2014).

99. Zhang, H. *et al.* Enhanced FGFR signalling predisposes pancreatic cancer to the effect of a potent FGFR inhibitor in preclinical models. *Br J Cancer* 110, 320–329 (2014).

100. Chien, W. *et al.* Selective inhibition of unfolded protein response induces apoptosis in pancreatic cancer cells. *Oncotarget* 5, 4881–4894 (2014).

101. Valentino, J. D. *et al.* Cotargeting the PI3K and RAS pathways for the treatment of neuroendocrine tumors. *Clinical Cancer Research* 20, 1212–1222 (2014).

102. Wong, M. H. *et al.* Cotargeting of epidermal growth factor receptor and PI3K overcomes PI3K-Akt oncogenic dependence in pancreatic ductal adenocarcinoma. *Clinical Cancer Research* 20, 4047–4058 (2014).

103. Bai, X. *et al.* Inhibition of protein phosphatase 2A sensitizes pancreatic cancer to chemotherapy by increasing drug perfusion via HIF-1α-VEGF mediated angiogenesis. *Cancer Lett* 355, 281–287 (2014).

104. Heilmann, A. M. *et al.* CDK4/6 and IGF1 receptor inhibitors synergize to suppress the growth of p16INK4A -Deficient Pancreatic Cancers. *Cancer Res* 74, 3947–3958 (2014).

105. Chien, W. *et al.* Growth inhibition of pancreatic cancer cells by histone deacetylase inhibitor belinostat through suppression of multiple pathways including HIF, NFkB, and mTOR signaling in vitro and in vivo. *Mol Carcinog* 53, 722–735 (2014).

106. Gogate, P. N. *et al.* Targeting the C-terminal focal adhesion kinase scaffold in pancreatic cancer. *Cancer Lett* 353, 281–289 (2014).

107. Zhong, Y. *et al.* Functional p38 MAPK identified by biomarker profiling of pancreatic cancer restrains growth through JNK inhibition and correlates with improved survival. *Clinical Cancer Research* 20, 6200–6211 (2014).

108. Almahariq, M. *et al.* Pharmacological inhibition and genetic knockdown of exchange protein directly activated by cAMP 1 reduce pancreatic cancer metastasis in vivo. *Mol Pharmacol* 87, 142–149 (2014).

109. Wu, J. *et al.* Matrix metalloproteinase inhibitor MMI-166 suppresses the growth of SW1990 human pancreatic cancer cells. *Curr Signal Transduct Ther* 9, 93–100 (2014).

110. Weissmueller, S. *et al.* Mutant p53 drives pancreatic cancer metastasis through cell-autonomous PDGF receptor β signaling. *Cell* 157, 382–394 (2014).

111. Hamilton, G. *et al.* AKT regulates NPM dependent ARF localization and p53mut stability in tumors. *Oncotarget* 5, 6142–6167 (2014).

112. Deng, X. & Friedman, E. Mirk Kinase inhibition blocks the in vivo growth of pancreatic cancer cells. *Genes Cancer* 5, 337–347 (2014).

113. Mace, T. A. *et al.* Single agent BMS-911543 Jak2 inhibitor has distinct inhibitory effects on STAT5 signaling in genetically engineered mice with pancreatic cancer. *Oncotarget* 6, 44509–44522 (2015).

114. Zhao, X. *et al.* Small molecule inhibitor YM155-mediated activation of death receptor 5 is crucial for chemotherapy-induced apoptosis in pancreatic carcinoma. *Mol Cancer Ther* 14, 80–89 (2015).

115. Kazim, S. *et al.* Selective nuclear export inhibitor KPT-330 enhances the antitumor activity of gemcitabine in human pancreatic cancer. *Mol Cancer Ther* 14, 1570–1581 (2015).

116. Minjie, S., Defei, H., Zhimin, H., Weiding, W. & Yuhua, Z. Targeting pancreatic cancer cells by a novel hydroxamate-based histone deacetylase (HDAC) inhibitor ST-3595. *Tumor Biology* 36, 9015–9022 (2015).

117. Hari, Y., Harashima, N., Tajima, Y. & Harada, M. Bcl-xL inhibition by molecular-targeting drugs sensitizes human pancreatic cancer cells to TRAIL. *Oncotarget* 6, 41902–41915 (2015).

118. Takano, H. *et al.* Inhibition of Eph receptor A4 by 2,5-dimethylpyrrolyl benzoic acid suppresses human pancreatic cancer growing orthotopically in nude mice. *Oncotarget* 6, 41063–41076 (2015).

119. Brandes, F. *et al.* Targeting cMET with INC280 impairs tumour growth and improves efficacy of gemcitabine in a pancreatic cancer model. *BMC Cancer* 15, (2015).

120. Gao, X. *et al.* CDDO-Me inhibits tumor growth and prevents recurrence of pancreatic ductal adenocarcinoma. *Int J Oncol* 47, 2100–2106 (2015).

121. Awasthi, N., Hinz, S., Brekken, R. A., Schwarz, M. A. & Schwarz, R. E. Nintedanib, a triple angiokinase inhibitor, enhances cytotoxic therapy response in pancreatic cancer. *Cancer Lett* 358, 59–66 (2015).

122. Wei, F. *et al.* mTOR inhibition induces EGFR feedback activation in association with its resistance to human pancreatic cancer. *Int J Mol Sci* 16, 3267–3282 (2015).

123. Li, Y. *et al.* The molecular mechanisms of a novel multi-kinase inhibitor ZLJ33 in suppressing pancreatic cancer growth. *Cancer Lett* 356, 392–403 (2015).

124. Nagathihalli, N. S. *et al.* Signal Transducer and Activator of Transcription 3, Mediated Remodeling of the Tumor Microenvironment Results in Enhanced Tumor Drug Delivery in a Mouse Model of Pancreatic Cancer. *Gastroenterology* 149, 1932-1943.e9 (2015).

125. Vena, F. *et al.* The MEK1/2 Inhibitor Pimasertib Enhances Gemcitabine Efficacy in Pancreatic Cancer Models by Altering Ribonucleotide Reductase Subunit-1 (RRM1). *Clinical Cancer Research* 21, 5563–5577 (2015).

126. François, R. A. *et al.* Targeting Focal Adhesion Kinase and Resistance to mTOR Inhibition in Pancreatic Neuroendocrine Tumors. *J Natl Cancer Inst* 107, (2015).

127. Moen, I. *et al.* Anti-metastatic action of FAK inhibitor OXA-11 in combination with VEGFR-2 signaling blockade in pancreatic neuroendocrine tumors. *Clin Exp Metastasis* 32, 799–817 (2015).

128. Awasthi, N. *et al.* Augmentation of response to nab-paclitaxel by inhibition of insulin-like growth factor (IGF) signaling in preclinical pancreatic cancer models. *Oncotarget* 7, 46988–47001 (2016).

129. Yamamoto, K. *et al.* Stromal remodeling by the BET bromodomain inhibitor JQ1 suppresses the progression of human pancreatic cancer. *Oncotarget* 7, 61469–61484 (2016).

130. Recio-Boiles, A. *et al.* JNK pathway inhibition selectively primes pancreatic cancer stem cells to TRAIL-induced apoptosis without affecting the physiology of normal tissue resident stem cells. *Oncotarget* 7, 9890–9906 (2016).

131. Jiang, Y. *et al.* The small-molecule IAP antagonist AT406 inhibits pancreatic cancer cells in vitro and in vivo. *Biochem Biophys Res Commun* 478, 293–299 (2016).

132. Yeo, D. *et al.* FRAX597, a PAK1 inhibitor, synergistically reduces pancreatic cancer growth when combined with gemcitabine. *BMC Cancer* 16, (2016).

133. Arpin, C. C. *et al.* Applying small molecule signal transducer and activator of transcription-3 (STAT3) protein inhibitors as pancreatic cancer therapeutics. *Mol Cancer Ther* 15, 794–805 (2016).

134. Barnard, D. *et al.* LY2603618, a selective CHK1 inhibitor, enhances the anti-tumor effect of gemcitabine in xenograft tumor models. *Invest New Drugs* 34, 49–60 (2016).

135. Henderson, S. E. *et al.* Suppression of Tumor Growth and Muscle Wasting in a Transgenic Mouse Model of Pancreatic Cancer by the Novel Histone Deacetylase Inhibitor AR-42. *Neoplasia (United States)* 18, 765–774 (2016).

136. Lee, H. S. *et al.* A novel HDAC inhibitor, CG200745, inhibits pancreatic cancer cell growth and overcomes gemcitabine resistance. *Sci Rep* 7, 41615 (2017).

137. Espindola-Netto, J. M. *et al.* Preclinical efficacy of the novel competitive NAMPT inhibitor STF-118804 in pancreatic cancer. *Oncotarget* 8, 85054–85067 (2017).

138. Langdon, C. G. *et al.* Combinatorial screening of pancreatic adenocarcinoma reveals sensitivity to drug combinations including bromodomain inhibitor plus neddylation inhibitor. *Mol Cancer Ther* 16, 1041–1053 (2017).

139. Li, H. *et al.* Inhibition of Neddylation Modification Sensitizes Pancreatic Cancer Cells to Gemcitabine. *Neoplasia (United States)* 19, 509–518 (2017).

140. Mathison, A. *et al.* Combined AURKA and H3K9 methyltransferase targeting inhibits cell growth by inducing mitotic catastrophe. *Molecular Cancer Research* 15, 984–997 (2017).

141. Zhou, J. *et al.* Cucurbitacin B and SCH772984 exhibit synergistic anti-pancreatic cancer activities by suppressing EGFR, PI3K/Akt/mTOR, STAT3 and ERK signaling. *Oncotarget* 8, 103167–103181 (2017).

142. Chen, Y. J. *et al.* Novel histone deacetylase inhibitor AR-42 exhibits antitumor activity in pancreatic cancer cells by affecting multiple biochemical pathways. *PLoS One* 12, (2017).

143. Wang, K. *et al.* Inhibition of p21 activated kinase enhances tumour immune response and sensitizes pancreatic cancer to gemcitabine. *Int J Oncol* 52, 261–269 (2017).

144. Zhang, D. *et al.* Constitutive IRAK4 activation underlies poor prognosis and chemoresistance in pancreatic ductal adenocarcinoma. *Clinical Cancer Research* 23, 1748–1759 (2017).

145. Manu, K. A. *et al.* Inhibition of isoprenylcysteine carboxylmethyltransferase induces cell-cycle arrest and apoptosis through p21 and p21-regulated BNIP3 induction in pancreatic cancer. *Mol Cancer Ther* 16, 914–923 (2017).

146. Chen, W. H. *et al.* Discovery of potent 2,4-difluoro-linker poly(ADPribose) polymerase 1 inhibitors with enhanced water solubility and in vivo anticancer efficacy. *Acta Pharmacol Sin* 38, 1521–1532 (2017).

147. Takiguchi, S. *et al.* Crizotinib, a MET inhibitor, prevents peritoneal dissemination in pancreatic cancer. *Int J Oncol* 51, 184–192 (2017).

148. Andrews, F. H. *et al.* Dual-activity PI3K-BRD4 inhibitor for the orthogonal inhibition of MYC to block tumor growth and metastasis. *Proc Natl Acad Sci U S A* 114, E1072–E1080 (2017).

149. Chen, W. *et al.* Discovery, mechanism and metabolism studies of 2,3-difluorophenyl-linker-containing PARP1 inhibitors with enhanced in vivo efficacy for cancer therapy. *Eur J Med Chem* 138, 514–531 (2017).

150. Rath, N. *et al.* Rho kinase inhibition by AT13148 blocks pancreatic ductal adenocarcinoma invasion and tumor growth. *Cancer Res* 78, 3321–3336 (2018).

151. Lewis, C. S. *et al.* mTOR kinase inhibition reduces tissue factor expression and growth of      pancreatic neuroendocrine tumors. *J Thromb Haemost* 17, 169–182 (2018).

152. Bai, E. *et al.* L61H46 shows potent efficacy against human pancreatic cancer through inhibiting STAT3 pathway. *Cancer Manag Res* 10, 565–581 (2018).

153. Pal, A. *et al.* Usp9x Promotes Survival in Human Pancreatic Cancer and Its Inhibition Suppresses Pancreatic Ductal Adenocarcinoma In Vivo Tumor Growth. *Neoplasia (United States)* 20, 152–164 (2018).

154. Jauset, T. *et al.* BET inhibition is an effective approach against KRAS-driven PDAC and NSCLC. *Oncotarget* 9, 18734–18746 (2018).

155. Ludwig, K. F. *et al.* Small-molecule inhibition of Axl targets tumor immune suppression and enhances chemotherapy in pancreatic cancer. *Cancer Res* 78, 246–255 (2018).

156. Chou, A. *et al.* Tailored first-line and second-line CDK4-targeting treatment combinations in mouse models of pancreatic cancer. *Gut* 67, 2142–2155 (2018).

157. Fu, X.-H. *et al.* CUDC-907 displays potent antitumor activity against human pancreatic adenocarcinoma in vitro and in vivo through inhibition of HDAC6 to downregulate c-Myc expression. *Acta Pharmacol Sin* 40, 677–688 (2018).

158. Gitto, S. B. *et al.* Difluoromethylornithine Combined with a Polyamine Transport Inhibitor Is Effective against Gemcitabine Resistant Pancreatic Cancer. *Mol Pharm* 15, 369–376 (2018).

159. Vena, F. *et al.* MEK inhibition leads to BRCA2 downregulation and sensitization to DNA damaging agents in pancreas and ovarian cancer models. *Oncotarget* 9, 11592–11603 (2018).

160. Jiang, H. *et al.* Concurrent HER or PI3K Inhibition Potentiates the Antitumor Effect of the ERK Inhibitor Ulixertinib in Preclinical Pancreatic Cancer Models. *Mol Cancer Ther* 17, 2144–2155 (2018).

161. Xu, Q. *et al.* Inhibition of PTP1B blocks pancreatic cancer progression by targeting the PKM2/AMPK/mTOC1 pathway. *Cell Death Dis* 10, 874 (2019).

162. Zhong, Z. *et al.* PORCN inhibition synergizes with PI3K/mTOR inhibition in Wnt-addicted cancers. *Oncogene* 38, 6662–6677 (2019).

163. Lu, P. *et al.* THZ1 reveals CDK7-dependent transcriptional addictions in pancreatic cancer. *Oncogene* 38, 3932–3945 (2019).

164. Kashyap, V. K. *et al.* Therapeutic efficacy of a novel βiII/βIV-tubulin inhibitor (VERU-111) in pancreatic cancer. *Journal of Experimental and Clinical Cancer Research* 38, (2019).

165. Adamska, A. *et al.* Pharmacological inhibition of ABCC3 slows tumour progression in animal models of pancreatic cancer. *J Exp Clin Cancer Res* 38, 312 (2019).

166. Emmanouilidi, A. *et al.* Preclinical validation of 3-phosphoinositide-dependent protein kinase 1 inhibition in pancreatic cancer. *J Exp Clin Cancer Res* 38, 191 (2019).

167. Miller, A. L. *et al.* The BET inhibitor JQ1 attenuates double-strand break repair and sensitizes models of pancreatic ductal adenocarcinoma to PARP inhibitors. *EBioMedicine* 44, 419–430 (2019).

168. Lu, H. *et al.* SHP2 inhibition overcomes RTK-mediated pathway reactivation in KRAS-mutant tumors treated with MEK inhibitors. *Mol Cancer Ther* 18, 1323–1334 (2019).

169. Dekhne, A. S. *et al.* Novel Pyrrolo[3,2-d]pyrimidine Compounds Target Mitochondrial and Cytosolic One-carbon Metabolism with Broad-spectrum Antitumor Efficacy. *Mol Cancer Ther* 18, 1787–1799 (2019).

170. Chung, S., Vail, P., Witkiewicz, A. K. & Knudsen, E. S. Coordinately targeting cell-cycle checkpoint functions in integrated models of pancreatic cancer. *Clinical Cancer Research* 25, 2290–2304 (2019).

171. Elliott, I. A. *et al.* Lysosome inhibition sensitizes pancreatic cancer to replication stress by aspartate depletion. *Proc Natl Acad Sci U S A* 116, 6842–6847 (2019).

172. Santoro, R. *et al.* Modulating TAK1 Expression Inhibits YAP and TAZ Oncogenic Functions in Pancreatic Cancer. *Mol Cancer Ther* 19, 247–257 (2019).

173. Ogawa, K. *et al.* Aspartate β-hydroxylase promotes pancreatic ductal adenocarcinoma metastasis through activation of SRC signaling pathway. *J Hematol Oncol* 12, (2019).

174. Ponz-Sarvise, M. *et al.* Identification of resistance pathways specific to malignancy using organoid models of pancreatic cancer. *Clinical Cancer Research* 25, 6742–6755 (2019).

175. Su, H. *et al.* N-arylpiperazine-containing compound (C2): An enhancer of sunitinib in the treatment of pancreatic cancer, involving D1DR activation. *Toxicol Appl Pharmacol* 384, 114789 (2019).

176. Brown, W. S. *et al.* Overcoming Adaptive Resistance to KRAS and MEK Inhibitors by Co-targeting mTORC1/2 Complexes in Pancreatic Cancer. *Cell Rep Med* 1, 100131 (2020).

177. Lai, S. W. *et al.* Targeted PARP Inhibition Combined with FGFR1 Blockade is Synthetically Lethal to Malignant Cells in Patients with Pancreatic Cancer. *Cells* 9, (2020).

178. Jin, M. H. *et al.* Therapeutic co-targeting of WEE1 and ATM downregulates PD-L1 expression in pancreatic cancer. *Cancer Res Treat* 52, 149–166 (2020).

179. Konczalla, L. *et al.* Biperiden and mepazine effectively inhibit MALT1 activity and tumor growth in pancreatic cancer. *Int J Cancer* 146, 1618–1630 (2020).

180. Wang, J. *et al.* Identification of a novel PAK1 inhibitor to treat pancreatic cancer. *Acta Pharm Sin B* 10, 603–614 (2020).

181. Dosch, A. R. *et al.* Combined Src/EGFR inhibition targets STAT3 signaling and induces stromal remodeling to improve survival in pancreatic cancer. *Molecular Cancer Research* 18, 623–631 (2020).

182. Kong, R. *et al.* Small Molecule Inhibitor C188-9 Synergistically Enhances the Demethylated Activity of Low-Dose 5-Aza-2′-Deoxycytidine Against Pancreatic Cancer. *Front Oncol* 10, (2020).

183. Vaishnavi, A. *et al.* Inhibition of MEK1/2 Forestalls the Onset of Acquired Resistance to Entrectinib in Multiple Models of NTRK1-Driven Cancer. *Cell Rep* 32, 107994 (2020).

184. Sun, Y. *et al.* The aberrant expression of ADAR1 promotes resistance to BET inhibitors in pancreatic cancer by stabilizing c-Myc. *Am J Cancer Res* 10, 148–163 (2020).

185. Tong, X. *et al.* Identification of a druggable protein–protein interaction site between mutant p53 and its stabilizing chaperone DNAJA1. *Journal of Biological Chemistry* 296, Preprint at https://doi.org/10.1074/jbc.ra120.014749 (2020)

186. Grbovic-Huezo, O. *et al.* Unbiased in vivo preclinical evaluation of anticancer drugs identifies effective therapy for the treatment of pancreatic adenocarcinoma. *Proc Natl Acad Sci U S A* 117, 30670–30678 (2020).

187. Wang, Z. *et al.* SETD5-Coordinated Chromatin Reprogramming Regulates Adaptive Resistance to Targeted Pancreatic Cancer Therapy. *Cancer Cell* 37, 834-849.e13 (2020).

188. Caston, R. A. *et al.* Combined inhibition of Ref-1 and STAT3 leads to synergistic tumour inhibition in multiple cancers using 3D and in vivo tumour co-culture models. *J Cell Mol Med* 25, 784–800 (2020).

189. Yang, Y. *et al.* Design, synthesis and biological evaluation of imidazolopyridone derivatives as novel BRD4 inhibitors. *Bioorg Med Chem* 29, (2021).

190. Ren, D. *et al.* SGLT2 promotes pancreatic cancer progression by activating the Hippo signaling pathway via the hnRNPK-YAP1 axis. *Cancer Lett* 519, 277–288 (2021).

191. Wang, Y. *et al.* Inhibition of bone morphogenetic protein receptor 2 suppresses pancreatic ductal adenocarcinoma growth by regulating GRB2/ PI3K/AKT axis. *Ann Transl Med* 9, 557–557 (2021).

192. Le Large, T. Y. S. *et al.* Focal adhesion kinase inhibition synergizes with nab-paclitaxel to target pancreatic ductal adenocarcinoma. *Journal of Experimental and Clinical Cancer Research* 40, (2021).

193. Kattan, W. E. *et al.* Components of the phosphatidylserine endoplasmic reticulum to plasma membrane transport mechanism as targets for KRAS inhibition in pancreatic cancer. *Proc Natl Acad Sci U S A* 118, (2021).

194. Liu, S. *et al.* Antitumor activity and mechanism of resistance of the novel HDAC and PI3K dual inhibitor CUDC-907 in pancreatic cancer. *Cancer Chemother Pharmacol* 87, 415–423 (2021).

195. Nakkina, S. P. *et al.* Dfmo improves survival and increases immune cell infiltration in association with myc downregulation in the pancreatic tumor microenvironment. *Int J Mol Sci* 22, (2021).

196. Hartman, S. J. *et al.* WEE1 Inhibition in Combination With Targeted Agents and Standard Chemotherapy in Preclinical Models of Pancreatic Ductal Adenocarcinoma. *Front Oncol* 11, (2021).

197. April-Monn, S. L. *et al.* Ezh2 inhibition as new epigenetic treatment option for pancreatic neuroendocrine neoplasms (Pannens). *Cancers (Basel)* 13, (2021).

198. Zhao, Y. *et al.* Targeted intervention of eIF4A1 inhibits EMT and metastasis of pancreatic cancer cells via c-MYC/miR-9 signaling. *Cancer Cell Int* 21, (2021).

199. Sun, D. *et al.* Evaluation of the small-molecule BRD4 degrader CFT-2718 in small-cell lung cancer and pancreatic cancer models. *Mol Cancer Ther* 20, 1367–1377 (2021).

200. Dubiella, C. *et al.* Sulfopin is a covalent inhibitor of Pin1 that blocks Myc-driven tumors in vivo. *Nat Chem Biol* 17, 954–963 (2021).

201. Ding, Z. *et al.* Cholesterol biosynthesis inhibitor RO 48‑8071 inhibits pancreatic ductal adenocarcinoma cell viability by deactivating the JNK and ERK/MAPK signaling pathway. *Mol Med Rep* 24, (2021).

202. Qian, W. *et al.* The EGFR-HSF1 axis accelerates the tumorigenesis of pancreatic cancer. *Journal of Experimental and Clinical Cancer Research* 40, (2021).

203. Zhang, Z. *et al.* Kaempferol potentiates the sensitivity of pancreatic cancer cells to erlotinib via inhibition of the PI3K/AKT signaling pathway and epidermal growth factor receptor. *Inflammopharmacology* 29, 1587–1601 (2021).

204. Doffo, J. *et al.* NOXA expression drives synthetic lethality to RUNX1 inhibition in pancreatic cancer. *Proc Natl Acad Sci U S A* 119, (2022).

205. He, J. *et al.* A Novel Small Molecular Prostaglandin Receptor EP4 Antagonist, L001, Suppresses Pancreatic Cancer Metastasis. *Molecules* 27, (2022).

206. Xu, J. *et al.* Pevonedistat Suppresses Pancreatic Cancer Growth via Inactivation of the Neddylation Pathway. *Front Oncol* 12, (2022).

207. Principe, D. R. *et al.* XP-524 is a dual-BET/EP300 inhibitor that represses oncogenic KRAS and potentiates immune checkpoint inhibition in pancreatic cancer. *Proc Natl Acad Sci U S A* 119, (2022).

208. Xue, D. *et al.* Multiparameter Optimization of Oxidative Phosphorylation Inhibitors for the Treatment of Pancreatic Cancer. *J Med Chem* 65, 3404–3419 (2022).

209. Qi, S. *et al.* Targeting E2 ubiquitin-conjugating enzyme UbcH5c by small molecule inhibitor suppresses pancreatic cancer growth and metastasis. *Mol Cancer* 21, (2022).

210. Wu, S. Q. *et al.* FDI-6 and olaparib synergistically inhibit the growth of pancreatic cancer by repressing BUB1, BRCA1 and CDC25A signaling pathways. *Pharmacol Res* 175, (2022).

211. Kumar, S. *et al.* Targeting pancreatic cancer by TAK-981: a SUMOylation inhibitor that activates the immune system and blocks cancer cell cycle progression in a preclinical model. *Gut* 71, 2266–2283 (2022).

212. Bachmann, M. *et al.* Pharmacological targeting of the mitochondrial calcium-dependent potassium channel KCa3.1 triggers cell death and reduces tumor growth and metastasis in vivo. *Cell Death Dis* 13, (2022).

213. Frank, K. J. *et al.* Extensive preclinical validation of combined RMC-4550 and LY3214996 supports clinical investigation for KRAS mutant pancreatic cancer. *Cell Rep Med* 3, (2022).

214. Parisotto, M. *et al.* The NAMPT Inhibitor FK866 Increases Metformin Sensitivity in Pancreatic Cancer Cells. *Cancers (Basel)* 14, (2022).

215. Li, B., Feng, Y., Hou, Q., Fu, Y. & Luo, Y. Antigen Peptide Transporter 1 (TAP1) Promotes Resistance to MEK Inhibitors in Pancreatic Cancers. *Int J Mol Sci* 23, (2022).

216. Hoque, M. M., Iida, Y., Kotani, H., Kartika, I. D. & Harada, M. Hydroxychloroquine Promotes Bcl-xL Inhibition-induced Apoptosis in BxPC-3 Human Pancreatic Cancer Cells. *Anticancer Res* 42, 3495–3506 (2022).

217. Ha, D. P. *et al.* Targeting GRP78 suppresses oncogenic KRAS protein expression and reduces viability of cancer cells bearing various KRAS mutations. *Neoplasia (United States)* 33, (2022).

218. Quiñonero, F. *et al.* PARP1 inhibition by Olaparib reduces the lethality of pancreatic cancer cells and increases their sensitivity to Gemcitabine. *Biomedicine and Pharmacotherapy* 155, (2022).

219. Guardado Rivas, M. O. *et al.* Evidence for a novel, effective approach to targeting carcinoma catabolism exploiting the first-in-class, anti-cancer mitochondrial drug, CPI-613. *PLoS One* 17, (2022).

220. Cheng, F. *et al.* Trefoil factor 3 promotes pancreatic carcinoma progression via WNT pathway activation mediated by enhanced WNT ligand expression. *Cell Death Dis* 13, (2022).

221. Kumstel, S. *et al.* Targeting pancreatic cancer with combinatorial treatment of CPI-613 and inhibitors of lactate metabolism. *PLoS One* 17, (2022).

222. Zhang, C. *et al.* Small molecule inhibitor against onco-mucins disrupts Src/FosL1 axis to enhance gemcitabine efficacy in pancreatic ductal adenocarcinoma. *Cancer Lett* 551, (2022).

223. Pacchiana, R. *et al.* 3-Bromo-Isoxazoline Derivatives Inhibit GAPDH Enzyme in PDAC Cells Triggering Autophagy and Apoptotic Cell Death. *Cancers (Basel)* 14, (2022).

224. Xu, Z., Zhang, B., Liu, Z. & Gou, S. Design, synthesis and anticancer evaluation of selective 2,4-disubstituted pyrimidine CDK9 inhibitors. *Eur J Med Chem* 244, (2022).

225. Wu, S. *et al.* A synthesized olean-28,13β-lactam targets YTHDF1-GLS1 axis to induce ROS-dependent metabolic crisis and cell death in pancreatic adenocarcinoma. *Cancer Cell Int* 22, (2022).

226. Zhang, Y. *et al.* Hesperadin suppresses pancreatic cancer through ATF4/GADD45A axis at nanomolar concentrations. *Oncogene* 41, 3394–3408 (2022).

227. Pan, P. *et al.* HDAC5 Loss Enhances Phospholipid-Derived Arachidonic Acid Generation and Confers Sensitivity to cPLA2 Inhibition in Pancreatic Cancer. *Cancer Res* 82, 4542–4554 (2022).

228. Wu, C. *et al.* Gene Coexpression Network Characterizing Microenvironmental Heterogeneity and Intercellular Communication in Pancreatic Ductal Adenocarcinoma: Implications of Prognostic Significance and Therapeutic Target. *Front Oncol* 12, (2022).

229. Mao, T. *et al.* HDACs/mTOR inhibitor synergizes with pyrotinib in HER2-positive pancreatic cancer through degradation of mutant P53. *Cancer Cell Int* 22, (2022).

230. Okamoto, S. *et al.* Inhibition of pancreatic cancer-cell growth and metastasis in vivo by a pyrazole compound characterized as a cell-migration inhibitor by an in vitro chemotaxis assay. *Biomedicine and Pharmacotherapy* 155, (2022).

231. Zhao, C. *et al.* A self-amplifying USP14-TAZ loop drives the progression and liver metastasis of pancreatic ductal adenocarcinoma. *Cell Death Differ* 30, 1–15 (2022).

232. Cave, D. D. *et al.* LAMC2 marks a tumor-initiating cell population with an aggressive signature in pancreatic cancer. *Journal of Experimental and Clinical Cancer Research* 41, (2022).

233. Wang, X. *et al.* Mitochondrial Calcium Uniporter Drives Metastasis and Confers a Targetable Cystine Dependency in Pancreatic Cancer. *Cancer Res* 82, 2254–2268 (2022).

234. Brooun, A. *et al.* The pharmacologic and toxicologic characterization of the potent and selective KRAS G12D inhibitors ERAS-4693 and ERAS-5024. *Toxicol Appl Pharmacol* 474, (2023).

235. Chang, C. Di *et al.* In silico identification and biological evaluation of a selective MAP4K4 inhibitor against pancreatic cancer. *J Enzyme Inhib Med Chem* 38, (2023).

236. Chen, Y. *et al.* Design, synthesis and biological evaluation of novel DCLK1 inhibitor containing purine skeleton for the treatment of pancreatic cancer. *Eur J Med Chem* 261, (2023).

237. Morimoto, Y. *et al.* Simultaneous inhibition of Chk1 and Bcl-xL induces apoptosis *in vitro* and represses tumour growth in an *in vivo* xenograft model. *Journal of Chemotherapy* 35, 435–447 (2023).

238. Jeong, M.-H., Urquhart, G., Lewis, C., Chi, Z. & Jewell, J. L. Inhibition of phosphodiesterase 4D suppresses mTORC1 signaling and pancreatic cancer growth. *JCI Insight* (2023). doi:10.1172/jci

239. Entrialgo-Cadierno, R. *et al.* The phospholipid transporter PITPNC1 links KRAS to MYC to prevent autophagy in lung and pancreatic cancer. *Mol Cancer* 22, (2023).

240. Kong, W. *et al.* Azeliragon inhibits PAK1 and enhances the therapeutic efficacy of AKT inhibitors in pancreatic cancer. *Eur J Pharmacol* 948, (2023).

241. Jeong, S. *et al.* Streamlined DNA-encoded small molecule library screening and validation for the discovery of novel chemotypes targeting BET proteins. *Mol Ther Nucleic Acids* 32, 637–649 (2023).

242. Huang, N., Liao, P., Zuo, Y., Zhang, L. & Jiang, R. Design, Synthesis, and Biological Evaluation of a Potent Dual EZH2-BRD4 Inhibitor for the Treatment of Some Solid Tumors. *J Med Chem* 66, 2646–2662 (2023).

243. She, C. *et al.* Combination of RUNX1 inhibitor and gemcitabine mitigates chemo‐resistance in pancreatic ductal adenocarcinoma by modulating BiP/PERK/eIF2α-axis-mediated endoplasmic reticulum stress. *Journal of Experimental and Clinical Cancer Research* 42, (2023).

244. Lee, E. J. *et al.* A new vulnerability to BET inhibition due to enhanced autophagy in BRCA2 deficient pancreatic cancer. *Cell Death Dis* 14, (2023).

245. Lin, Q. *et al.* Expression of fibroblast growth factor receptor 1 correlates inversely with the efficacy of single-agent fibroblast growth factor receptor-specific inhibitors in pancreatic cancer. *Br J Pharmacol* 181, 1383–1403 (2024).

246. Stossel, C. *et al.* Spectrum of Response to Platinum and PARP Inhibitors in Germline BRCA–Associated Pancreatic Cancer in the Clinical and Preclinical Setting. *Cancer Discov* 13, 1826–1843 (2023).

247. Resovi, A. *et al.* Fibronectin fragments generated by pancreatic trypsin act as endogenous inhibitors of pancreatic tumor growth. *Journal of Experimental and Clinical Cancer Research* 42, (2023).

248. Feng, T. *et al.* PDZ-binding kinase aggravates pancreatic neuroendocrine neoplasm progression by activating the AKT/mTOR pathway. *Mol Carcinog* 62, 716–726 (2023).

249. Hamdan, F. H. *et al.* Interactive enhancer hubs (iHUBs) mediate transcriptional reprogramming and adaptive resistance in pancreatic cancer. *Gut* 72, 1174–11185 (2023).

250. Hering, N. A. *et al.* Targeting Interleukin-6/Glycoprotein-130 Signaling by Raloxifene or SC144 Enhances Paclitaxel Efficacy in Pancreatic Cancer. *Cancers (Basel)* 15, (2023).

251. Lee, M. *et al.* Venadaparib Is a Novel and Selective PARP Inhibitor with Improved Physicochemical Properties, Efficacy, and Safety. *Mol Cancer Ther* 22, 333–342 (2023).

252. Chen, H. *et al.* Selectively Targeting STAT3 Using a Small Molecule Inhibitor is a Potential Therapeutic Strategy for Pancreatic Cancer. *Clinical Cancer Research* 29, 815–830 (2023).

253. Li, D. *et al.* Scaffolding Protein Connector Enhancer of Kinase Suppressor of Ras 1 (CNKSR1) Regulates MAPK Inhibition Responsiveness in Pancreas Cancer via Crosstalk with AKT Signaling. *Molecular Cancer Research* 21, 316–331 (2023).

254. Xu, Y. *et al.* First-in-Class NADH/Ubiquinone Oxidoreductase Core Subunit S7 (NDUFS7) Antagonist for the Treatment of Pancreatic Cancer. *ACS Pharmacol Transl Sci* 6, 1164–1181 (2023).

255. Huang, L. *et al.* The suppressive efficacy of THZ1 depends on KRAS mutation subtype and is associated with super‐enhancer activity and the PI3K/AKT/mTOR signalling in pancreatic ductal adenocarcinoma: A hypothesis‐generating study. *Clin Transl Med* 13, (2023).

256. Guijarro, M. V *et al.* First-in-class multifunctional TYMS nonclassical antifolate inhibitor with potent in vivo activity that prolongs survival. (2023). doi:10.1172/jci

257. Kemp, S. B. *et al.* Efficacy of a Small-Molecule Inhibitor of KrasG12D in Immunocompetent Models of Pancreatic Cancer. *Cancer Discov* 13, 298–311 (2023).

258. Itzhak, I., Bareket-Samish, A. & Fishman, P. Namodenoson Inhibits the Growth of Pancreatic Carcinoma via Deregulation of the Wnt/β-catenin, NF-κB, and RAS Signaling Pathways. *Biomolecules* 13, (2023).

259. Kim, C. *et al.* PARP Inhibitor Sensitizes BRCA -mutant Pancreatic Cancer to Oxaliplatin by Suppressing the CDK1/BRCA1 Axis. *Anticancer Res* 43, 5523–5534 (2023).

260. Quiñonero, F. *et al.* Combining Olaparib and Ascorbic Acid on Nanoparticles to Enhance the Drug Toxic Effects in Pancreatic Cancer. *Int J Nanomedicine* 18, 5075–5093 (2023).

261. Kumar, K. *et al.* Targeting BET Proteins Decreases Hyaluronidase-1 in Pancreatic Cancer. *Cells* 12, (2023).

262. Ghukasyan, R. *et al.* MEK Inhibition Sensitizes Pancreatic Cancer to STING Agonism by Tumor Cell–intrinsic Amplification of Type I IFN Signaling. *Clinical Cancer Research* 29, 3130–3141 (2023).

263. Lee, M. R. *et al.* Application of plasma circulating KRAS mutations as a predictive biomarker for targeted treatment of pancreatic cancer. *Cancer Sci* 115, 1283–1295 (2024).

264. Zhang, C. di *et al.* Synergistic antitumor efficacy of rMV-Hu191 and Olaparib in pancreatic cancer by generating oxidative DNA damage and ROS-dependent apoptosis. *Transl Oncol* 39, (2024).

265. Lu, D. *et al.* Lead Compound Development of SRC-3 Inhibitors with Improved Pharmacokinetic Properties and Anticancer Efficacy. *J Med Chem* 67, 5333–5350 (2024).

266. Li, M. *et al.* GK921, a transglutaminase inhibitor, strengthens the antitumor effect of cisplatin on pancreatic cancer cells by inhibiting epithelial-to-mesenchymal transition. *Biochim Biophys Acta Mol Basis Dis* 1870, (2024).

267. Ferretti, G. D. S. *et al.* HSP70-mediated mitochondrial dynamics and autophagy represent a novel vulnerability in pancreatic cancer. *Cell Death Differ* 31, 881–896 (2024).

268. Erdem, S. *et al.* Inhibition of SUMOylation Induces Adaptive Antitumor Immunity against Pancreatic Cancer through Multiple Effects on the Tumor Microenvironment. *Mol Cancer Ther* 23, 1597–1612 (2024).

269. Ayaz, M. O. *et al.* Identification of a novel GSK3β inhibitor involved in abrogating KRas dependent pancreatic tumors in Wnt/beta-catenin and NF-kB dependent manner. *Life Sci* 351, (2024).

270. Gu, J. *et al.* Ubiquitin-specific protease 7 maintains c-Myc stability to support pancreatic cancer glycolysis and tumor growth. *J Transl Med* 22, (2024).

271. Becker, J. H. *et al.* Targeting BCL2 with Venetoclax Enhances the Efficacy of the KRASG12DInhibitor MRTX1133 in Pancreatic Cancer. *Cancer Res* 84, 3629–3639 (2024).

272. Su, D. *et al.* FBXO32 Stimulates Protein Synthesis to Drive Pancreatic Cancer Progression and Metastasis. *Cancer Res* 84, 2607–2625 (2024).

273. Miyazaki, S. *et al.* Targeting KRAS-mutant pancreatic cancer through simultaneous inhibition of KRAS, MEK, and JAK2. *Mol Oncol* 19, 377–390 (2025).

274. Pecoraro, C. *et al.* Exploring the therapeutic potential of a novel series of imidazothiadiazoles targeting focal adhesion kinase (FAK) for pancreatic cancer treatment: synthesis, mechanistic insights and promising antitumor and safety profile. *J Drug Target* 32, 1278–1294 (2024).

275. Li, H., Yao, Y., Hao, R. & Long, C. Selective and effective suppression of pancreatic cancer through MNK inhibition. *Immunopharmacol Immunotoxicol* 46, 651–661 (2024).

276. Kumarasamy, V. *et al.* The Extracellular Niche and Tumor Microenvironment Enhance KRAS Inhibitor Efficacy in Pancreatic Cancer. *Cancer Res* 84, 1115–1132 (2024).

277. Hawkins, H. J. *et al.* Examination of Wnt signaling as a therapeutic target for pancreatic ductal adenocarcinoma (PDAC) using a pancreatic tumor organoid library (PTOL). *PLoS One* 19, (2024).

278. Reichardt, W. *et al.* 19Fluorine-MRI Based Longitudinal Immuno-Microenvironment-Monitoring for Pancreatic Cancer. *Journal of Magnetic Resonance Imaging* 61, 1996–2008 (2025).

279. Gao, Z. *et al.* Sphingosine-1-Phosphate Inhibition Increases Endoplasmic Reticulum Stress to Enhance Oxaliplatin Sensitivity in Pancreatic Cancer. *World J Oncol* 15, 169–180 (2024).

280. Zhou, C. *et al.* Anti-tumor efficacy of HRS-4642 and its potential combination with proteasome inhibition in KRAS G12D-mutant cancer. *Cancer Cell* 42, 1286-1300.e8 (2024).

281. Zarei, M. *et al.* IDH1 Inhibition Potentiates Chemotherapy Efficacy in Pancreatic Cancer. *Cancer Res* 84, 3072–3085 (2024).

282. Xiao, M. *et al.* NLRP4 renders pancreatic cancer resistant to olaparib through promotion of the DNA damage response and ROS-induced autophagy. *Cell Death Dis* 15, (2024).

283. Ge, W. *et al.* Activation of the PI3K/AKT signaling pathway by ARNTL2 enhances cellular glycolysis and sensitizes pancreatic adenocarcinoma to erlotinib. *Mol Cancer* 23, (2024).

284. Wasko, U. N. *et al.* Tumour-selective activity of RAS-GTP inhibition in pancreatic cancer. *Nature* 629, 927–936 (2024).

285. Ku, B. *et al.* PRMT1 promotes pancreatic cancer development and resistance to chemotherapy. *Cell Rep Med* 5, (2024).

286. Xiao, M. *et al.* TPX2 serves as a novel target for expanding the utility of PARPi in pancreatic cancer through conferring synthetic lethality. *Pancreas* 74, 410–423 (2024).

287. Jiang, J. *et al.* Translational and Therapeutic Evaluation of RAS-GTP Inhibition by RMC-6236 in RAS-Driven Cancers. *Cancer Discov* 14, 994–1017 (2024).

288. Wang, S. *et al.* Genome-wide CRISPR screens identify PKMYT1 as a therapeutic target in pancreatic ductal adenocarcinoma. *EMBO Mol Med* 16, 1115–1142 (2024).

289. Singhal, A. *et al.* A Classical Epithelial State Drives Acute Resistance to KRAS Inhibition in Pancreatic Cancer. *Cancer Discov* 14, 2122–2134 (2024).

290. Xu, X. *et al.* Discovery of a potent and highly selective inhibitor of SIRT6 against pancreatic cancer metastasis in vivo. *Acta Pharm Sin B* 14, 1302–1316 (2024).

291. Li, Y. *et al.* Design, synthesis and pharmacological evaluation of 1,2,3,4-tetrahydrobenzofuro[2,3-c]pyridine derivatives as p21-activated kinase 4 inhibitors for treatment of pancreatic cancer. *Acta Pharm Sin B* 15, 438–466 (2025).

292. Zeng, C. Y. *et al.* YKL-06-061 exerts antitumor effect through G1/S phase arrest by downregulating c-Myc and inhibition of metastasis via SIK1 upregulation in pancreatic cancer. *Anticancer Drugs* 36, 114–125 (2025).

293. Chun, J. W. *et al.* ATR inhibition promotes synergistic antitumor effect in platinum-resistant pancreatic cancer. *Biochim Biophys Acta Mol Basis Dis* 1871, (2025).

294. Jobu, Y. *et al.* Inhibitory effects of the combination of rapamycin with gemcitabine plus paclitaxel on the growth of pancreatic cancer tumors. *Hum Cell* 38, (2025).

295. Yao, Z. *et al.* Niraparib perturbs autophagosome-lysosome fusion in pancreatic ductal adenocarcinoma and exhibits anticancer potential against gemcitabine-resistant PDAC. *Transl Oncol* 51, (2025).

296. Zhang, Z. *et al.* Inhibition of KLF5 promotes ferroptosis via the ZEB1/HMOX1 axis to enhance sensitivity to oxaliplatin in cancer cells. *Cell Death Dis* 16, (2025).

297. Addassi, H. A. *et al.* Inhibition of phospholipase D1 reduces pancreatic carcinogenesis in mice partly through a FAK-dependent mechanism. *Carcinogenesis* 46, (2025).
